# Post-fabrication reconfiguration of hydrogels through thermodynamic asymmetry in dynamic chemistry

**DOI:** 10.1038/s41467-026-78292-4

**Published:** 2026-10-07

**Authors:** Davide Campagna, Diego Ciardi, Weixiang Chen, Andreas Walther

**Affiliations:** Life-Like Materials and Systems, University of Mainz, Mainz, Germany

**Keywords:** Self-assembly, Mechanical properties, Self-assembly

## Abstract

Designing soft materials with adaptive properties requires internal chemical processes that enable reconfiguration across multiple system levels. Dynamic covalent chemistry has provided powerful routes to dynamic polymer networks, yet it is typically employed in an equilibrium-based manner where the identity of the dynamic bond remains unchanged. Here, we introduce thermodynamic asymmetry in dynamic covalent chemistry to enable the replacement of network-defining linkages after material formation, rather than their mere bond shuffling. For combinations of hydrazones and oximes, we demonstrate near-quantitative hydrazone-to-oxime exchange under aqueous conditions. Kinetic modeling elucidates that the exchange is dominated by a hydrolytic pathway with a non-trivial concentration dependence. This chemistry enables diverse reconfigurations within hydrogels after their initial synthesis, achieving near-complete replacement at material scale. Treating pre-formed hydrazone gels with functional alkoxyamines enables topological, mechanical, and functional reprogramming without network deconstruction. This work establishes thermodynamically biased dynamic covalent exchange as a generalizable principle for materials, surface functions, or self-assembling systems, illustrating how molecular reaction pathways can be harnessed to deterministically alter structural and functional identity.

## Abstract (web summary)

Dynamic covalent chemistry has provided powerful routes to dynamic polymer networks, yet the nature of the dynamic bond is typically defined at the outset and remains unchanged. Here, the authors introduce thermodynamic asymmetry in dynamic covalent chemistry to enable the reconfiguration of network-defining linkages after material formation, allowing for functional material evolution.

## Introduction

Soft materials that can adapt their structure and function are central to emerging concepts in biomaterials, tissue engineering, life-like and intelligent matter^1,2^. Hydrogels, in particular, provide a versatile platform to emulate the dynamic and heterogeneous environments found in biological systems, where material properties evolve in response to chemical cues. While numerous strategies exist to tune hydrogel properties during fabrication or to endow gels with reversible stimulus-responsive behavior^3–9^, these approaches often modulate an existing network rather than replacing the chemical identity of the network-defining crosslinks. A more demanding form of post-fabrication reprogramming is the ability to alter network topology, mechanics, or function after material formation by exchanging the molecular motifs that constitute the network itself.

In the field of dynamic covalent chemistry, the primary focus has been on exploiting reversible bond exchange to endow polymer networks with properties such as self-healing, stress relaxation, and stimuli-responsive mechanics^10,11^. In these systems, the dynamic covalent motif is typically selected at the outset and remains unchanged throughout the material lifetime, with bond exchange occurring between identical functional groups. Thus, dynamic exchange is commonly used to rearrange existing bonds, rather than to replace one class of network-forming linkage with another in a directional and predictable manner.

Achieving more complex reprogramming requires more than reversibility. It demands directionality and selectivity in bond exchange. Specifically, a mechanism is needed that biases exchange toward one bonding motif over another, enabling predictable and near-quantitative exchange rather than stochastic equilibration. While individual dynamic covalent reactions have been extensively characterized in solution, general strategies that translate molecular-level exchange into controlled reconfiguration of polymer networks are scarce^12,13^, and include the use of the Diels-Alder system undergoing diene displacement that requires harsh conditions (i.e., heating at 120 °C)^14^, or a specific conjugate acceptor undergoing a thiol-amine acyl substitution under mild conditions, but lacking specificity of the exchangeable motif^15^. Consequently, versatile design principles for chemically reprogramming soft materials after formation are missing. Such a strategy would connect molecular reaction pathways directly to predictable material-level reprogramming.

We hypothesized that imine-based chemistries may offer a particularly attractive platform to address this challenge, as they offer diverse application scenarios and their equilibrium properties can be systematically tuned^16–27^. Differences in hydrolytic stability between related imine derivatives introduce intrinsic thermodynamic biases that, in principle, can be harnessed to drive directional bond exchange. However, their potential as active chemical design elements, and the connection between molecular exchange pathways and macroscopic material reconfiguration, remain poorly understood.

Here, we introduce a dynamic covalent chemistry strategy that exploits the thermodynamic asymmetry between hydrazone and oxime linkages to enable directional exchange in covalent polymer hydrogel networks. The key feature is the near-quantitative replacement of hydrazone crosslinking segments by oxime linkages after network formation, rather than reversible bond shuffling within a single linkage class. We define a kinetic framework for this exchange at the molecular level and show how the identity of the dynamic linkage governs gelation kinetics, stress relaxation, and network topology. Diffusion of reactive alkoxyamine species into pre-formed materials enables crosslink replacement, which in turn allows programmable mechanical reconfiguration and the introduction of new modes of responsiveness. This approach provides a general framework for chemically reprogramming soft materials after formation.

## Results

### Quantitative assessment of the exchange from hydrazone to oxime

Our strategy for reconfigurable hydrogels exploits a fundamental thermodynamic asymmetry within imine chemistry, specifically between hydrazones and oximes. Both linkages, hydrazones and oximes, are formed by condensation of aldehydes with their respective α-nucleophiles, hydrazines and alkoxyamines and differ markedly in their stability toward hydrolysis in water. The substituent in the α-position strongly influences the equilibrium of imine hydrolysis (*K*_d_ of hydrolysis). Lower basicity and nucleophilicity correlate with increased bond stability^28^. As a consequence, oximes are hydrolytically more stable than hydrazones.

**Fig. 1:**
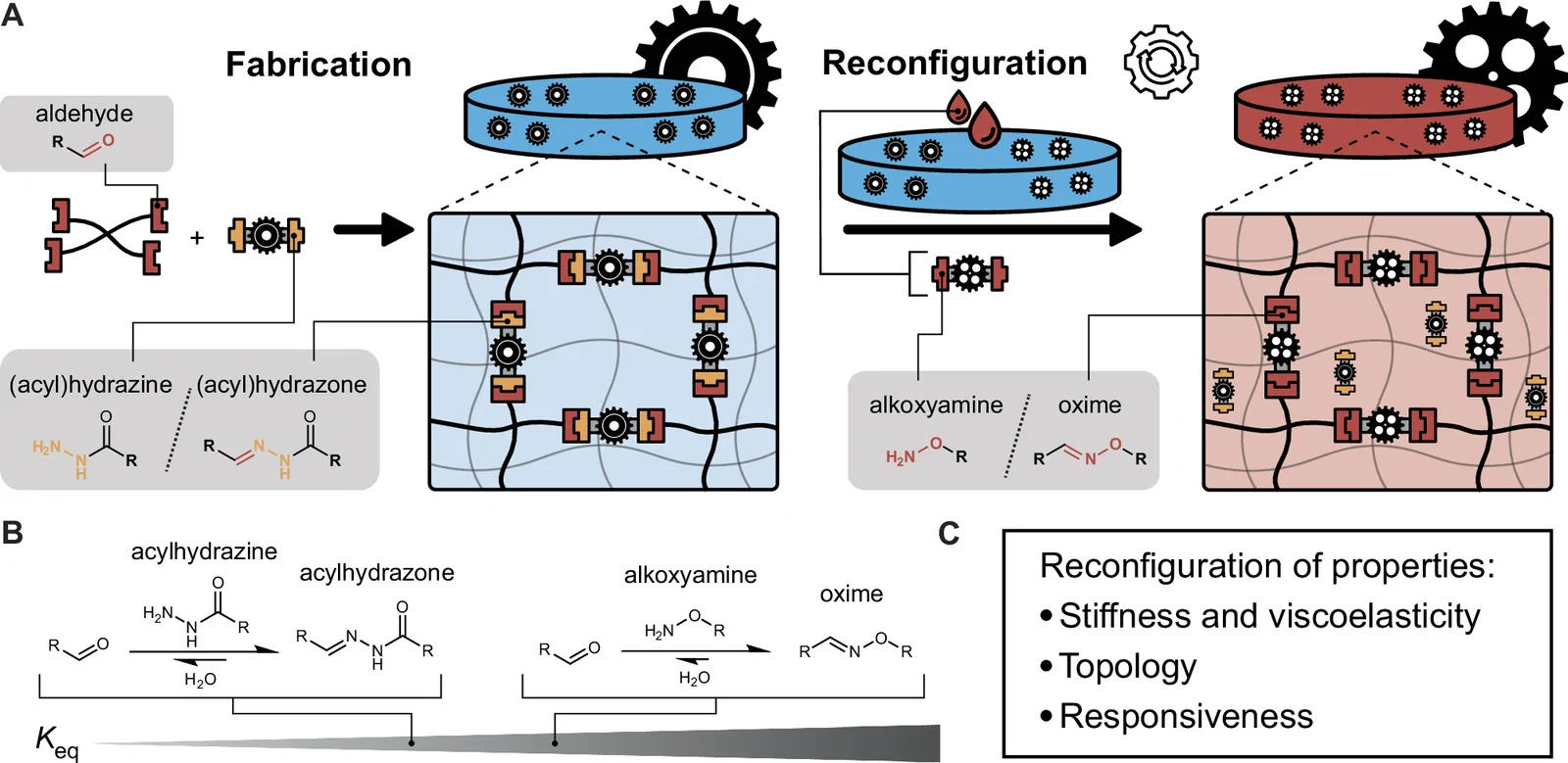
Overview of the concept of functional reconfiguration of hydrogel networks by hydrazone-to-oxime exchange. **A** Fabrication of hydrazone-crosslinked hydrogels, and subsequent reconfiguration of the crosslinking segments by addition of alkoxyamines, leading to an oxime-crosslinked hydrogel. The different gears symbolize different functionalities arising from this exchange. **B** Thermodynamic imbalance between two types of imines: (acyl)hydrazone and oxime. **C** Summary of the reconfigurations of the hydrogel properties achieved in this work.

While hydrazones and oximes have individually been employed in dynamic covalent systems^29–34^, the implications of their intrinsic thermodynamic asymmetry for directional bond exchange in multicomponent systems remain unrecognized. Only a few isolated observations of this exchange exist, without mechanistic analysis or materials-level implications^35,36^. Here, we leverage this imbalance to establish hydrazone-to-oxime exchange as a predictable and efficient chemical operator for network reconfiguration, motivating a quantitative investigation of its kinetics and mechanism as a prerequisite for materials design (Fig. 1).

**Fig. 2:**
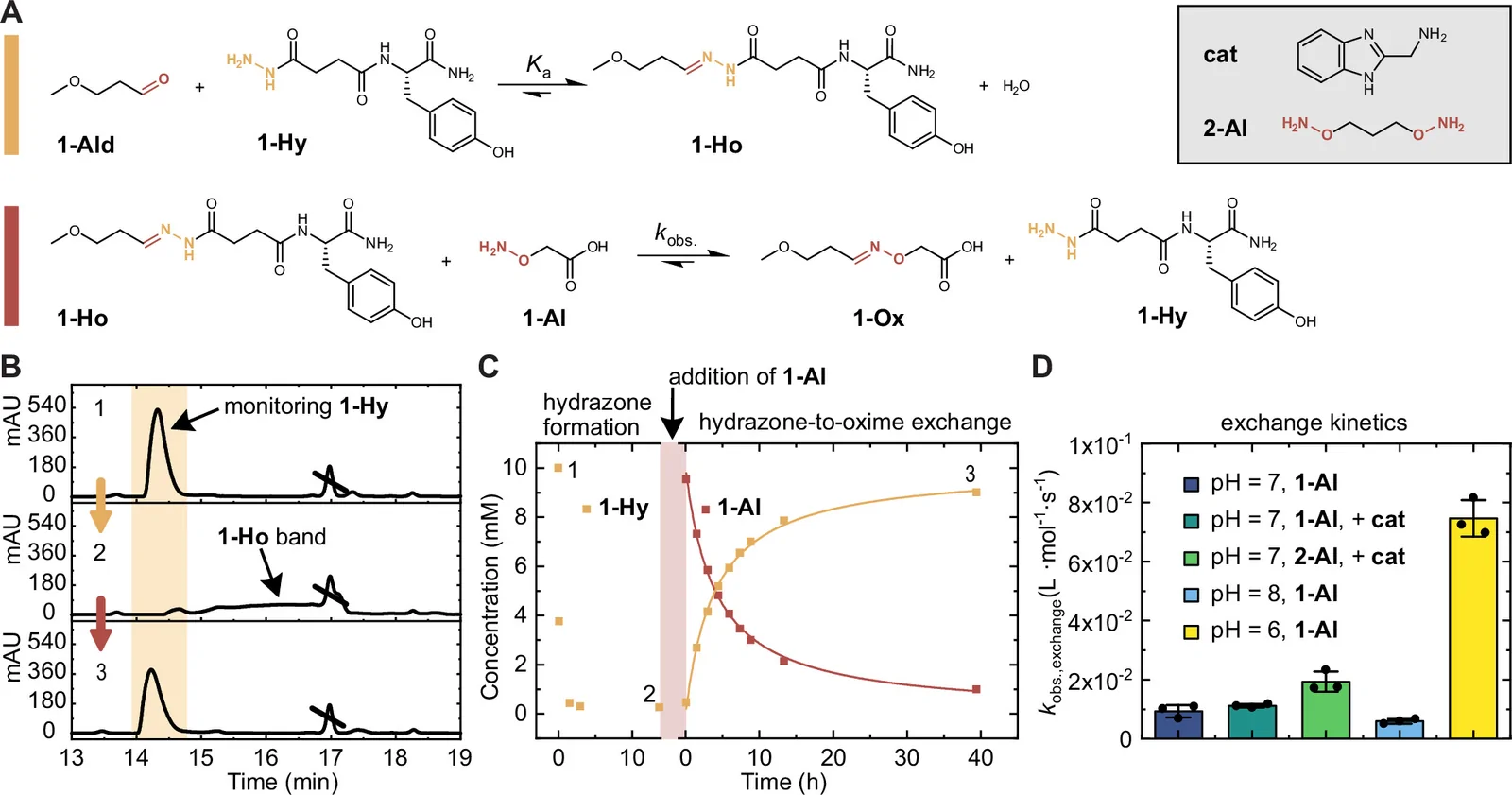
Small molecule model studies to understand the hydrazone-to-oxime exchange. **A** Reaction schemes for hydrazone formation and subsequent exchange to oxime. **B** Exemplary HPLC chromatograms of (top-to-bottom) 1-Hy before reaction, hydrazone 1-Ho formation after 14 h (the reaction was left overnight to ensure full equilibration), and exchange reaction to oxime by adding 1-Al, after 40 h. At this reaction time, the exchange is near-quantitative (impurities indicated with black lines; 1-Ho has a broad band due to its dynamic nature, with hydrolysis occurring in the HPLC column). Elution was performed using a gradient water/acetonitrile from 95:5 to 5:95 ratio (v/v), followed by isocratic 0.1% trifluoroacetic acid (TFA) in water/acetonitrile 95:5 ratio (v/v). **C** Quantified time courses from HPLC data. Lines are fits to second-order irreversible kinetics. Conditions for (**B**, **C**): 10 mM 1-Ald + 10 mM 1-Hy; final product reacted with 10 mM 1-Al; 100 mM PBS, pH 7, without catalyst. **D** *k*_obs_ for the exchange reaction under different conditions, as indicated. Tabulated values and details are in Table S1. *n* = 3. Bars show mean values. Error bars represent standard deviations.

We first focus on deriving quantitative information on the hydrazone-to-oxime exchange using small model molecules by HPLC under different conditions (detailed list of experiments in Table S1, complete reaction schemes in Fig. S1). We synthesized a hydrazine-functionalized tyrosinamide derivative, 1-Hy, with excellent water solubility and suitable UV absorbance for HPLC detection. We then carried out sequential reactions. First, formation of the hydrazone 1-Ho by condensation between hydrazine 1-Hy and aldehyde 1-Ald in equimolar ratio, followed by exchange between the formed hydrazone 1-Ho and an equivalent amount of the alkoxyamine 1-Al (Fig. 2A, pH 7, 100 mM PBS). The HPLC chromatograms clearly show the disappearance of the peak of the hydrazine 1-Hy upon condensation with the aldehyde 1-Ald, and its reappearance after the addition of the alkoxyamine 1-Al (Fig. 2B). Formation of the hydrazone 1-Ho proceeds quickly. A plateau is reached in less than two hours. The following exchange reaction to oxime 1-Ox, with release of the original hydrazine 1-Hy, occurs significantly more slowly but near-quantitatively (Fig. 2C). Both reactions reflect equilibration reactions.

While the hydrazone formation is governed by reversible kinetics, which depends on the association equilibrium constant (*K*_a_), the oxime produced via the exchange reaction is characterized by exceptional hydrolytic stability^28^, and the corresponding curve can be fitted to second-order irreversible kinetics (red curve), obtaining a *k*_obs, exchange_. The exchange kinetics depend on environmental parameters and substrate specifics (Fig. 2D, kinetic fits in Fig. S2). The addition of an amine-bearing catalyst (cat)^37^ shows only a mild acceleration by 19 %, whereas changing the alkoxyamine 1-Al to 2-Al has a stronger influence, showing an acceleration by 106 %, due to the differences in the electronic nature of the reactive group. The effect of pH changes dramatically from slightly basic to acidic conditions. A reduction to pH 6 greatly accelerates the exchange kinetics by almost one order of magnitude through acid catalysis^30^, while an increase to pH 8 does not show acceleration; on the contrary, it shows a mild decrease by 37%. Overall, the exchange proceeds efficiently in all conditions, underscoring the robustness of the process.

### Definition of a kinetic framework and elucidation of the mechanism

After quantifying the exchange under different aqueous conditions, we next clarify the mechanism and define an appropriate kinetic framework. Transiminations are well documented for conventional primary amines and can proceed either via a direct associative pathway or through a hydrolytic dissociative pathway. The reaction has been deeply studied in organic solvents^38^, where it occurs by the direct pathway via an aminal tetrahedral intermediate. In heterotransiminations, the thermodynamic imbalance biases the equilibrium towards the imine that incorporates the most basic amine^38^.

In aqueous conditions, however, the situation is more complex due to the possible hydrolytic pathway, and no unified mechanistic framework exists in the literature. Hydrolytic transimination involves transient aldehyde intermediates, and, in competitive settings, favors the formation of the imine that incorporates the least basic amine, as this bond has a higher stability toward hydrolysis. The possible co-existence of both mechanisms therefore complicates the kinetic description of the exchange process.

**Fig. 3:**
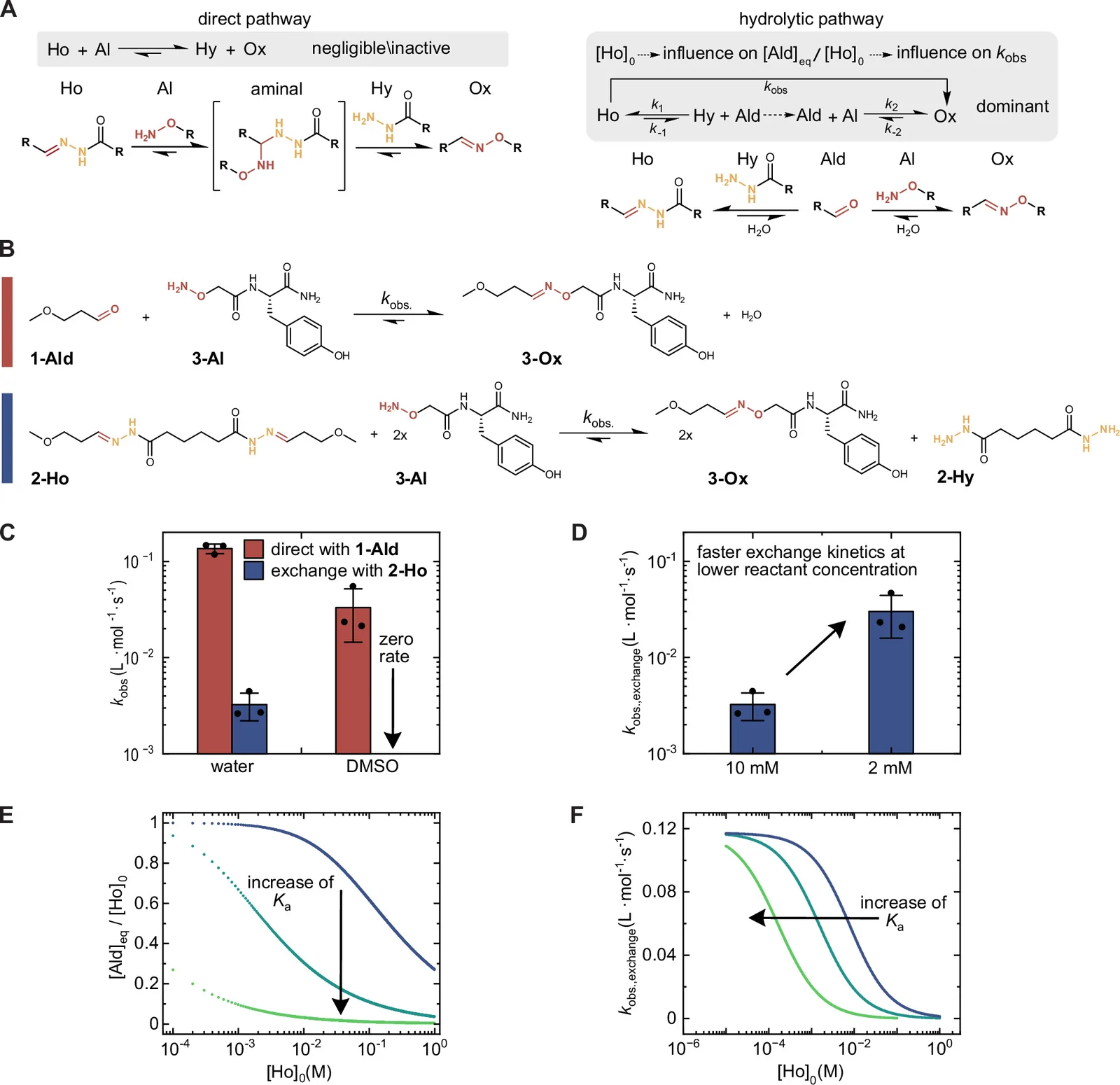
Mechanistic insights into hydrazone-to-oxime exchange chemistry. **A** Schemes of direct versus hydrolytic exchange pathway. **B** Reaction schemes of the direct oxime formation and exchange using 3-Al for a comparative analysis to extract mechanistic information. Note that during the exchange reaction, a mono-hydrazone product is transiently formed. **C** *k*_obs_ obtained by fitting to second-order irreversible kinetics for the respective reactions in (**B**) conducted at stoichiometric balance at 10 mM (pH 7, 100 mM PBS; details in Table S1). **D** Influence of reactant concentration on the hydrazone-to-oxime exchange kinetics (pH 7, 100 mM PBS; details in Table S1). (C-D) *n* = 3. Bars show mean values. Error bars represent standard deviations. **E** Analytical solution of the ratio [Ald]_eq_ / [Ho]_0_ against different values of [Ho]_0_, for three different values of *K*_a_ (L∙mol^-1^): 0.74 ∙ 10^3^ (determined experimentally), 10^5^ and 10^2^ (chosen arbitrarily). The fraction of free aldehyde increases by lowering the concentration of initial hydrazone. **F** Simulated *k*_obs_ of hydrazone-to-oxime exchange against [Ho]_0_ (equal to [Al]_0_), neglecting any back reaction, *k*_-2_, and fitted to the logistic function for three different values of *K*_a_ (L∙mol^−1^): 0.74 ∙ 10^3^ (determined experimentally), 0.74 ∙ 10^4^ and 0.74 ∙ 10^2^ (chosen arbitrarily by varying *k*_1_, see Table S2).

For our hydrazone-oxime system, Fig. 3A summarizes both mechanistic pathways, while Supplementary Note 1 provides a full kinetic model (scheme in Fig. S3). Generic aldehyde, hydrazine, alkoxyamine, hydrazone, and oxime species are abbreviated as Ald, Hy, Al, Ho, and Ox, respectively. The direct (associative) pathway admits an analytical solution under equimolar conditions of Ho and Al, whereas the hydrolytic (dissociative) pathway requires a coupled set of ordinary differential equations, which must be solved numerically (Supplementary Note 1).

To identify the predominant exchange pathway, we carried out a set of comparative reactions designed to separate the direct from the hydrolytic pathway. First, we compare direct oxime formation and hydrazone-to-oxime exchange in water (pH 7, 100 mM PBS) and in DMSO, in the absence of catalyst. For this purpose, we synthesized alkoxyamine 3-Al that can react with an equivalent amount of either aldehyde 1-Ald or hydrazone 2-Ho (Fig. 3B). 2-Ho was made independently by reacting equimolar 2-Hy and 1-Ald (Fig. S4). In both cases, oxime 3-Ox is the final product.

While the oxime formation between 1-Ald and 3-Al proceeds readily in DMSO, without catalyst, at a rate comparable to that in water, no hydrazone-to-oxime exchange between 2-Ho and 3-Al is detected in DMSO. This contrasts with water, where a clear hydrazone-to-oxime exchange occurs (Fig. 3C, exemplary chromatogram and kinetic fits in Figure S5). The absence of exchange in DMSO persists even in the presence of catalyst or minor fractions of deionized water, 1 % and 5% v/v (Fig. S6). Since DMSO strongly suppresses hydrolytic pathways, exchange under these conditions would be expected to proceed predominantly through a direct pathway.

To further elucidate the dominance of the hydrolytic pathway, we adopted a kinetics-based approach. We performed the hydrazone-to-oxime exchange between 2-Ho and 3-Al in water (pH 7, 100 mM PBS) without catalyst, at two different initial concentrations [2-Ho]_0_ = [3-Al]_0_ = 2 and 10 mM, yielding markedly different observed rate constants, *k*_obs_. Counterintuitively, a higher *k*_obs_ is obtained at lower concentration (Fig. 3D).

This behavior can be rationalized by considering the aldehyde-hydrazone equilibrium in water. Aldehyde and hydrazine condense to form a hydrazone via a second-order reaction, while hydrolysis back to the reactants proceeds via a pseudo-first-order reaction. The difference in reaction order leads to distinct concentration dependencies of formation and hydrolysis rates, and consequently to different equilibrium ratios of aldehyde and hydrazone as a function of the initial hydrazone concentration, [Ho]_0_. This effect can be explicitly described for the hydrolysis of an initially pure hydrazone, yielding a ratio [Ald]_eq_/[Ho]_0_ that varies with [Ho]_0_ following a sigmoidal dependence (Fig. 3E). The shape of this curve is governed by the equilibrium constant of the aldehyde-hydrazone system expressed as *K*_a_ = [Ho]_eq_/([Ald]_eq_ · [Hy]_eq_). In a hydrolytic exchange pathway, the transient aldehyde fraction directly determines the amount of aldehyde available to react with alkoxyamine to form the final oxime (Fig. 3A). Accordingly, the observed dependence of *k*_obs_ on the initial concentration [Ho]_0_ = [Al]_0_ provides strong evidence that hydrazone-to-oxime exchange proceeds predominantly via the hydrolytic pathway.

To further validate the hydrolytic pathway as the dominant mechanism, we then carried out systematic kinetic simulations that account for hydrazone hydrolysis and subsequent oxime formation (Supplementary Note 1). The model relates *k*_obs_ to [Ho]_0_ based on reactions and rate constants for hydrazone formation and hydrolysis (*k*_1_ and *k*_-1_) and oxime formation (*k*_2_), while neglecting oxime hydrolysis (*k*_*-*2_) due to the high hydrolytic stability of oximes under the conditions studied (Supplementary Note 1). Experimentally determined rate constants were used as input parameters to assess the consistency between simulations and measurements. The rate constants *k*_1_ and *k*_-1_ were determined by monitoring the hydrazone formation (2-Ho) by UV-Vis spectroscopy and fitting the data to a second-order reversible kinetic model (Fig. S7). These parameters correctly reproduce the experimentally observed dependence of the aldehyde-hydrazone equilibrium on concentration (Fig. S8). *k*_2_ was determined as *k*_obs_ for the direct oxime formation between 1-Ald and 3-Al to 3-Ox at pH 7 without catalyst (Fig. S5B, fit to second-order irreversible kinetics).

By applying the starting condition of [Ho]_0_ = [Al]_0_ = 10 mM, an exemplary simulation of the exchange reaction yields a time-dependent consumption profile of the alkoxyamine Al (Fig. S9A), whose time scale resembles the experimentally observed kinetics in Fig. 2C. This agreement confirms that the experimentally derived rate constants and our model capture relevant kinetics of the system. To systematically explore the influence of individual kinetic parameters, further simulations were carried out by varying the rate constants *k*_1_, *k*_-1_, *k*_2_. For each parameter, two additional values were chosen that are one order of magnitude lower and higher than the experimentally determined values (all values summarized in Table S2). For each parameter set, multiple simulations at varying [Ho]_0_ were performed and fitted to second-order irreversible kinetics to extract a series of apparent rate constants *k*_obs_. This procedure allows prediction of *k*_obs_ against [Ho]_0_ for different combinations of *k*_1_, *k*_-1_, *k*_2_ (Fig. 3F), yielding sigmoidal curves that can be described by a four-parameter logistic function of the form:

1 $${k}_{{{{\rm{obs}}}}}=\frac{{k}_{2}}{1+{\left(\frac{{\left[{{{\rm{Ho}}}}\right]}_{0}}{a}\right)}^{b}}$$

The curves asymptotically approach *k*_2_ at low [Ho]_0_, whereas *k*_obs_ approaches zero at high [Ho]_0_. This behavior is consistent with experiments, wherein *k*_obs_ decreases with increasing [Ho]_0_ (Fig. 3D). *b* is a constant value that determines the slope of the curve, while *a* contains the kinetic parameters *k*_1_, *k*_-1_, *k*_2_, and determines the inflection point. A detailed analysis can be found in Fig. S9B–F and further discussion in Supplementary Note 1. Finally, we note that while a minor contribution from a direct associative pathway cannot be formally excluded under all conditions, the combined DMSO control experiments, concentration dependence, and kinetic simulations indicate that the exchange is dominated by a hydrolytically mediated pathway under the aqueous conditions used here.

As an illustrative example, *k*_1_ was varied at fixed *k*_-1_ to simulate different values of *K*_a_. Increasing *K*_a_ shifts the sigmoidal dependence toward lower [Ho]_0_ values (Fig. 3F), resulting in lower *k*_obs_ at a given concentration. This shift reflects the lower fraction of transient aldehyde available to react with the alkoxyamine, in direct agreement with the hydrolysis-dominated mechanism.

### Gelation kinetics and mechanical properties of hydrogels

**Fig. 4:**
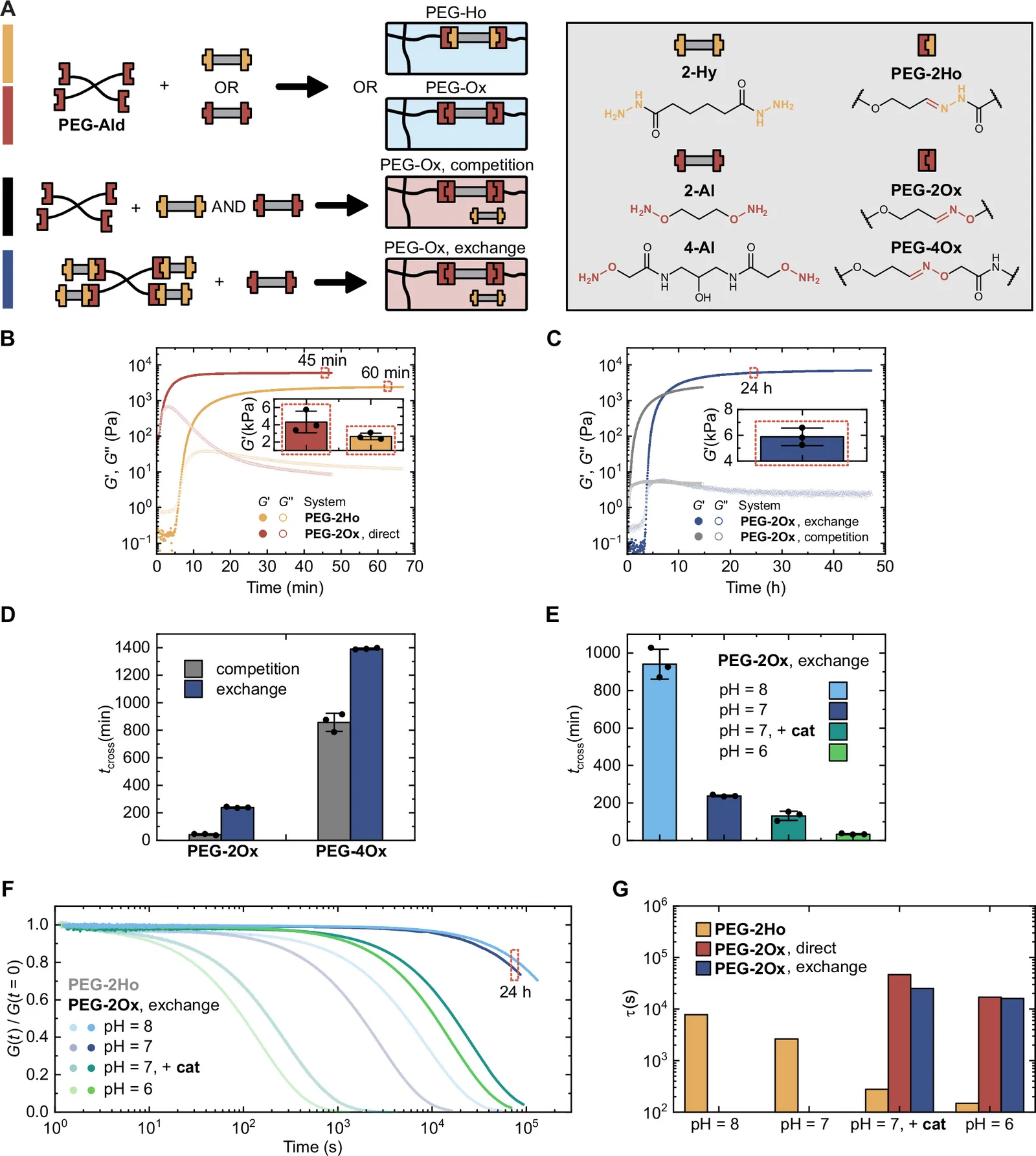
Gelation and exchange gelation in hydrogels and their dynamic stress-relaxation properties. **A** Scheme of the different gelation processes to fabricate gels (left), and box showing the corresponding molecular structures of the components (right). Comparative time sweeps for gel formation focusing on **B** PEG-2Ho and PEG-2Ox (direct), and **C** PEG-2Ox via competition and exchange (9.8 wt% PEG-Ald, 1 equivalent of respective crosslinker, 100 mM PBS, pH 7, no catalyst). Gelation via exchange is the slowest in each case. The insets show precise values of *G*′ at the indicated times (dotted red frames). *n* = 3. Bars show mean values. Error bars represent standard deviations. **D** Influence of alkoxyamine structure and gelation method on the timescale of gelation, at pH 7. **E** Influence of catalyst and pH on timescale of gelation via exchange, for PEG-2Ox. The trend is in line with the small-molecule experiments (see Fig. 2D). The gel point *t*_cross_ is defined as the time at which *G*′ = *G*″. *n* = 3. Bars show mean values. Error bars represent standard deviations. **F** Stress relaxation curves of hydrogels show vastly reduced stress relaxation for oxime gels vs hydrazone gels. **G** Characteristic relaxation times determined from **F**, using the single-mode exponential decay. Tabulated values and details are in Table S3.

We next transfer our molecular-level understanding to hydrogel formation of 4-arm poly(ethylene glycol)-aldehyde (PEG-Ald) with bivalent crosslinkers, enabling direct validation between molecular exchange kinetics, gelation behavior, and macroscopic mechanical properties derived from rheology. First, we discuss a series of gelation experiments conducted in 100 mM PBS at pH 7 in the absence of catalyst (Fig. 4A–C, details in Table S3). We compare (1) direct formation of the hydrazone- and oxime-crosslinked hydrogels (PEG-Ho and PEG-Ox), (2) competitive gelation using a mixture of hydrazine and alkoxyamine crosslinkers to PEG-Ald (1 equivalent of each crosslinker), and (3) gel formation by exchange, in which an alkoxyamine crosslinker (1 equivalent) is added to PEG-Ald that was pre-treated with a twofold excess of hydrazine crosslinker to generate hydrazone-terminated end-groups.

Looking first at the general trends, direct gelation of PEG-Ald with either the hydrazine 2-Hy or alkoxyamine 2-Al crosslinkers (orange, red curves in Fig. 4B) proceeds rapidly with *G*′/*G*″crossovers within 10 min after mixing. The oxime gel reaches a superior *G*′, consistent with more complete effective crosslink formation in its network, in line with the higher hydrolytic stability of oxime^39^. The oxime gel also forms faster than the hydrazone gel. Conversely, in the small-molecule study, the rate constant of the hydrazone formation (*k*_1_ ≈ 0.6 L·mol^−^^1^ · s^−1^, see Figure S7) is ca. 6-fold higher than the apparent rate constant of the oxime formation (*k*_obs_ ≈ 0.1 L·mol^−1^ · s^−1^, see entry 2 in Table S1). However, the much higher hydrolysis rate of the hydrazone (*k*_-1_ ≈ 0.0009 s^−1^, see Fig. S7), plays the key role in slowing down the overall gelation kinetics by concurrent breakage of already formed hydrazone linkages. Competitive gelation experiments (grey curve in Fig. 4C) employing both crosslinkers take an intermediate position, whereas the exchange gelation (blue curve in Fig. 4C) from hydrazone-terminated PEG-Ald using addition of alkoxyamine crosslinker 2-Al is the slowest, requiring close to 5 h to reach the gel point. These behaviors are in line with expectations, considering that direct reactions are faster than competitive reactions requiring partial exchange of intermediately formed hydrazones or even full reconfiguration when using pre-formed hydrazone-terminated PEG-Ald. In all the formations of oxime gels, the alkoxyamine 2-Al gives faster gelation kinetics than alkoxyamine 4-Al, which is due to the electronic differences of the reactive group, as previously observed for the molecular exchange kinetics (Fig. 4D, rheology in Fig. S10).

A correspondence between molecular exchange kinetics and hydrogel formation emerges clearly from the effects of catalyst and pH. For PEG-2Ox, the presence of catalyst almost halves the gelation time, which is dramatically reduced at pH 6 by roughly one order of magnitude, and increases at pH 8 approximately 4-fold (Fig. 4E, values in Table S3, rheology in Fig. S10). Overall, these results underscore a trend that aligns with molecular kinetics discussed above.

Importantly, all pathways lead to final hydrogels with roughly the same *G*′ plateau, which corresponds to similar effective crosslinking densities. This is an important observation, because it confirms that the incorporation of the alkoxyamine crosslinker is strongly favored over the hydrazine crosslinker. In particular, exchange gelation of prehybridized hydrazone blockers underscores a continuous and efficient replacement of all hydrazones with oximes.

Further critical insights regarding bond dynamics and hydrolysis can be derived from stress relaxation experiments (Fig. 4F). Hydrazone gels PEG-2Ho exhibit pronounced stress relaxation, whereas oxime gels PEG-2Ox show substantially diminished stress relaxation, rather independent of whether prepared by exchange or directly (see also Fig. S11A). The lowest relaxation is observed for PEG-4Ox (Fig. S11B). Since stress relaxation is linked to bond exchange dynamics, it becomes evident that oxime gels have extremely low or negligible hydrolysis, and that among the two oximes, PEG-4Ox is the most stable due to the electronic differences already discussed in the context of exchange kinetics. This materials-level characterization is thus in line with the above assumption made for our kinetic model (Fig. 3). Additionally, for each gel, the relaxation time follows the order: pH 6 <pH 7 with catalyst <pH 7 without catalyst <pH 8. This result demonstrates that the trend observed for the kinetics of exchange gelation also applies to the kinetics of hydrolysis that determines the stress relaxation, which is coherent with the established theoretical model that correlates the molecular parameters (i.e., kinetics of dissociation, here hydrolysis) with the mechanics of the gel (i.e., relaxation time)^39^.

All the gels at pH 6 and pH 7 with catalyst are fully relaxed in less than 24 h, and thus the stress relaxation data can be quantified according to the Maxwell model with a single exponential decay to extract the characteristic relaxation time, τ (Fig. 4G), which takes the form:

2 $$G\left(t\right)={G}_{0}{e}^{-(t/\tau )}$$

Hydrazone and oxime gels differ in the characteristic relaxation time by ca. two orders of magnitude. For the oxime gels at pH 7 and pH 8, the slow relaxation does not allow a reliable extraction of τ, therefore, we chose an alternative assessment by determining the fractional decay compared to the initial modulus at *t* = 24 h. The obtained values are between 0.7 and 0.9 (Table S3), highlighting the similarity between the gels obtained directly or via exchange.

### Topological reconfiguration of hydrogels post-fabrication

**Fig. 5:**
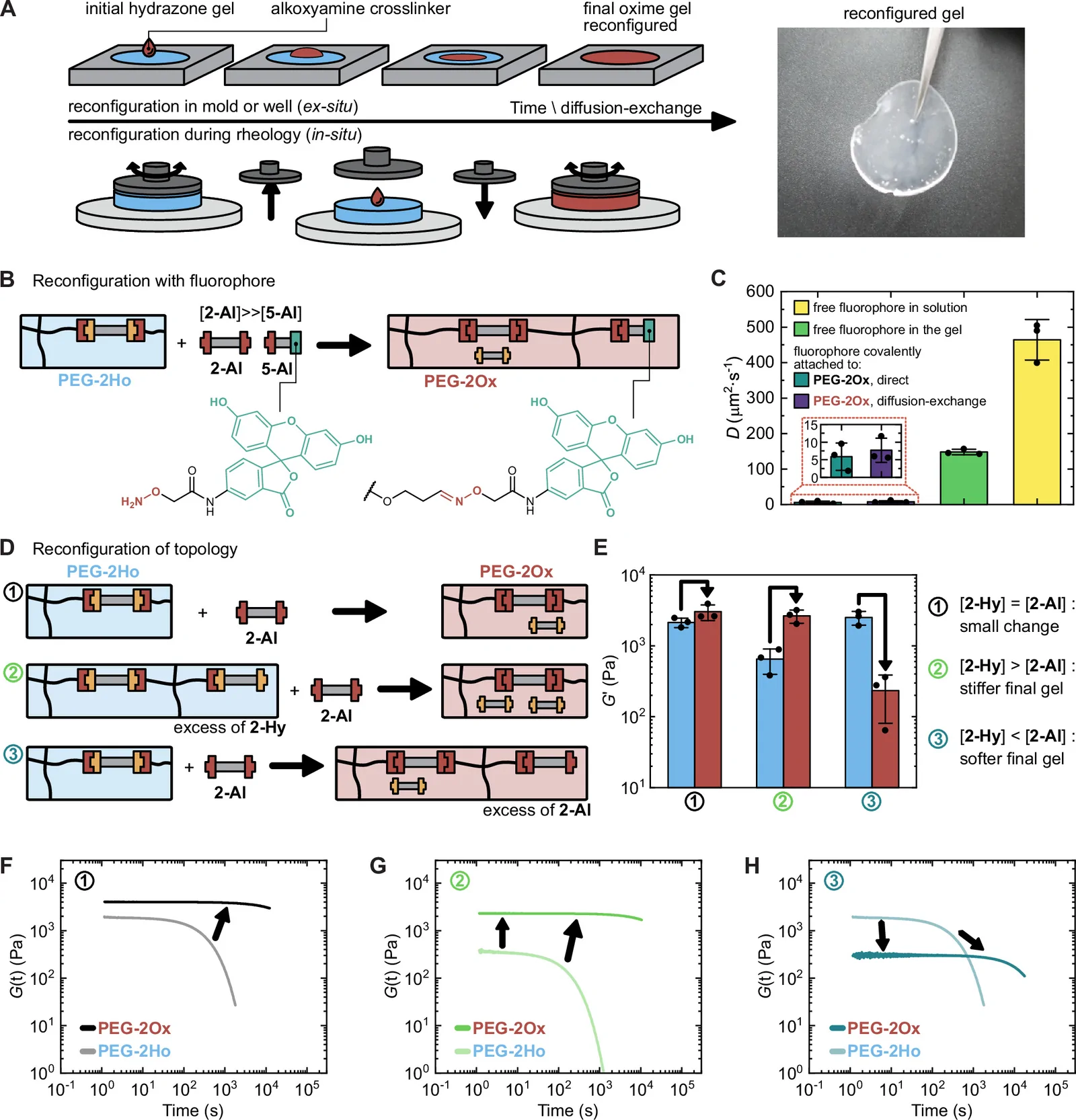
Reconfiguration of mechanical and topological properties of hydrogels post-fabrication. **A** Scheme of methodologies for the reconfigurations. The image shows a representative reconfigured oxime gel PEG-2Ox, with 1/1 exchange. **B** Dye labeling during exchange to measure FRAP. **C** Diffusion coefficients (*D*) from FRAP for 5-Al attached to PEG-2Ox gels either by direct gelation or during diffusion-exchange from PEG-2Ho versus controls, free dye (5(6)-Carboxyfluorescein) in PBS or in PEG-2Ox. *n* = 3 technical replicates in different regions of the same sample. Bars show mean values. Error bars represent standard deviations. The inset shows a magnified view of the near-zero values. Diffusion coefficients of fluorophores attached to the network are near zero, unlike those for the free fluorophore in solution or in the gel. **D** Reconfiguration of crosslinking segments and topology. **E** *G*′ of the initial PEG-2Ho gels and PEG-2Ox gels after reconfiguration, with related conditions regarding the use of 2-Hy and 2-Al, as indicated. Tabulated values and stoichiometric details in Table S4. *n* = 3. Bars show mean values. Error bars represent standard deviations. **F**–**H** Comparative stress relaxations of the initial hydrazone gels versus the reconfigured oxime gel in three representative topological reconfigurations (same labels as in **E**).

One of the emerging key advantages of the efficient exchange processes is that it opens doors for the seamless and destruction-free reconfiguration of hydrogel network topologies and functionalities through the diffusion of reactive alkoxyamine species through the gel and concomitant exchange reaction (*i.e*., hereafter referred to as diffusion-exchange). We implemented this method for both gels in molds ex-situ and during rheology in-situ by simply pipetting alkoxyamine solutions onto pre-formed hydrazone gels (Fig. 5A).

To confirm the diffusion-exchange within the gel, we first performed an ex-situ reconfiguration of a hydrazone gel (PEG-2Ho) using a mix of alkoxyamine crosslinker 2-Al and a minor fraction of dye-functionalized alkoxyamine 5-Al (Fig. 5B). Fluorescence recovery after photobleaching (FRAP) analysis in a confocal laser scanning microscope provides a direct way to analyze the diffusivity of the dye (details in Fig. S12). FRAP measurements reveal a near-zero diffusion coefficient (*D*, Fig. 5C) for the dye after the diffusion-exchange, confirming its attachment to the network. *D* is on the same level as for a direct addition of 5-Al to a gelating PEG-Ald/2-Al system and is orders of magnitude lower than for the free dye in solution or when not attached to the network, with values in the range of ca. 100–500 µm²/s, in line with previous studies^40^.

We next devised a set of reconfiguration experiments to modulate the mechanical properties of the hydrogels in a controlled manner (Fig. 5D). We first consider a full 1:1 exchange of hydrazine by alkoxyamine crosslinkers, before turning to topological reconfiguration achieved by tuning the equivalents of the hydrazine crosslinker used for the fabrication and alkoxyamine crosslinker added for the reconfiguration.

A complete 1:1 exchange using 2-Al shows a slightly higher *G*′ for the final oxime gel (Fig. 5E, number 1), which originates from the higher hydrolytic stability of the oxime linkages, as stated previously. We then address topological reconfiguration of the hydrogel networks by employing off-stoichiometric ratios of the crosslinkers. Conceptually, when hydrazine is present in excess during gel fabrication, dangling chain ends lead to a softened initial hydrazone network. In contrast, when excess alkoxyamine is introduced during reconfiguration, dangling reconfigured chain ends lead to a softened reconfigured oxime gel (schematically summarized in Fig. 5D). To showcase this behavior, an exchange from a stoichiometric ratio of 2-Hy to aldehyde groups of PEG of 1.15 to a ratio of 2-Al to aldehyde groups of PEG of 1 yields a transition from soft to stiff due to removal of dangling chains. On the contrary, an exchange from a stoichiometric ratio of 2-Hy to aldehyde groups of PEG of 1 to a ratio of 2-Al to aldehyde groups of PEG of 1.45 yields a transition from stiff to soft due to introduction of dangling chains (Fig. 5E, respectively numbers 2 and 3). Figure S13 shows full rheological in situ data with transient softening due to intermediate formation of copious dangling ends that are later crosslinked. Increasing the stoichiometric ratios to 1:2 or 2:1 causes loss of percolation during reconfiguration or a sol state before the reconfiguration (Fig. S14).

Next to the topology, stress relaxation properties are also altered (Fig. 5F–H). In all cases, the initial hydrazone gel exhibits faster stress relaxation, whereas the reconfigured oxime gel exhibits low stress relaxation typical of the gels bearing oxime crosslinks, as previously discussed (Fig. 4). The different approaches regarding stoichiometric and off-stoichiometric exchanges demonstrate that gel stiffness and viscoelastic dissipation can be modulated with a certain level of orthogonality depending on the starting and end components, providing a versatile approach to reprogramming material properties on demand.

### Reconfiguration of functional and viscoelastic properties

Finally, we exploit post-fabrication reconfiguration to achieve more advanced reprogramming of the gel properties using specifically engineered alkoxyamine crosslinkers bearing intrinsic functionalities. Among the wide range of functional motifs in polymer materials, disulfides and coordination complexes represent versatile moieties imparting responsiveness orthogonal to amine-imine systems^41^. For the disulfide component, we prepared a cystamine-based alkoxyamine crosslinker (6-Al), whereas we based the coordination complex on a Fe^3+^-catechol unit (7-Al).

**Fig. 6:**
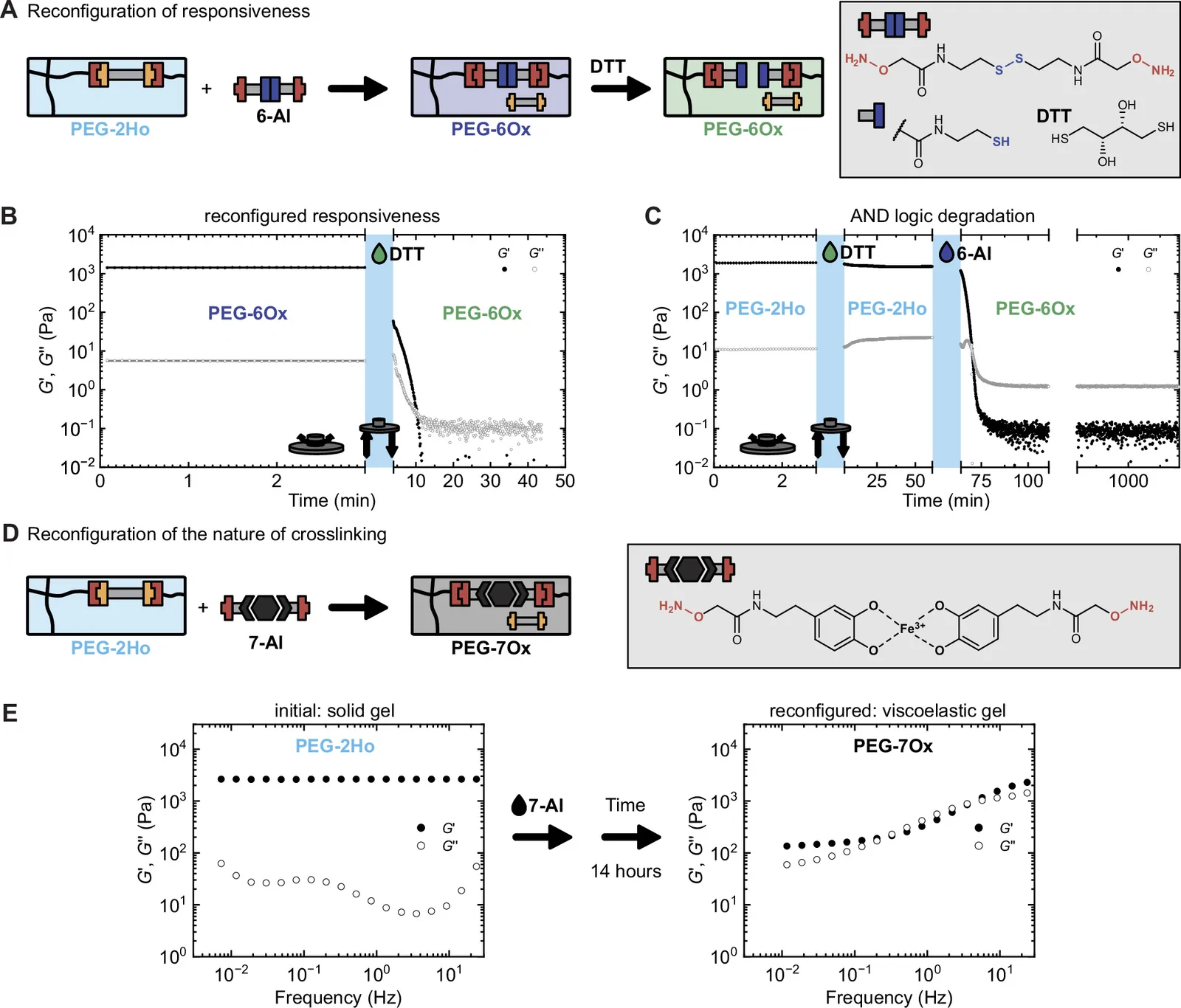
Reconfiguration of functional and viscoelastic properties of hydrazone hydrogels post-fabrication. **A** Reconfiguration scheme for imparting redox responsiveness into hydrazone gels using a disulfide-bearing alkoxyamine crosslinker. **B** Redox response as seen by rheology for an ex-situ reconfigured PEG-6Ox gel. **C** Time sweep of in situ reconfiguration by addition of DTT followed by alkoxyamine 6-Al. The gel-to-sol transition, upon addition of DTT, is selectively achieved after the exchange reaction. **D** Reconfiguration to incorporate metal-ligand-bearing Fe^3+^-catechol alkoxyamine crosslinkers, 7-Al. **E** Frequency sweeps of the initial hydrazone gel PEG-2Ho and of the reconfigured oxime gel PEG-7Ox reveal the transition from low dissipation to high dissipation.

We first discuss redox responsiveness enabled by disulfide-based alkoxyamine crosslinkers (Fig. 6A). To this end, we first prepared a hydrazone gel (PEG-2Ho), reconfigured it outside of the rheometer, and then placed it into the rheometer to probe switchable mechanical behavior by monitoring *G*′ and *G*″. Indeed, upon addition of dithiothreitol (DTT) as reducing agent, a gel-to-sol transition occurs as seen by the strong drop in *G*′ and the *G*′/*G*″ crossover (Fig. 6B). Conversely, addition of DTT to the pristine hydrazone gel causes only negligible changes of the moduli, and the gel maintains its structural integrity (Fig. 6C). However, the subsequent addition of 6-Al induces a gel-to-sol transition due to a combination of reduction and hydrazone-to-oxime reconfiguration, which together eliminate the crosslinks.

We next examine how to impart further non-covalent viscoelasticity by a coordination complex-bearing crosslinker. As a representative example, we selected a Fe^3+^-catechol system to impart dynamic crosslinks into the reconfigured hydrogels. This motif provides viscoelastic behavior that is orthogonal to amine–imine chemistry and is therefore well suited for post-fabrication functionalization. Importantly, this system requires avoiding PBS as it can interfere with the complexation. Thus, we used deionized water with pH adjusted to around 7.5, a regime at which Fe^3+^ is coordinated by two or three molecules of catechol^42,43^. Similar to the other cases, the reconfiguration proceeds smoothly (Fig. 6D), and frequency sweeps before and after the reconfiguration reveal profound changes in the viscoelastic response. Introduction of the Fe^3+^-catechol crosslinker (Fig. 6E) results in dynamic relaxation behavior, characterized by a characteristic *G*′/*G*″ crossover at ca. 4-5 Hz. In contrast, the original hydrazone gel shows a nearly parallel development of *G*′ and *G*″, with *G*′ being one order of magnitude higher than *G*″. Hence, in this frequency regime, no relaxation events take place. Note that this is in line with stress relaxation experiments (Fig. 4F) that probe much longer relaxation times more effectively. Collectively, these two examples underscore how our efficient reconfiguration chemistry can be used to impart new modes of responsiveness and dynamic properties to the materials via a very simple exchange process.

## Discussion

Here, we have established a general paradigm for post-fabrication reconfiguration of molecular systems and polymer hydrogels using thermodynamically imbalanced dynamic covalent chemistry. Unlike equilibrium-based bond exchange with one fixed type of dynamic motif, our strategy exploits differences in hydrolytic stability to drive directional, near-quantitative bond exchange, enabling functional crosslink replacement in multicomponent networks and providing a chemical handle to reprogram material properties.

At the molecular level, our study provides a detailed and quantitative understanding of hydrazone-to-oxime exchange in aqueous environments. By combining systematic kinetic experiments with numerical modeling, we disentangle the competing direct and hydrolytic pathways and identify – unlike in organic solvents –hydrolysis as the dominant mechanism governing exchange under relevant conditions. Importantly, the reaction-order mismatch inherent to the hydrolytic pathway gives rise to a non-trivial dependence of the apparent exchange kinetics on reactant concentration. The resulting kinetic framework, supported by simulations and analytical approximations, offers predictive insights into how molecular parameters control the rate and extent of exchange. This represents a highly comprehensive mechanistic and kinetic description of such an exchange reaction in water.

Building on this molecular understanding, we translate the exchange concept to the materials level and demonstrate controlled reconfiguration of hydrogels. Efficient exchange enables predictable tuning of gelation kinetics, stress relaxation, and elastic moduli while preserving macroscopic integrity. By exploiting diffusion–exchange, pre-formed hydrogels can be chemically reprogrammed in situ, allowing modulation of network topology and mechanical states without intermediate material destruction. Moreover, incorporation of functional alkoxyamine crosslinkers enables reprogramming of responsiveness and viscoelastic behavior, illustrating how molecular exchange can be leveraged to introduce new material functions without redesigning the original network.

Beyond the specific hydrazone–oxime system studied here, the underlying design principle – using thermodynamic bias to drive directional bond exchange – should be broadly applicable to other dynamic covalent chemistries and material platforms. Post-fabrication chemical reconfiguration opens a pathway toward soft materials that can adapt their structure and function over time, enabling sequential, context-dependent, or environment-triggered performance from a single initial material. At a conceptual level, the ability to transform one network into multiple functional states echoes emerging ideas of multipotency and pluripotency in synthetic materials^44^. We anticipate that the framework established here will stimulate further exploration of reconfigurable materials and strengthen the link between molecular reaction design and emergent bulk properties.

## Methods

### Materials

All chemicals were purchased from different commercial suppliers and used as received. *5(6)*-Carboxyfluorescein (Fluka), adipic dihydrazide 2-Hy (TCI Chemicals, 99%), O,O′-1,3-Propanediylbishydroxylamine dihydrochloride 2-Al (Sigma Aldrich, 98%), O-(carboxymethyl)hydroxylamine hemihydrochloride 1-Al (Sigma Aldrich, 98%), (1H-Benzo[d]imidazol-2-yl)methanamine catalyst (BLD pharm, 97%), 3-Methoxypropionaldehyde 1-Ald (BLD pharm, 90%). 4-Arm PEG-Aldehyde 10 kDa PEG-Ald (Creative PEGWorks), HPLC-grade acetonitrile (VWR), phosphate buffered saline (Thermo Fisher Scientific, 100 mM or 10 mM PBS) were used as received. Deionized water was used in Milli-Q grade except for synthesis. Teflon molds were fabricated in-house using a CNC milling station BZT PF 750-P. TLC was performed on Merck TLC silica gel 60 F254 TLC plates with a fluorescent indicator. Purification by automatic chromatography was performed on a BUCHI C810 Flash Basic Chromatography System, equipped with a UV-Vis detector.

### NMR Spectroscopy

^1^H, ^13^C and ^19^F NMR spectra were recorded on a 400 MHz Bruker Avance II 400 spectrometer (^13^C: 101 MHz; ^19^F: 376 MHz) or a 300 MHz Bruker Avance III HD 300 spectrometer (^13^C: 75 MHz; ^19^F: 282 MHz). Chemical shifts were reported in *δ* units. The multiplicity of the peaks was indicated as follows: s = singlet, d = doublet, t = triplet, q = quartet, m = multiplet. Deuterated solvents were purchased from Deutero GmbH. The spectra were analyzed using the software MestReNova.

### UV-Vis spectroscopy

UV-Vis absorption spectra were measured using an Ocean QE Spectrometer equipped with an Ocean CUV-QPOD temperature-controlled cuvette holder. Quartz cuvettes with *l* = 1 mm were used for all the experiments.

### Electrospray ionization high-resolution mass spectrometry (ESI HRMS)

ESI HRMS measurements were executed on an Agilent 6545 QTOF-HRAM-MS device.

### Confocal laser scanning microscopy (CLSM)

Confocal laser scanning microscopy (CLSM) was performed on a Leica Stellaris 5 device. Recorded images were analyzed with the software ImageJ.

### High-performance liquid chromatography (HPLC)

A Thermo Fisher Scientific Dionex Ultimate 3000 HPLC system was used, and chromatograms were processed using the Chromeleon data system. The samples were detected at the wavelength of 280 nm, using a diode array detector, for integral analysis. Analytical HPLC was performed over a Nucleosil 100-5-C18, 4.6 × 250 mm reversed-phase column, using a gradient water/acetonitrile from 95:5 to 5:95 ratio (v/v), followed by isocratic 0.1% trifluoroacetic acid (TFA) in water/acetonitrile 95:5 ratio (v/v). Preparative HPLC was performed over a Nucleosil 100-5-C18, 21 × 250 mm reversed-phase column, using a gradient water/acetonitrile from 95:5 to 5:95 ratio (v/v).

### Kinetic simulations

Numerical treatment to solve sets of ODEs (ordinary differential equations) was performed using the software COPASI as an open-source tool (https://copasi.org/). Within the software interface, one biochemical compartment was defined in which all the adequate species and reactions (mass action, irreversible or reversible) were set with specified values of kinetic constants. Thus, all the differential equations were generated, and the time-course task was performed using the deterministic LSODA method. The numerical solutions gave the trends of the concentrations of the species against the reaction time. Datasets were exported, plotted, and fitted subsequently.

### Rheology

Rheological measurements were performed using an Anton Paar Modular Compact Rheometer MCR 302 equipped with a 25 mm plate-plate geometry, at 25 °C, in shear mode. All measurements were analyzed with the RheoCompass software. Silicone oil M 350 medium viscosity was placed around the sample during measurements for any experiment that required more than 30 min, to avoid significant water evaporation. Time sweeps were performed at 1 Hz at 1% amplitude. Frequency sweeps were performed at 1% amplitude. Stress relaxations were performed at 10% amplitude.

### Use of stock solutions

Hydrazine 2-Hy and alkoxyamines 1-Al, 2-Al, and synthesized 4-Al were prepared at 200 or 400 mM in 100 mM PBS. 1-Ald was prepared at 200 mM in DMSO (the correct amount of aliquot to use was determined considering the purity of 90% of the commercial compound). Triethylamine (TEA) was prepared at 200 mM in DMSO.

### Control of pH

pH measurements of the required solutions were performed using a METTLER TOLEDO benchtop pH meter equipped with a microelectrode. Adjustments of the pH values were performed using HCl 1 or 4 M, and NaOH 1 or 6 M. The pH of the buffers, buffers containing catalyst, and all the stock solutions of the crosslinkers and reagents was carefully adjusted to the desired pH of the final solution or gel.

### HPLC analysis of the molecular reactions in water

All the reactions (structures in Figure S1, details in Table S1) were performed in a total volume of 0.5 mL of 100 mM PBS (PBS with or without 10 mM catalyst), at room temperature, with equimolar components at the final concentration of 10 mM (unless indicated otherwise). Note that for bifunctional molecules (2-Hy and 2-Al), the final concentration (10 mM) refers to the single functional group. 1-Hy and 3-Al were always prepared at the final concentration (in 100 mM buffer, and adjusted to the desired pH), and the required reactant was added from stock solution. For the exchange reactions with 1-Ho, this was prepared by equilibrating 1-Ald and 1-Hy for 14 h in water at the exchange conditions, and the alkoxyamine 1-Al or 2-Al was added from stock solution. Once the reagents were combined in an HPLC vial, the mixture was quickly shaken, moved in the HPLC sample holder, and the reaction was monitored with chromatograms obtained upon automatic injections. The intrinsic time gap between the data points was determined by the required duration of the HPLC runs and column re-equilibration. Residual concentrations of 1-Hy and 3-Al were determined by integration and correlation to related calibration data.

### General protocol for the fabrication of the hydrogels

As representative fabrication of the hydrazone gel PEG-2Ho, 10 kDa 4-Arm PEG-Aldehyde (PEG-Ald, 11.8 mg) was dissolved in a total volume of ca. 120 µL of 100 mM PBS, obtaining the final concentration of ca. 9.8 wt% (39.2 mM end-groups). Then, the crosslinker 2-Hy (1 equivalent with respect to the end-groups) was added from a stock solution (400 mM). The mixture was quickly shaken, transferred onto the rheometer plate, and the top plate was moved to the measuring position (0.2 mm). The PEG-Ald solution and the 2-Hy stock solution were adjusted to the same desired pH before combining them. For specific experiments, the pH and the buffer concentration were varied, other crosslinkers or mixtures were used, or a catalyst was added (always 1 equivalent with respect to the end-groups), always making sure that the final PEG concentration remains at 9.8 wt%. The use of catalyst always involved the preparation of the buffer with dissolved catalyst at the final concentration.

Samples were typically fabricated with three volumes: 120, 200, or 600 µL, depending on the experiment. 120 µL samples were used for gelation in the rheometer and in small vials. 200 µL samples (i.e., 19.6 mg of polymer) were used for gelation in a 96-well plate for CLSM. The gel precursor solution was placed in the well plate with care to fill the lower surface completely. 600 µL samples (i.e., 58.8 mg of polymer) were used for gelation in Teflon molds to obtain discs with 25 mm diameter and 1 mm thickness. The gel precursor solution was placed in the mold with particular care to fill all the inner surface of the mold and to avoid air bubbles in the sample.

### Gelation experiments in situ: direct, exchange, competition

Gelations in situ were always performed in 100 mM PBS, whereas the use of catalyst and the pH values are reported in Table S3. The fabrication of the gels by direct formation of hydrazones or oximes was carried out according to the general protocol. For the fabrication of the gels by exchange, PEG-Ald was combined with an excess of 2-Hy (2 equivalents) added from stock solution. The mixture was shaken quickly and vigorously to avoid the formation of any small insoluble polymer particles. After ca. 3 h of equilibration, the alkoxyamine crosslinker (1 equivalent) was added to achieve gelation. For the fabrication of the gels by competition, PEG-Ald was combined with a mixture of 2-Hy crosslinker (1 equivalent) and alkoxyamine crosslinker (1 equivalent).

The rheological analysis was carried out with the top plate adjusted to a 0.2 mm gap. Time sweep measurements were stopped when *G*′ clearly reached the plateau (e.g., for the fast-gelating samples PEG-2Ho and PEG-2Ox, direct), or when the increase of *G*′ became negligible (e.g., after hours or days for the slow-gelating samples PEG-2Ox via exchange and all the PEG-4Ox). Stress relaxations were stopped after complete decay for the fast-relaxing gels, or after 24 h for the slow-relaxing gels (e.g., PEG-2Ox at pH 7 and pH 8).

### General protocol for the reconfiguration of the hydrogels

All the reconfigurations were done on hydrazone gels PEG-2Ho fabricated in a vial, well, or mold. In every experiment, the buffer of the stock solutions of the crosslinkers and additional reagents was the same as the one used to fabricate the initial hydrazone gel to reconfigure. For specific experiments, the buffer concentration was varied, different crosslinkers or mixtures were used, or a catalyst was present in the initial hydrazone gel (always 1 equivalent with respect to the end-groups).

The ex-situ reconfiguration of the gels fabricated in vials or well plates was performed by pipetting a drop of the required crosslinker (and eventual additional reagent) onto the pre-formed gel. These were properly sealed with a screw cap and/or parafilm, allowing the reconfiguration (ca. 5 days) to occur. The ex-situ reconfiguration of the gels fabricated in the mold was performed by applying 5 drops of the required crosslinker (and, if needed, additional reagent), properly distributed onto the gel surface. The mold containing the sample was covered with a plastic lid, and some deionized water was left around the mold to avoid drying up the sample. The total weight of the mold with the sample was taken before any experiment. During the reconfiguration (ca. 5 days), the mold was periodically left in an open atmosphere to balance the gain of weight, caused by the absorption of moisture, with the loss of weight caused by drying. Thus, removal and handling of the final disc-shaped gel, for further analysis, was done, with its weight nearly identical to the initial sample.

The in-situ reconfiguration of the gels fabricated in the mold was performed in the rheometer. After the gel was transferred onto the rheometer plate, with the top plate, a drop of the required crosslinker (and any additional reagent) was applied onto the gel. The measuring position of the top plate was adjusted to a 1.1–1.2 mm gap to allow proper contact with the wet surface of the gel, but without squeezing out the added liquid at the edge. After quickly adding silicone oil, the measurement could be started to monitor the occurring reconfiguration.

### FRAP experiments

All the gels for FRAP were fabricated in 100 mM PBS, directly in a 96-well plate, according to the general protocol. Fluorophores were added from stock solutions (10 mM in 100 mM PBS) to reach a final concentration of 0.05 mM. Crosslinkers were added from stock solution to reach equimolarity with PEG-Ald. Oxime gel with pre-linked fluorophore was made by adding 5-Al before the crosslinker, allowing immediate ligation of the dye. For the reconfiguration, a hydrazone gel PEG-2Ho was first fabricated according to the general procedure, and after a few hours of equilibration 2-Al and 5-Al were added in this order. The final pH was always ca. 7. All the samples were thus analyzed with CLSM.

The FRAP experiments were performed using the point-bleaching method. First, a few pre-bleaching images were recorded, after which the bleaching was carried out for 3 s at 100% laser intensity, at a single pixel, using the characteristic excitation peak of fluorescein (*λ* = 488 nm). Subsequently, post-bleaching images were recorded over approximately 14 s, at maximum frame rates. These were analyzed by taking cross-sectional line profiles crossing the bleached center, giving different Gaussian-shaped curves of the fluorescence intensity for all the images at different time points. All these post-bleaching curves were fitted to a Gaussian function, giving a squared standard deviation (s.d.) of the Gaussian distribution, *σ*^2^. The diffusion coefficients (*D*) were then extracted by plotting all the obtained *σ*^2^ against *t*, and fitting the data linearly^45^:

3 $${\sigma }^{2}=2{Dt}$$

Where 2*D* is the slope of the linear function.

### Topological reconfigurations

All the initial hydrazone gels PEG-2Ho were fabricated in vials or in molds according to the general protocol, in 100 mM PBS with catalyst. The stoichiometry of the used crosslinkers (for fabrication and reconfiguration) is reported in Table S4. All the reconfigurations were done according to the general protocol. Hydrazine and alkoxyamine crosslinkers were added from stock solution (400 mM). All the experiments were performed using the buffer with catalyst and crosslinker stock solutions adjusted to pH ca. 7.

### Functional reconfiguration with 6-Al and DTT addition

The initial hydrazone gel PEG-2Ho was fabricated according to the general protocol, in 100 mM PBS with catalyst. For the ex-situ in-mold reconfiguration followed by DTT addition, 6-Al (1 equivalent) was added from stock solution (350 mM, adjusted to the final pH), and after 5 days the obtained gel was transferred to the rheometer. DTT (2.5 equivalents) was added from stock solution (1 M, adjusted to final pH), applying the general in-situ reconfiguration procedure. For the in situ reconfiguration with inverted order of addition of 6-Al and DTT (same equivalents and stock solutions), the fabricated hydrazone gel was immediately transferred into the rheometer; DTT was added from stock solution, followed by a brief time sweep analysis and addition of 6-Al from stock solution. All the experiments were performed using the buffer with catalyst, crosslinker stock solutions, and DTT stock solution adjusted to pH ca. 7.

### Functional reconfiguration with 7-Al

The initial hydrazone gel PEG-2Ho was fabricated using a solution of deionized water, according to the general protocol. The pristine deionized water and the 2-Hy crosslinker stock solution (freshly prepared in deionized water) were carefully adjusted to pH ca. 7.5. 7-Al was added (1 equivalent) from stock solution (100 mM). The complex 7-Al was prepared by combining the catechol ligand (see synthesis of compound 8 in the Supplementary Information) and FeCl_3_ (2:1 equivalents) to obtain an acidic solution (yellow-orange) of the complex at the final concentration of 100 mM (in deionized water), after which the pH was adjusted to ca. 7.5, obtaining a black solution.

## Supplementary information

Supplementary Information

Transparent Peer Review file

## Source data

Source Data

## Data Availability

The data supporting the findings of this study are available within the Article and its Supplementary Information. Raw data generated in this study, including rheological datasets, HPLC chromatograms, NMR and mass spectrometry spectra, confocal microscopy images, and kinetic simulation files, have been deposited in the Zenodo database and are available under accession code [https://doi.org/10.5281/zenodo.22008371] (ref. ^46^). All data are available from the corresponding author upon request. Source data are provided with this paper.
