# Supplementary material for: Post-fabrication reconfiguration of hydrogels through thermodynamic asymmetry in dynamic chemistry
Source: Nat Commun. 2026 Oct 7;17:10574. doi: 10.1038/s41467-026-78292-4 (PMC13645739; doi:10.1038/s41467-026-78292-4)
Supplement: Supplementary file 1 [file 41467_2026_78292_MOESM1_ESM.pdf]

Supplementary Information

Post-fabrication Reconfiguration of Hydrogels through Thermodynamic Asymmetry in Dynamic Chemistry

Davide Campagna[1,*], Diego Ciardi[1], Weixiang Chen[1], Andreas Walther[1,*]

Affiliations

[1]Life-Like Materials and Systems, University of Mainz, Duesbergweg 10-14, 55128 Mainz, Germany.

*Corresponding author: davide.campagna@uni-mainz.de; andreas.walther@uni-mainz.de

Table of Contents

Synthetic procedures 2

General procedure of the coupling with HBTU 2

General procedure of the Boc deprotection with TFA 2

Synthesis of 1-Hy and 3-Al 2

Synthesis of the crosslinker 4-Al 3

Synthesis of the dye 5-Al 4

Synthesis of the crosslinker 6-Al 4

Synthesis of the ligand 8 5

Supplementary methods and protocols 6

HPLC analysis of the molecular reactions in DMSO 6

Fitting to the second-order irreversible kinetics 6

Formation of the hydrazone monitored by UV-Vis spectroscopy 6

Fitting to second-order reversible kinetics for the hydrazone formation 7

Analytical solution for the equilibrium aldehyde-hydrazone 7

Supplementary Note 1. Kinetic simulations and mathematical model 8

Supplementary Tables 10

Supplementary Figures 12

Characterization spectra 23

Supplementary References 42

Synthetic procedures

General procedure of the coupling with HBTU

The reactions were performed in DMF with the carboxylic acid as limiting reactant at the final concentration of 0.2 M. Typically, the carboxylic acid and HBTU (1.1 equivalents) were dissolved in dry DMF, and a solution of L-tyrosinamide (1.1 equivalents) in DMF was added. Then, DIPEA (2 equivalents) was added, and the mixture was stirred overnight at room temperature. The volatiles were removed under reduced pressure at 50 °C, the obtained residue redissolved in MeCN, acidified with a few drops of HCl solution (4 M) to form DIPEA HCl, and purified by preparative HPLC.

General procedure of the Boc deprotection with TFA

The reactions were performed in a mixture of TFA/DCM in ca. 1:2 (v/v) ratio, with the substrate at the final concentration of ca. 0.25 M. Typically, the substrate (1 equivalent) was first dissolved in dry DCM, TFA (ca. 25 equivalents) was added slowly, and the mixture was stirred at room temperature for 3 hours. Then, the mixture was diluted with DCM (ca. 10 times the volume of the reaction mixture) and quenched with MeOH (ca. equal volume to reaction mixture) that acts by providing a protic environment that stabilizes the TFA. The solvent was removed under reduced pressure at 35 °C, the obtained residue was redissolved in MeOH, and the solvent was removed. This step was repeated 3 times. The final residue was dried *in vacuo*, after which it was dissolved in deionized water and freeze-dried, affording the deprotected product as trifluoroacetate salt. Maleic acid was used as internal standard in ^{1}H NMR to verify an adequate purity with values of (100 ± 5) % (the error takes into account the integration in the NMR spectrum analysis). Precise amounts of internal standard and product were dissolved in a total volume of 0.5 mL of D_2O, the integral of the vinyl signal of maleic acid was normalized and compared to the integral of a diagnostic signal of the product, allowing to calculate its final purity.

Synthesis of 1-Hy and 3-Al

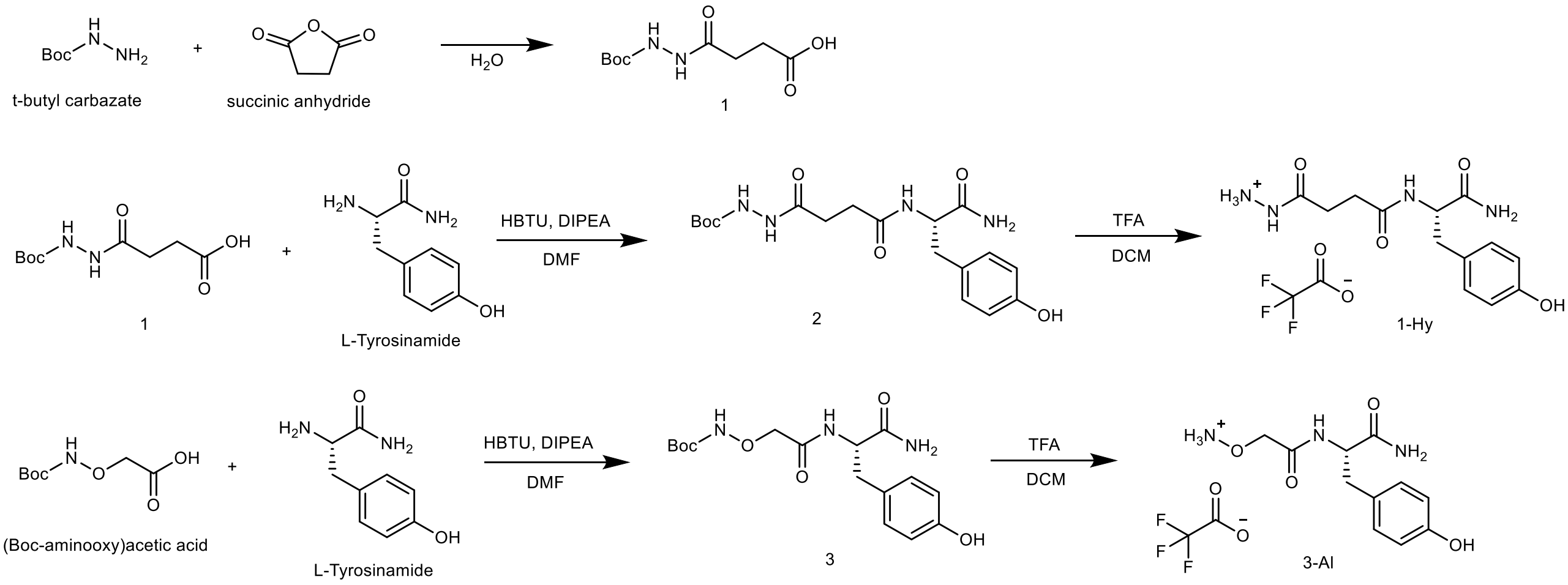

The synthesis of 1-Hy required the formation of the precursor **1**. To make this, *t*-butyl carbazate (2.64 g, 20 mmol, 1 equivalent) and succinic anhydride (2.00 g, 20 mmol, 1 equivalent) were dissolved in deionized water (40 mL), and the mixture was stirred overnight at room temperature. The solution was then directly freeze-dried and the solid product was afforded quantitatively without further purification. **^{1}H NMR (400 MHz, DMSO-d_6):** δ = 11.74 (s, 1H, -COO*H*), 9.54 (s, 1H, -N*H*), 8.71 (s, 1H, -N*H*), 2.47 – 2.24 (m, 4H), 1.40 (s, 9H). **^{13}C NMR (75 MHz, DMSO-d_6):** δ = 174.01, 171.20, 155.70, 79.45, 31.77, 29.19, 28.52.

Compound **2** was synthesized according to the general coupling procedure with HBTU, starting with reactant **1** (232 mg, 1 mmol, 1 equivalent). L-tyrosinamide (198 mg, 1.1 mmol, 1.1 equivalents), HBTU (417 mg, 1.1 mmol, 1.1 equivalents), and DIPEA (348 µL, 2 mmol, 2 equivalents). Product **2** was obtained after preparative HPLC, as white solid, in 22% yield. **^{1}H NMR (400 MHz, DMSO-d_6):** δ = 9.54 (s, 1H, -NHN*H*), 9.17 (s, 1H,

-O*H*), 8.69 (s, 1H, -NHN*H*), 8.03 (d, 1H, -N*H*), 7.34 (s, 1H, -NH_2), 7.02 (s, 1H, -NH_2), 7.02 (d, 2H), 6.64 (d, 2H), 4.34 – 4.24 (m, 1H), 2.90 (dd, 1H), 2.61 (dd, 1H), 2.38 – 2.18 (m, 4H), 1.40 (s, 9H). **^{13}C NMR (75 MHz, DMSO-*d*$_6$):** δ = 173.89, 171.73, 171.36, 156.17, 155.71, 130.46, 128.76, 115.31, 79.49, 54.79, 37.17, 30.83, 29.11, 28.53.

The pure compound **2** (71 mg, 0.18 mmol, 1 equivalent) was deprotected with TFA (342 µL, 4.50 mmol, 25 equivalents) according to the general procedure, affording 1-Hy quantitatively. **^{1}H NMR (400 MHz, D_2O):** δ = 7.07 (d, 2H), 6.76 (d, 2H), 4.45 – 4.37 (m, 1H), 3.00 (dd, 1H), 2.79 (dd, 1H), 2.49 – 2.20 (m, 4H). **^{13}C NMR (101 MHz, D_2O):** δ = 176.17, 174.54, 173.48, 164.93, 154.35, 130.47, 128.45, 117.78, 115.39, 54.89, 36.15, 30.29, 28.69. **^{19}F NMR (376 MHz, D_2O):** δ = -76.75.

The compound **3** was synthesized according to the general coupling procedure with HBTU, starting with the reactant (Boc-aminooxy)acetic acid (96 mg, 0.5 mmol, 1 equivalent). L-tyrosinamide (99 mg, 0.55 mmol, 1.1 equivalents), HBTU (209 mg, 0.55 mmol, 1.1 equivalents), and DIPEA (174 µL, 1 mmol, 2 equivalents). The product **3** was obtained after preparative HPLC, as white solid, in 83% yield. **^{1}H NMR (400 MHz, DMSO-*d*$_6$):** δ = 10.25 (s, 1H, -N*H*), 9.18 (s, 1H, -O*H*), 8.01 (d, 1H, -N*H*), 7.45 (s, 1H, -NH_2), 7.08 (s, 1H, -NH_2), 7.02 (d, 2H), 6.63 (d, 2H), 4.47 – 4.37 (m, 1H), 4.18 – 4.05 (m, 2H), 2.91 (dd, 1H), 2.68 (dd, J = 13.8, 9.1 Hz, 1H), 1.43 (s, 9H). **^{13}C NMR (75 MHz, DMSO-*d*$_6$):** δ = 173.14, 168.04, 157.26, 156.24, 130.51, 128.23, 115.33, 81.03, 74.97, 54.16, 37.48, 28.44.

The pure compound **3** (141 mg, 0.4 mmol, 1 equivalent) was deprotected with TFA (760 µL, 10 mmol, 25 equivalents) according to the general procedure, affording 3-Al quantitatively. **^{1}H NMR (400 MHz, D_2O):** δ = 7.09 (d, 2H), 6.76 (d, 2H), 4.56 – 4.48 (m, 1H), 4.16 – 4.02 (m, 2H), 3.05 (dd, 1H), 2.85 (dd, 1H). **^{13}C NMR (101 MHz, D_2O):** δ = 175.62, 170.88, 163.13, 154.46, 130.52, 128.19, 117.33, 115.45, 72.66, 54.44, 36.19. **^{19}F NMR (376 MHz, D_2O):** δ = -76.78.

Note. Depending on the quality of the batches, purification by preparative HPLC was additionally required. In this case, the determination of the final purity by ^{1}H NMR, using maleic acid as internal standard, revealed mixtures of neutral and TFA salt forming in the same isolated batch. The value of purity obtained assuming pure TFA salt form allowed to derive the ratio between neutral and salt form. Thus, the correct molecular weight of the specific batch could be defined and used for the preparation of solutions at certain concentrations used in the subsequent kinetic studies.

Synthesis of the crosslinker 4-Al

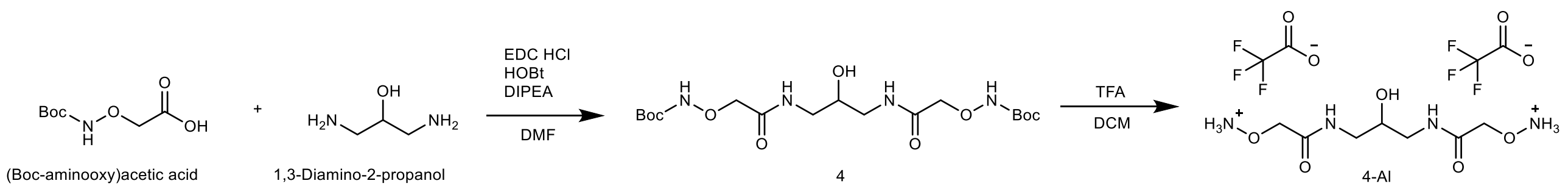

(Boc-aminooxy)acetic acid (956 mg, 5 mmol, 2 equivalents) and HOBt (777 mg, 5.75 mmol, 2.3 equivalents) were dissolved in dry DMF (10 mL), followed by the addition of EDC HCl (1.100 g, 5.75 mmol, 2.3 equivalents). The mixture was stirred at room temperature for 30 minutes, after which a solution of 1,3-diamino-2-propanol (225 mg, 2.5 mmol, 1 equivalent) and DIPEA (2.18 mL, 12.5 mmol, 5 equivalents) in dry DMF (2 mL) was added, and the full mixture was stirred overnight at room temperature. After adding water (ca. 2 mL) to quench the reaction, all the volatiles were removed under reduced pressure at 50 °C, and the residue was redissolved in EtOAc (100 mL) and washed with $NaHCO_3$ saturated solution (50 mL). The aqueous layer was extracted 2 more times with EtOAc (2x50 mL). The organic layers were collected, dried over $MgSO_4$, and the volatiles were removed under reduced pressure with gentle heating. The crude dry product was purified by automatic chromatography on silica gel (eluted with EtOAc/MeOH solvent system), affording the pure product **4**, as white solid, in 76% yield. **^{1}H NMR (300 MHz, DMSO-*d*$_6$):** δ = 10.26 (s, 2H, -N*H*), 7.95 (s, 2H, -N*H*), 5.05 (d, 1H, -O*H*), 4.16 (s, 4H), 3.67 – 3.53 (m, 1H), 3.26 – 3.00 (m, 4H), 1.42 (s, 18H). **^{13}C NMR (75 MHz, DMSO-*d*$_6$):** δ = 168.58, 157.17, 81.02, 75.18, 68.51, 42.93, 28.41.

The pure compound **4** (256 mg, 0.59 mmol, 1 equivalent) was deprotected with TFA (1.122 mL, 14.66 mmol, 25 equivalents) according to the general procedure, affording 4-Al quantitatively. **^{1}H NMR (300 MHz, DMSO-*d*$_6$):** δ = 8.17 (t, 2H, -N*H*), 4.39 (s, 4H), 3.69 – 3.56 (m, 1H), 3.27 – 3.00 (m, 4H). **^{13}C NMR (75 MHz, DMSO-*d*$_6$):** δ = 168.17, 159.10, 119.05, 72.45, 68.37, 42.90. **^{19}F NMR (376 MHz, D_2O):** δ = -76.82. **^{19}F NMR (282 MHz, DMSO-*d*$_6$):** δ = -74.24.

Synthesis of the dye 5-Al

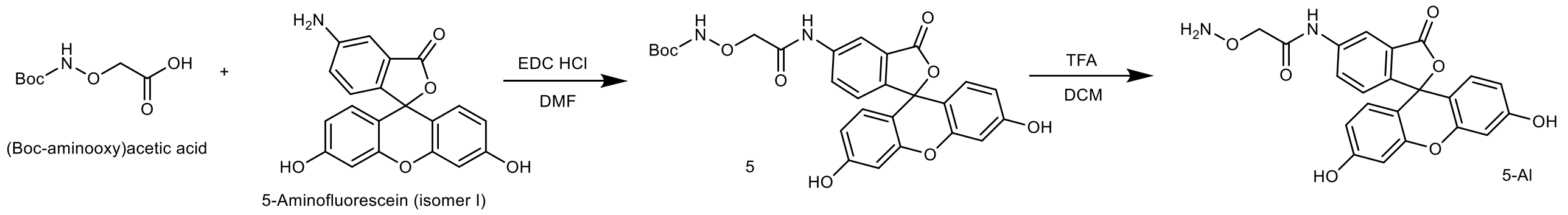

5-Aminofluorescein (50 mg, 0.144 mmol, 1 equivalent) and (Boc-aminooxy)acetic acid (112 mg, 0.576 mmol, 4 equivalents) were dissolved in dry DMF (1 mL). To this, a suspension of EDC HCl (112 mg, 0.576 mmol, 4 equivalents) in dry DMF/DCM (1 mL) in a 1:1 (v/v) ratio was added, and the mixture was stirred overnight at room temperature in the dark. The volatiles were removed under reduced pressure at 50 °C, the residue redissolved in MeCN/deionized water in a 3:1 (v/v) ratio and purified by preparative HPLC. The isolated product was freeze-dried, affording **5**, as orange solid, in 66% yield. **^{1}H NMR (400 MHz, DMSO-*d*$_6$):** δ = 10.52 (s, 2H, -N*H*), 10.16 (s, 2H, -O*H*), 8.36 (s, 1H), 7.90 (d, J = 2.0 Hz, 1H), 7.26 (d, J = 8.3 Hz, 1H), 6.68 (s, 2H), 6.63 – 6.52 (m, 4H), 4.44 (s, 2H), 1.44 (s, 9H). **^{13}C NMR (101 MHz, DMSO-*d*$_6$):** δ = 169.01, 168.10, 159.96, 157.61, 152.37, 147.75, 140.33, 129.56, 127.48, 127.09, 125.06, 114.30, 113.07, 110.10, 102.68, 83.62, 81.30, 75.56, 28.42. **ESI$^+$ HRMS:** *m/z* = 521.1555 (MH$^+$).

The pure compound **5** (39 mg, 0.076 mmol, 1 equivalent) dissolved in DCM (290 µL) was deprotected by adding slowly TFA (290 µL, 3.8 mmol, 50 equivalents). The reaction was carried out and the deprotected compound isolated according to the general procedure. The residue was redissolved in an equal mixture of MeCN and water, and it was purified by preparative HPLC. The isolated product was freeze-dried, affording 5-Al in the neutral form, as light orange solid, in 39% yield. **^{1}H NMR (400 MHz, DMSO-*d*$_6$):** δ = 10.19 (s, 1H, -N*H*), 10.15 (s, 2H, -ON*H*$_2$), 8.40 (d, 1H), 7.99 (d, 1H), 7.23 (d, 1H), 6.68 (s, 2H), 6.63 – 6.52 (m, 4H), 6.51 (s, 2H, -O*H*), 4.21 (s, 2H). **^{13}C NMR (101 MHz, DMSO-*d*$_6$):** δ = 170.18, 169.10, 159.93, 152.36, 147.50, 140.66, 129.57, 127.35, 124.84, 114.50, 113.04, 110.17, 102.67, 83.55, 75.11. **^{19}F NMR (376 MHz, DMSO-*d*$_6$):** no significant peak. **ESI$^+$ HRMS:** *m/z* = 421.1024 (MH$^+$).

Synthesis of the crosslinker 6-Al

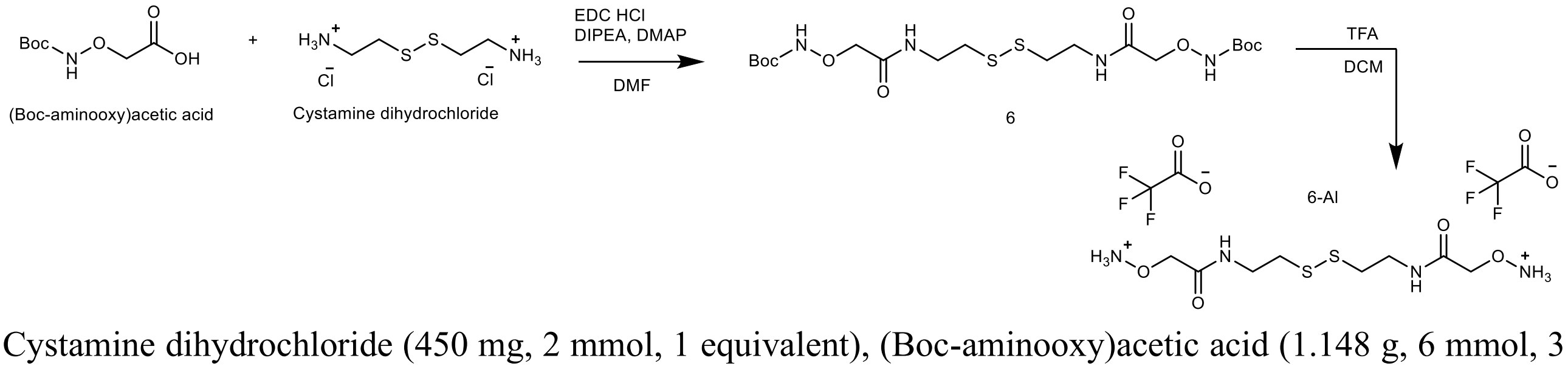

Cystamine dihydrochloride (450 mg, 2 mmol, 1 equivalent), (Boc-aminooxy)acetic acid (1.148 g, 6 mmol, 3 equivalents), DIPEA (696 µL, 4 mmol, 2 equivalents), and DMAP (32 mg, 0.26 mmol, 0.13 equivalents) were combined in dry DMF (10 mL). Then, EDC HCl (1.15 g, 6 mmol, 3 equivalents) was added, and the mixture was stirred overnight at room temperature. All the volatiles were removed under reduced pressure at 50 °C, the residue was redissolved in EtOAc (100 mL), and the turbid solution was washed with 50 mL of NH_4Cl saturated solution (50 mL) and 50 mL of NaCl saturated solution (50 mL). The organic layer was dried over $MgSO_4$ and the volatiles removed under reduced pressure with gentle heating. The crude dry product was purified by automatic chromatography on silica gel (eluted with cyclohexane/EtOAc solvent system), affording the pure product **6**, as white solid, in 63% yield. **^{1}H NMR (300 MHz, DMSO-*d*$_6$):** δ = 10.28 (s, 1H, -N*H*),

8.17 (t, 1H, -N*H*), 4.16 (s, 2H), 3.44 (dt, 2H), 2.81 (t, 2H), 1.42 (s, 9H). **^{13}C NMR (101 MHz, DMSO-d_6):** δ = 168.52, 157.28, 81.09, 75.14, 38.01, 37.40, 28.41.

The pure compound **6** (150 mg, 0.3 mmol, 1 equivalent) was deprotected with TFA (570 µL, 7.50 mmol, 25 equivalents) according to the general procedure, affording 6-Al quantitatively. **^{1}H NMR (400 MHz, D_2O):** δ = 4.52 (s, 2H), 3.50 (t, 2H), 2.79 (t, 2H). **^{13}C NMR (101 MHz, D_2O):** δ = 168.72, 163.41, 120.62, 71.70, 37.84, 36.23. **^{19}F NMR (376 MHz, D_2O):** δ = -76.76.

Synthesis of the ligand 8

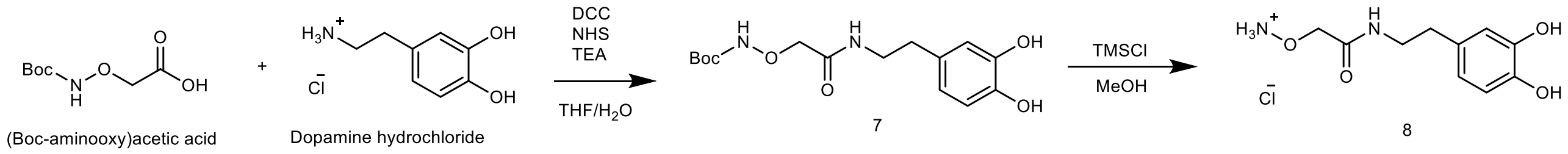

(Boc-aminooxy)acetic acid (765 mg, 4 mmol, 1 equivalent) and NHS (460 mg, 4 mmol, 1 equivalent) were dissolved in dry THF (10 mL), followed by the addition of DCC (825 mg, 4 mmol, 1 equivalent). The mixture was stirred at room temperature for few hours. The solution was filtered to remove the precipitated dicyclohexylurea (DCU) and the filtrate washed with a few mL of THF. To the combined THF mixture, a solution of dopamine hydrochloride (759 mg, 4 mmol, 1 equivalent) and TEA (558 µL, 4 mmol, 1 equivalent) in deionized water (3 mL) was added dropwise in an ice bath, observing the formation of a brownish color. The mixture was stirred overnight at room temperature, under inert atmosphere and in the dark. All the volatiles were removed under reduced pressure with gentle heating, the residue was redissolved in EtOAc and washed with a HCl solution (0.1 M). The organic layer was dried over $MgSO_4$ and the volatiles removed under reduced pressure with gentle heating. The crude product was purified by automatic chromatography on silica gel (eluted with EtOAc), affording the pure product **7**, as white solid, in 52% yield. **^{1}H NMR (300 MHz, DMSO-d_6):** δ = 10.28 (s, 1H, -N*H*), 8.76 (s, 1H, -O*H*), 8.67 (s, 1H, -O*H*), 8.03 (t, 1H, -N*H*), 6.68 – 6.55 (m, 2H), 6.45 (dd, 1H), 4.14 (s, 2H), 3.25 (dt, 2H), 2.56 (t, 2H), 1.42 (s, 9H). **^{13}C NMR (75 MHz, DMSO-d_6):** δ = 168.20, 157.32, 145.56, 144.06, 130.35, 119.62, 116.35, 115.96, 81.09, 75.23, 35.06, 28.39.

As TFA-based deprotection was avoided due to oxidative side reactions, TMSCl in MeOH was used to produce HCl *in-situ*. The pure compound **7** (326 mg, 1 mmol, 1 equivalent) was dissolved in MeOH (15 mL) and the solution was bubbled with N_2 stream for 20 minutes. Then, TMSCl (381 µL, 3 mmol, 3 equivalents) was added dropwise, and the mixture was stirred overnight at room temperature, carefully sealed under inert atmosphere and in the dark. The volatiles were removed under reduced pressure at 200 mbar and 35 °C. The residue was redissolved in MeOH and the solvent removed under reduced pressure. The residue was dried fully *in vacuo*, affording the chloride salt form of **8** as white crystalline solid, quantitatively. **^{1}H NMR (300 MHz, D_2O):** δ = 6.74 (d, 1H), 6.68 (d, 1H), 6.59 (dd, 1H), 4.42 (s, 2H), 3.34 (t, 2H), 2.60 (t, 2H). **^{13}C NMR (75 MHz, D_2O):** δ = 168.43, 143.79, 142.29, 131.76, 121.09, 116.56, 116.16, 71.66, 40.37, 33.58.

Supplementary methods and protocols

HPLC analysis of the molecular reactions in DMSO

The reactions in pure DMSO were performed in a total volume of 0.5 mL, at room temperature, with equimolar components at the final concentration of 10 mM. 3-Al was always prepared at final concentration (in DMSO, with or without catalyst). For the direct oxime formation and for the exchange reactions, a solution of 3-Al was prepared, and the required reactants were added from stock solution. For the reverse exchange reaction, a solution of 3-Ox was prepared at the final concentration by leaving combined equimolar 3-Al and 1-Ald to equilibrate for 3 days in presence of 1 equivalent of TEA, and 2-Hy was added from stock solution (100 mM in DMSO). The monitoring with the HPLC was performed as for the experiments in water.

Fitting to the second-order irreversible kinetics

Datasets obtained either experimentally (HPLC) for the direct oxime formations and exchange reactions, or by kinetic simulations (COPASI) of the exchange reactions, in the form of consumption of a reactant A over time, were fitted to a second-order irreversible kinetic model. The reaction equation of the direct oxime formation takes the form:

$$\mathrm{A} + \mathrm{B} \rightarrow \mathrm{C} \tag{1}$$

Whereas for the exchange reaction takes the form:

$$\mathrm{A} + \mathrm{B} \rightarrow \mathrm{C} + \mathrm{D} \tag{2}$$

In both cases, for initial A and B in equimolar ratio:

$$[\mathrm{A}]_0 = [\mathrm{B}]_0 \tag{3}$$

The differential equation can be written as:

$$-\frac{d[\mathrm{A}]}{dt} = k_{\mathrm{obs}}[\mathrm{A}]^2 \tag{4}$$

Which, after integration, gives the analytical solution:

$$\frac{1}{[\mathrm{A}]} = \frac{1}{[\mathrm{A}]_0} + k_{\mathrm{obs}}t \tag{5}$$

Whose form can be rearranged:

$$[\mathrm{A}] = \frac{1}{\left(\frac{1}{[\mathrm{A}]_0} + k_{\mathrm{obs}}t\right)} \tag{6}$$

Formation of the hydrazone monitored by UV-Vis spectroscopy

The hydrazone 2-Ho formation in water (first reaction in Figure S1) was monitored by UV-Vis spectroscopy to determine the related k_1, k_{-1}, and K_a, as reported.[1–3] The process was carried out in a cuvette in a total volume of 2 mL of 100 mM PBS/DMSO in a 200:1 (v/v) ratio, monitoring the peak of the hydrazone absorption band (λ = 240 nm). A solution of 1-Ald was prepared at the concentration of 1 mM in the correct solvent mixture, adjusted to pH ca. 7, and 1 equivalent of 2-Hy was added from stock solution (100 mM in 100 mM PBS). The cuvette with the final solution was quickly shaken and placed in the holder to start the time-course UV-Vis measurement. Data points were taken initially every 2 minutes, then every 5 minutes, and every 10 minutes. Derived concentrations of 2-Hy give the consumption trend to be used for the subsequent fitting. For the calibration, a batch of solutions of 1-Ald at different concentrations (0.1-0.3 mM in 100 mM PBS) were prepared by a dilution series starting from stock solution of 1-Ald. Each final solution contained 10 equivalents

of 2-Hy (with respect to 1-Ald), added from stock solution, assuming complete formation of 2-Ho. The pH of all the calibration solutions was adjusted to pH ca. 7.

Fitting to second-order reversible kinetics for the hydrazone formation

In a second-order reversible kinetics, the generic reaction equation takes the generic form:

$$\mathrm{A} + \mathrm{B} \rightleftharpoons \mathrm{C} \tag{7}$$

Assuming that generic Hy and Ald are A and B, respectively, and generic Ho is C, the Dirksen kinetic analysis[1] provides an analytical solution with the condition of having initial A and B in equimolar ratio:

$$[\mathrm{A}]_0 = [\mathrm{B}]_0 \tag{8}$$

The differential equation takes the form:

$$\frac{d[\mathrm{C}]}{dt} = k_1[\mathrm{A}]^2 - k_{-1}[\mathrm{C}] = k_1[\mathrm{A}]^2 - k_{-1}([\mathrm{A}]_0 - [\mathrm{A}]) \tag{9}$$

Integration gives:

$$[\mathrm{A}](t) = \frac{a_+([\mathrm{A}]_0 - a_-) - a_-([\mathrm{A}]_0 - a_+)\mathrm{e}^{-k_1(a_+ - a_-)t}}{([\mathrm{A}]_0 - a_-) - ([\mathrm{A}]_0 - a_+)\mathrm{e}^{-k_1(a_+ - a_-)t}} \tag{10}$$

Which, after fitting with the experimental data of 2-Hy consumption, gives the values of k_1, a_+, and a_-, where:

$$a_+ = \frac{-k_{-1} + \sqrt{k_{-1}^2 + 4k_1k_{-1}[\mathrm{A}]_0}}{2k_1} \tag{11}$$

$$a_- = \frac{-k_{-1} - \sqrt{k_{-1}^2 + 4k_1k_{-1}[\mathrm{A}]_0}}{2k_1} \tag{12}$$

And, focusing on the positive solution, a rearrangement gives:

$$k_{-1} = \frac{k_1a_+^2}{([\mathrm{A}]_0 - a_+)} \tag{13}$$

Having determined both k_1 and k_{-1}, K_a can be calculated as equal to k_1/k_{-1}.

Analytical solution for the equilibrium aldehyde-hydrazone

Considering the hydrazone formation as a second-order reversible reaction:

$$\mathrm{Hy} + \mathrm{Ald} \leftrightharpoons \mathrm{Ho} \tag{14}$$

If we assume initial equimolar Hy and Ald in equimolar ratio:

$$[\mathrm{Hy}]_0 = [\mathrm{Ald}]_0 \tag{15}$$

We can derive:

$$[\mathrm{Hy}]_{\mathrm{eq}} = [\mathrm{Ald}]_{\mathrm{eq}} = [\mathrm{Ald}]_0 - [\mathrm{Ho}]_{\mathrm{eq}} \tag{16}$$

$$K_{\mathrm{eq}} = \frac{[\mathrm{Ho}]_{\mathrm{eq}}}{[\mathrm{Hy}]_{\mathrm{eq}}[\mathrm{Ald}]_{\mathrm{eq}}} = \frac{[\mathrm{Ho}]_{\mathrm{eq}}}{\left([\mathrm{Ald}]_0 - [\mathrm{Ho}]_{\mathrm{eq}}\right)^2} \tag{17}$$

And the analytical solution for $[\mathrm{Ho}]_{\mathrm{eq}}$ takes the form:

$$[\mathrm{Ho}]_{\mathrm{eq}} = \frac{2K_{\mathrm{eq}}[\mathrm{Ald}]_0 + 1 \pm \sqrt{4K_{\mathrm{eq}}[\mathrm{Ald}]_0 + 1}}{2K_{\mathrm{eq}}} \quad (18)$$

Where the negative solution is physically meaningful.

Then, considering the hydrolysis of hydrazone (inverse process):

$$\mathrm{Ho} \leftrightharpoons \mathrm{Hy} + \mathrm{Ald} \quad (19)$$

The analytical solution for $[\mathrm{Ho}]_{\mathrm{eq}}$ is mathematically identical, and the only difference is that $[\mathrm{Ald}]_0$ is replaced by $[\mathrm{Ho}]_0$. Three datasets of $[\mathrm{Ho}]_{\mathrm{eq}}$ against $[\mathrm{Ho}]_0$ at different fixed K_a were generated using a Python script, and $[\mathrm{Ald}]_{\mathrm{eq}}$ was derived. The ratio $[\mathrm{Ald}]_{\mathrm{eq}}/[\mathrm{Ho}]_0$ can be plotted against $[\mathrm{Ho}]_0$, obtaining a sigmoidal curve.

Supplementary Note 1. Kinetic simulations and mathematical model

The reversible formation of the hydrazone is defined by the related set of ODEs. Given:

$$\mathrm{Hy} + \mathrm{Ald} \leftrightharpoons \mathrm{Ho} \quad (20)$$

The differential equations are:

$$\frac{d[\mathrm{Hy}]}{dt} = -k_1[\mathrm{Hy}][\mathrm{Ald}] + k_{-1}[\mathrm{Ho}] \quad (21)$$

$$\frac{d[\mathrm{Ald}]}{dt} = -k_1[\mathrm{Hy}][\mathrm{Ald}] + k_{-1}[\mathrm{Ho}] \quad (22)$$

$$\frac{d[\mathrm{Ho}]}{dt} = +k_1[\mathrm{Hy}][\mathrm{Ald}] - k_{-1}[\mathrm{Ho}] \quad (23)$$

Which were used to simulate the time-courses of hydrazone formation at 4 different values of initial equivalent concentration of Hy and Ald: 1, 2, 10, and 20 mM.

The exchange reaction is significantly more complex and its complete kinetic model is divided in three possible pathways that can coexist: dissociative without catalyst, dissociative with catalyst, and associative (Figure S3), where the dissociative is hydrolytic and the associative is the direct pathway. A detailed kinetic simulation was performed for the dissociative model without catalyst, for which two different cases are defined. The first one implies the irreversible oxime formation as approximation, thus lack of inverse reaction (*i.e.*, hydrolysis of oxime, with the kinetic constant k_{-2}):

$$\mathrm{Hy} + \mathrm{Ald} \leftrightharpoons \mathrm{Ho} \quad (24)$$

$$\mathrm{Al} + \mathrm{Ald} \rightarrow \mathrm{Ox} \quad (25)$$

For which the list of ODEs is:

$$\frac{d[\mathrm{Hy}]}{dt} = -k_1[\mathrm{Hy}][\mathrm{Ald}] + k_{-1}[\mathrm{Ho}] \quad (26)$$

$$\frac{d[\mathrm{Ald}]}{dt} = -k_1[\mathrm{Hy}][\mathrm{Ald}] + k_{-1}[\mathrm{Ho}] - k_2[\mathrm{Al}][\mathrm{Ald}] \quad (27)$$

$$\frac{d[\mathrm{Ho}]}{dt} = +k_1[\mathrm{Hy}][\mathrm{Ald}] - k_{-1}[\mathrm{Ho}] \quad (28)$$

$$\frac{d[\mathrm{Al}]}{dt} = -k_2[\mathrm{Al}][\mathrm{Ald}] \tag{29}$$

$$\frac{d[\mathrm{Ox}]}{dt} = +k_2[\mathrm{Al}][\mathrm{Ald}] \tag{30}$$

For the second case the oxime formation is also considered as reversible:

$$\mathrm{Hy} + \mathrm{Ald} \leftrightharpoons \mathrm{Ho} \tag{31}$$

$$\mathrm{Al} + \mathrm{Ald} \rightleftharpoons \mathrm{Ox} \tag{32}$$

Thus, giving the following list of ODEs:

$$\frac{d[\mathrm{Hy}]}{dt} = -k_1[\mathrm{Hy}][\mathrm{Ald}] + k_{-1}[\mathrm{Ho}] \tag{33}$$

$$\frac{d[\mathrm{Ald}]}{dt} = -k_1[\mathrm{Hy}][\mathrm{Ald}] + k_{-1}[\mathrm{Ho}] - k_2[\mathrm{Al}][\mathrm{Ald}] + k_{-2}[\mathrm{Ox}] \tag{34}$$

$$\frac{d[\mathrm{Ho}]}{dt} = +k_1[\mathrm{Hy}][\mathrm{Ald}] - k_{-1}[\mathrm{Ho}] \tag{35}$$

$$\frac{d[\mathrm{Al}]}{dt} = -k_2[\mathrm{Al}][\mathrm{Ald}] + k_{-2}[\mathrm{Ox}] \tag{36}$$

$$\frac{d[\mathrm{Ox}]}{dt} = +k_2[\mathrm{Al}][\mathrm{Ald}] - k_{-2}[\mathrm{Ox}] \tag{37}$$

Solving the ODEs for both cases by running multiple simulations with different sets of k_n, and at different initial equimolar concentration of $[\mathrm{Ho}]_0$=$[\mathrm{Al}]_0$, gives complex datasets. These are fitted to second-order irreversible or reversible kinetics depending on the value of k_{-2}. Reversible Dirksen model[1] applied for high and medium values, irreversible model for low and zero values (exact values in Table S2). For each defined set of k_n, the series k_{obs} plotted against the used $[\mathrm{Ho}]_0 = [\mathrm{Al}]_0$, and fitted to the Fourth Party Logistic function (4PL), allows a detailed evaluation of the influence of the k_n on the parameters of the function (Figure S9B-E). The equation takes the form:

$$k_{\mathrm{obs}} = \frac{k_2}{1 + \left(\frac{[\mathrm{Ho}]_0}{a}\right)^b} \tag{38}$$

It results that the parameter a has a direct proportionality with k_{-1} and an inverse proportionality with k_1 and k_2. Changing K_a either by increasing k_1 or decreasing k_{-1} impacts the location of the sigmoidal curve dramatically (Figure S9B-C), while decreasing by an equal factor both k_1 and k_{-1}, thus maintaining the same K_a, has a minor effect (Figure S9D), meaning that the thermodynamics of the hydrazone hydrolysis plays a more important role than the kinetics of hydrolysis. Changing k_2 results in a dramatic change of the plateau (Figure S9E), as expected. We also evaluated k_{obs} against $[\mathrm{Ho}]_0$ for three different values of k_{-2}, obtaining pseudo-sigmoidal curves (Figure S9F). However, the higher the value of k_{-2}, the smaller is the range of $[\mathrm{Ho}]_0$ where the fitting to obtain k_{obs} is possible. Thus, for high values of k_{-2} a more advanced model is required. For our system that involves a final highly stable oxime, further development of the model was out of scope.

Supplementary Tables

Table S1. List of the molecular reactions in water studied by HPLC.

entry	dynamic reaction	catalyst	pH[c)]	k_{obs} (L·mol^{-1}·s^{-1})[d)]
1	1-Ald + 1-Hy → 1-Ho	no	7.00 ± 0.1	n.d.
2	1-Ald + 3-Al → 3-Ox	no	7.00 ± 0.1	0.136 ± 0.016
3a	1-Ho + 1-Al → 1-Ox + 1-Hy	yes	7.00 ± 0.1	0.0111 ± 0.0006
3b	1-Ho + 1-Al → 1-Ox + 1-Hy	no	7.00 ± 0.1	0.0093 ± 0.0021
3c	1-Ho + 1-Al → 1-Ox + 1-Hy	no	6.00 ± 0.1	0.0746 ± 0.0062
3d	1-Ho + 1-Al → 1-Ox + 1-Hy	no	8.00 ± 0.1	0.0059 ± 0.0008
4	1-Ho + 2-Al → 2-Ox + 1-Hy	yes	7.00 ± 0.1	0.0193 ± 0.0034
5[a)]	2-Ho[b)] + 3-Al → 3-Ox + 2-Hy	no	7.00 ± 0.1	0.0032 ± 0.0010[e)]<br>0.0301 ± 0.0143[f)]

[a)] Performed at 10 mM and 2 mM, sample prepared with the same generic procedure described above, adjusting the aliquot of the stock solution of 2-Ho, and the quantity of 3-Al; [b)] Prepared by leaving combined 1-Ald (200 mM) and 2-Hy (100 mM) to equilibrate for 1 day; [c)] The value of pH refers to the prepared solutions of 1-Hy or 3-Al in buffer (with or without catalyst) at the final concentration of 10 mM, and to the reactant stock solution (when this is buffer, as for 1-Al and 2-Al). The pH was always adjusted at the same desired value. The error represents the average maximum fluctuation of pH value observed when the reactant is added from stock solution in DMSO (1-Ald or 2-Ho); [d)] Determined by fitting the second-order irreversible kinetic model, with reported mean values ± the standard deviations for triplicates; [e)] At 10 mM; [f)] At 2mM.

Table S2. Kinetic constants as parameters for the kinetic simulations. Medium values are determined experimentally (except for k_{-2}). High and low values are chosen arbitrarily with 10-fold difference compared to the medium values.

	k_1 (L·mol^{-1}·s^{-1})	k_{-1} (10^{-3}·s^{-1})	k_2 (L·mol^{-1}·s^{-1})	k_{-2} (10^{-7}·s^{-1})
low	0.064	0.086	0.012	0.015
Medium	0.64[a)]	0.86[a)]	0.12[b)]	0.15[c)]
high	6.4	8.6	1.2	1.5

[a)] Values obtained from the second-order reversible fitting of the hydrazone formation monitored by UV-Vis spectroscopy (Figure S7). [b)] Value obtained from the second-order irreversible fitting of experimental data of the entry 2 in Table S1 (direct oxime formation analyzed by HPLC). [c)] Chosen arbitrarily.

Table S3. List of gelation experiments, conditions, and extracted parameters.

				Time sweeps		Stress relaxations	
entry	system	catalyst	pH[a)]	t_{cross} (min)[b)]	G' (Pa) \ t (min)[c)]	τ (s)	G (t = 24 h) / G (t = 0 h)
1a	PEG-2Ho	yes	7.00 ± 0.1	0.7	1619 ± 324 \ 10	277	n.d.
1b	PEG-2Ho	no	7.00 ± 0.1	7	2653 ± 389 \ 60	2622	n.d.
1c	PEG-2Ho	no	6.00 ± 0.1	0.7	2844 \ 10	147	n.d.
1d	PEG-2Ho	no	8.00 ± 0.1	31 ± 7	1684 \ 120	7821	n.d.
2a	PEG-2Ox, direct	yes	7.00 ± 0.1	n.d.	4541 ± 676 \ 10	46369	n.d.
2b	PEG-2Ox, direct	no	7.00 ± 0.1	0.4 ± 0.3	4334 ± 1256 \ 45	n.d.	0.89
2c	PEG-2Ox, direct	no	6.00 ± 0.1	0.3 ± 0.1	3330 \ 10	17007	n.d.
2d	PEG-2Ox, direct	no	8.00 ± 0.1	5 ± 2	1241 \ 60	n.d.	0.78

3a	PEG-2Ox, exchange	yes	7.00 ± 0.1	132 ± 25	1696 \ 600	25198	n.d.
3b	PEG-2Ox, exchange	no	7.00 ± 0.1	238 ± 5	5878 ± 674 \ 1440	n.d.	0.73
3c	PEG-2Ox, exchange	no	6.00 ± 0.1	34 ± 3	2487 \ 300	16076	n.d.
3d	PEG-2Ox, exchange	no	8.00 ± 0.1	840 ± 80	2510 \ 4320	n.d.	0.78
4a	PEG-4Ox, direct	yes	7.00 ± 0.1	0.3	5938 \ 30	n.d.	n.d.
4b	PEG-4Ox, direct	no	7.00 ± 0.1	0.7	6717 \ 60	n.d.	n.d.
5a	PEG-4Ox, exchange	yes	7.00 ± 0.1	542	3417 \ 2400	n.d.	n.d.
5b	PEG-4Ox, exchange	no	7.00 ± 0.1	1391 ± 6	2842 \ 4320	n.d.	n.d.
6	PEG-2Ox, competition	no	7.00 ± 0.1	42 ± 6	2313 \ 840	n.d.	n.d.
7	PEG-4Ox, competition	no	7.00 ± 0.1	858 ± 66	2880 \ 3600	n.d.	n.d.

[a] The value of pH refers to the pristine buffer (without or with catalyst) and crosslinker stock solutions (always adjusted to the same desired value). The error represents the average maximum discrepancy of pH values between the buffer and the crosslinker stock solutions used. It was verified that the polymer has negligible influence on the pH, and that the conversion to hydrazone or oxime has negligible impact on the pH. [b] Time where the storage modulus surpasses the loss modulus (*i.e.*, $G' > G''$), with a threshold of $G' \geqslant 10$ Pa to consider instrumental errors. [c] Time, in the time sweep measurement, at which the value G' was taken, dependent on the kinetic of gelation. Values with errors are means ± the standard deviations for triplicates.

Table S4. List of topological reconfiguration experiments. All the systems PEG-2Ox and PEG-4Ox refer to reconfigured gels (diffusion-exchange process) from the initial fabricated PEG-2Ho.

entry	system	reconfiguration	Hy (eq.)[a]	Al (eq.)[a]	G_{start}' (Pa)[d]	G_{end}' (Pa) \ t (min)[e]
1	PEG-2Ho	*ex-situ* (vial)	1+1[b]	n.d.	n.d.	n.d. (sol)[f]
2a	PEG-2Ox	*ex-situ* (vial)	1	1	n.d.	n.d. (gel)[f]
2b	PEG-2Ox	*ex-situ* (vial)	1	2	n.d.	n.d. (sol)[f]
3a	PEG-2Ox	*in-situ*	1	1	2134 ± 324	3896 \ 600
3bI	PEG-2Ox	*ex-situ* (mold)	1	1	n.d.	2598[g]
3bII	PEG-2Ox	*ex-situ* (mold)	1	1	n.d.	2588[g]
3c	PEG-2Ox	*in-situ*	1	1.45	2506 ± 553	236 ± 147 \ 600
3d	PEG-2Ox	*in-situ*	1.15	1	649 ± 253	2639 ± 568 \ 600
3e	PEG-2Ox	*in-situ*	0.5 (+0.5 Al)[c]	0.5	4742	4889
4	PEG-4Ox	*in-situ*	1	1	2149	2646 \ 4000

[a] hydrazine crosslinker (2-Hy) used for gelation, and alkoxyamine crosslinker (2-Al or 4-Al) used for reconfiguration; [b] The second equivalent of the crosslinker was added to the hydrazone gel formed with the first equivalent of crosslinker; [c] The initial (hydrazone) gel was fabricated using mixed crosslinkers; [d] Value of G' of the starting hydrazone gel; [e] Value of G' of the final, reconfigured, oxime gel. [f] Visual assessment after leaving the diffusion-exchange process to occur overnight. [g] Measured after 5 days of reconfiguration. Values with errors are means ± the standard deviations for triplicates. For the reconfigurations using 1 eq. Hy and 1 eq. Al (entries 3a-b), both *in-situ* and *ex-situ* experiments were considered to determine the mean and standard deviation of the final reconfigured oxime gel, which results in $G_{end}' = (3027 \pm 752)$ Pa.

Supplementary Figures

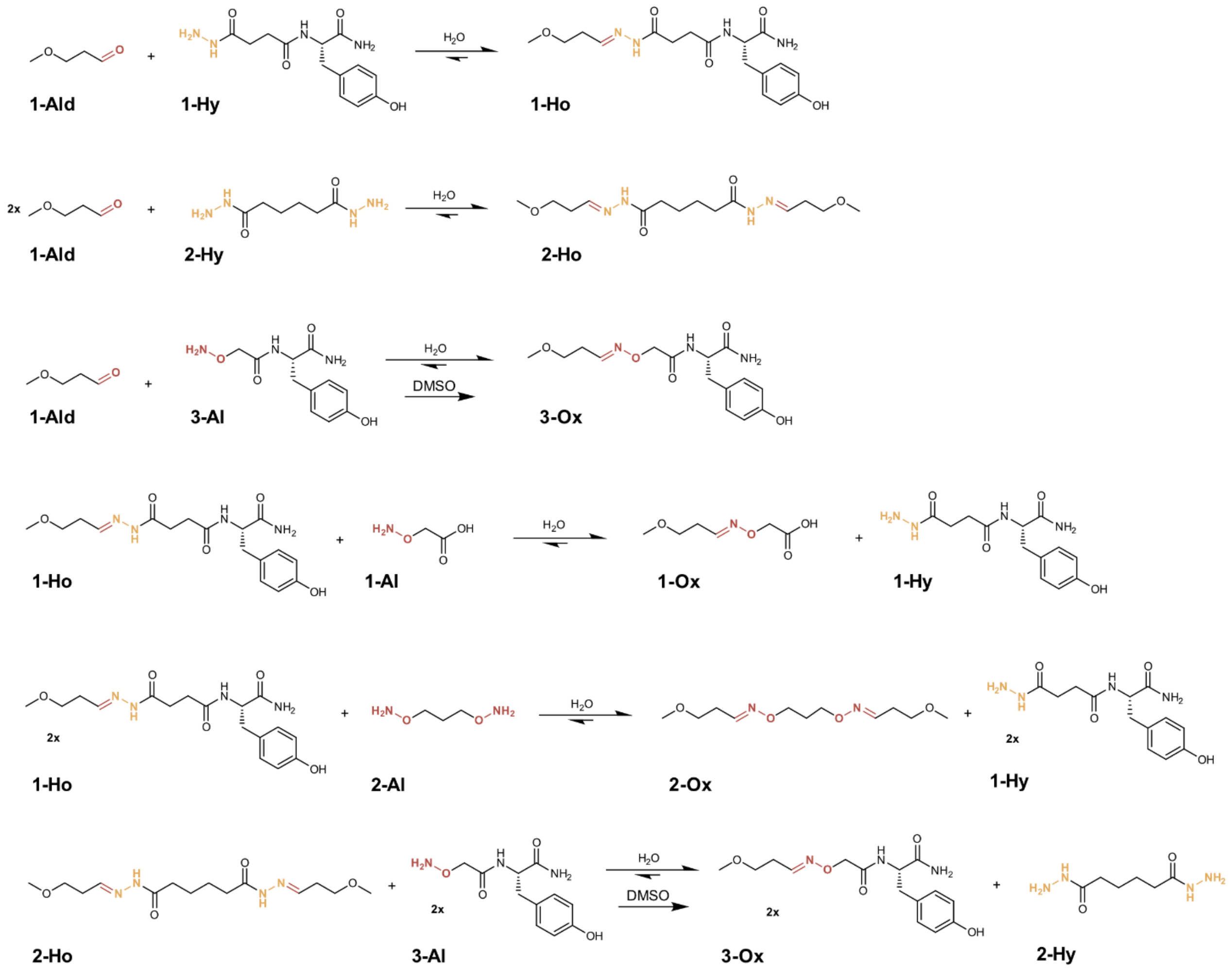

Figure S1. Full list of the molecular reactions studied by HPLC or UV-Vis.

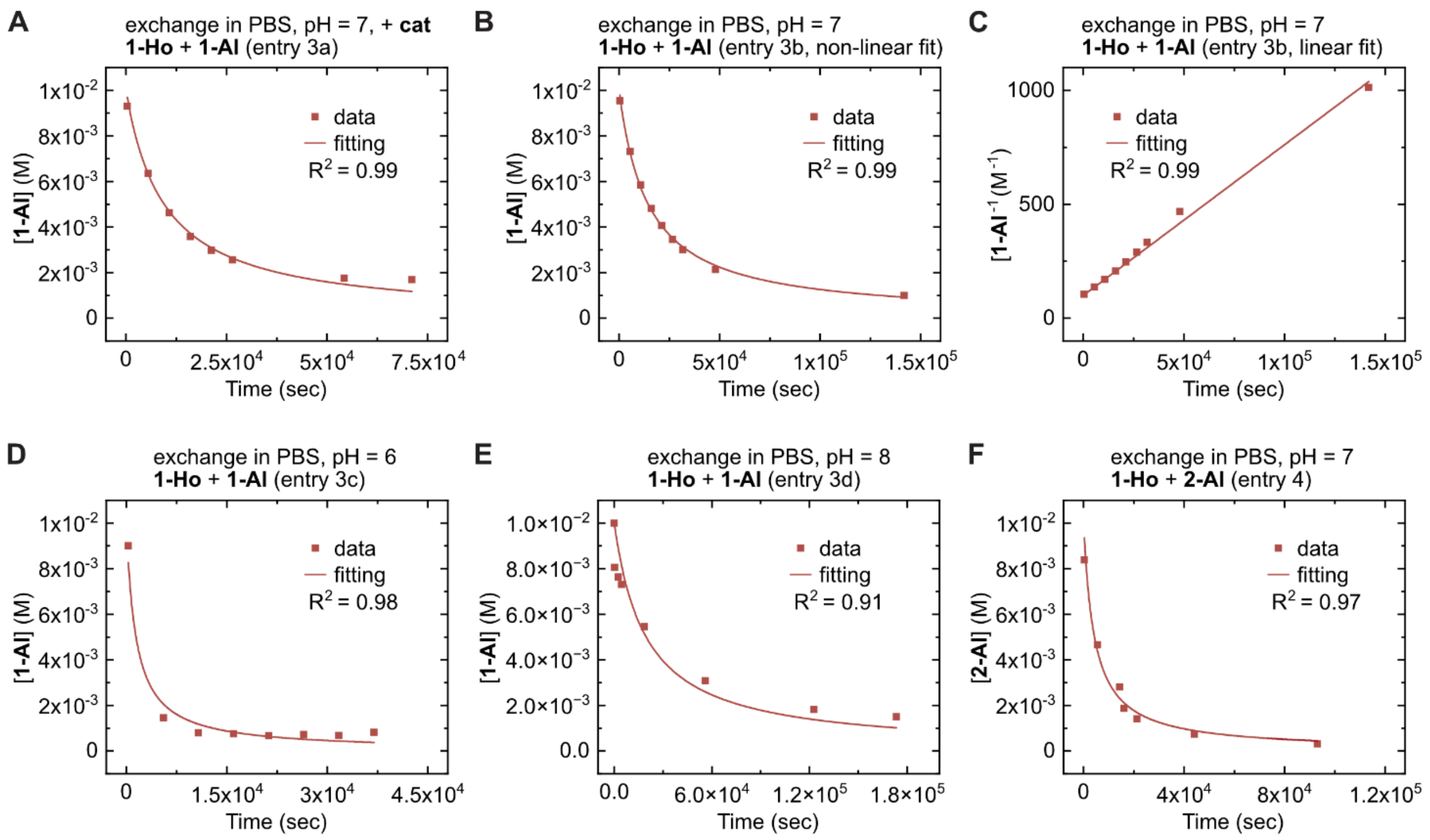

Figure S2. **Kinetic analysis of the experimental data for the quantitative assessment of the exchange.** A-F) Representative kinetic fits (second-order irreversible) obtained with processed HPLC data of the dynamic reactions in Table S1, as indicated. Consumption of the alkoxyamine derived from the signals of 1-Hy. All the data fit well to the second-order irreversible kinetic model.

Note. The fitting to the second-order irreversible kinetic model was performed for the exchange 1-Ho + 1-Al (entry 3b) in both linearized and non-linear forms. Values of k_{obs} were found to be comparable, with the R^2 of the non-linear fitting a little higher. Considering this observation, and the approximative nature of the model, we decided to apply the non-linear fitting to obtain all the values of k_{obs} that are intended to be indicative kinetic constants for all the exchange processes that are in reality more complex as they integrate multiple equilibrium reactions. Note that in every fitting, the parameter $[A]_0$ was preset manually.

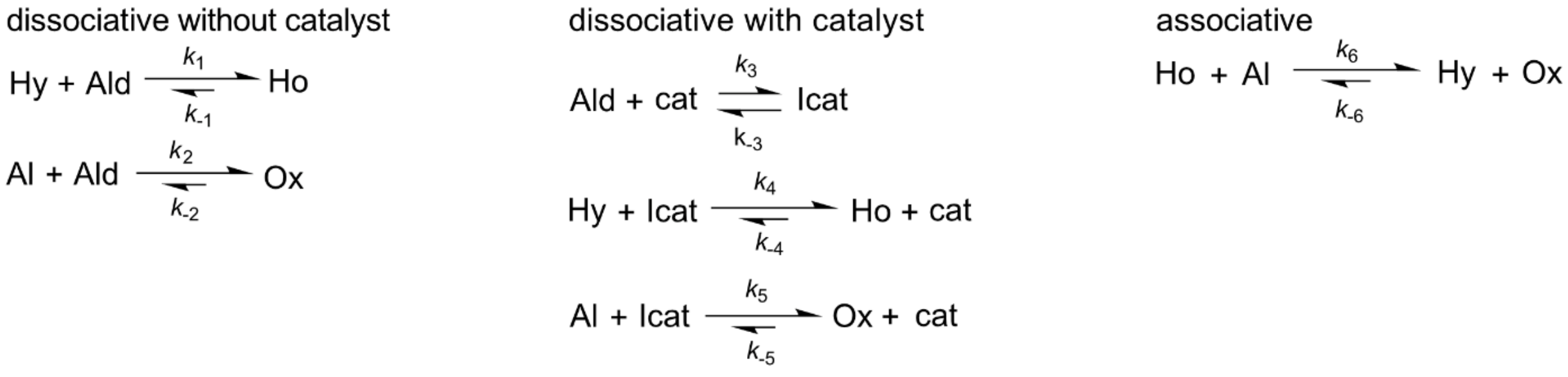

Figure S3. Full kinetic model.

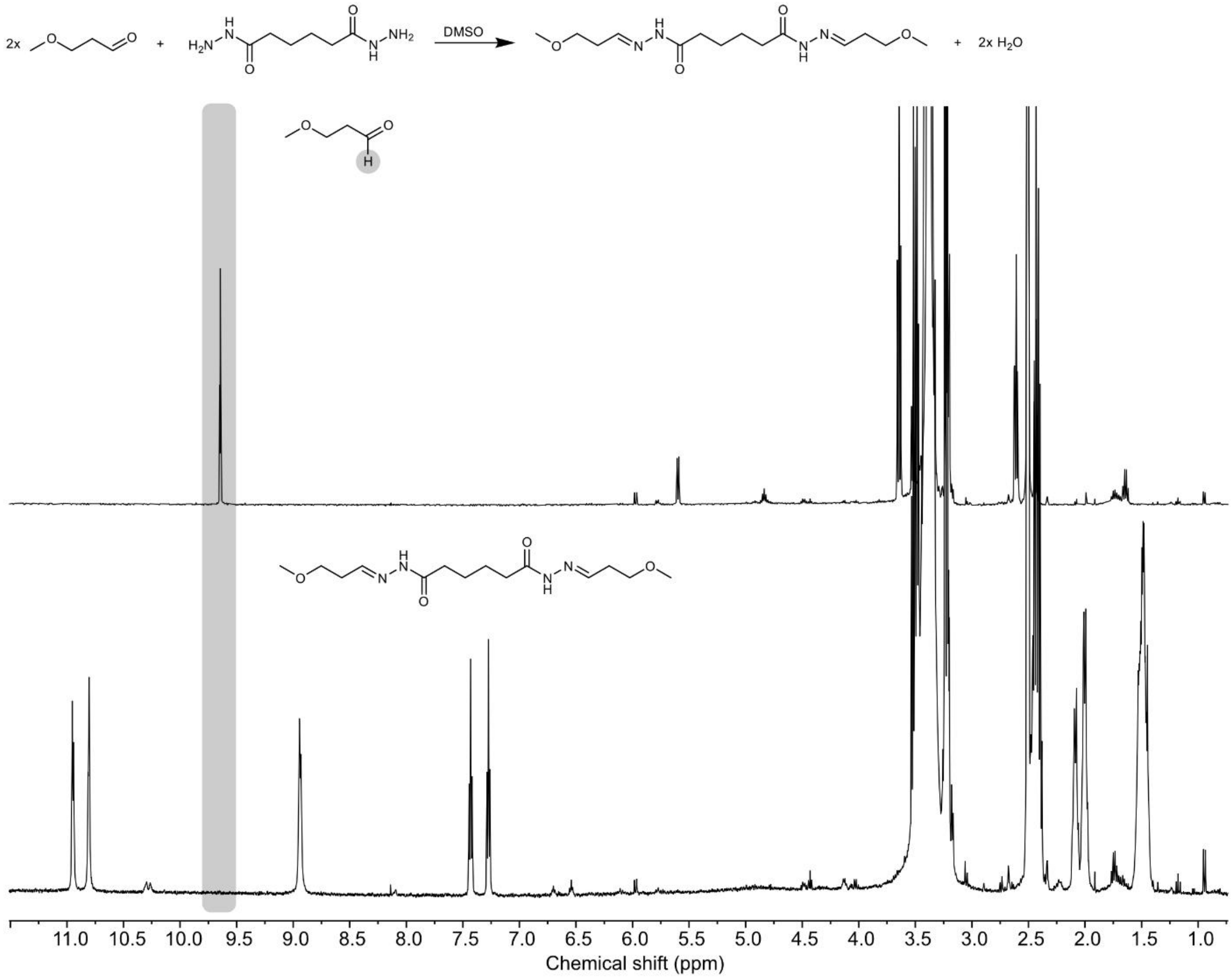

Figure S4. Reaction scheme of the formation of 2-Ho in DMSO (top) and related stacked ^{1}H NMR spectra in DMSO-d_6 of the starting 1-Ald versus the obtained 2-Ho. The gray area highlights the disappearance of the diagnostic aldehyde proton.

Note. 2-Ho (100 mM in DMSO) was prepared by reaction of 2-Hy (100 mM) and 1-Ald (200 mM) for 18 hours. High concentration allows quick formation, and DMSO prevents hydrolysis, affording the hydrazone quantitatively.

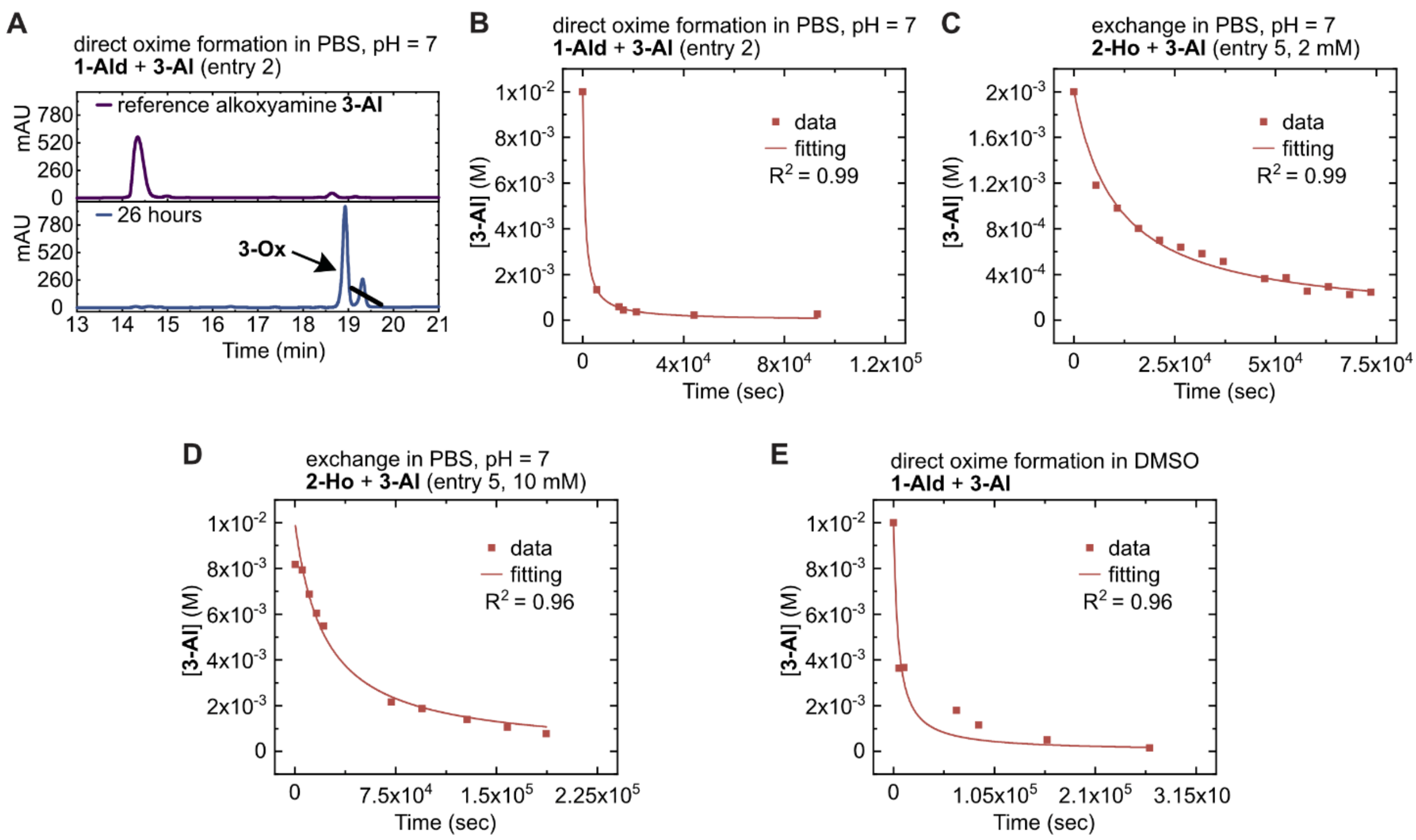

Figure S5. **Kinetic analysis of the exchange and of the direct oxime formation to extract mechanistic information.** A) Representative chromatogram for the direct formation of oxime 3-Ox in water, as indicated. All the exchange reactions from 3-Al to 3-Ox were followed in the same way. B-E) Representative kinetic fits (second-order irreversible) obtained with processed HPLC data of the dynamic reactions in Table S1, as indicated. E) Kinetic fit (second-order irreversible) obtained with processed HPLC data of the direct oxime formation in DMSO, as indicated.

Note. For the direct oxime formation (1-Ald + 3-Al) in DMSO, k_{obs} = (0.0331 ± 0.0187) L·mol^{-1}·s^{-1}.

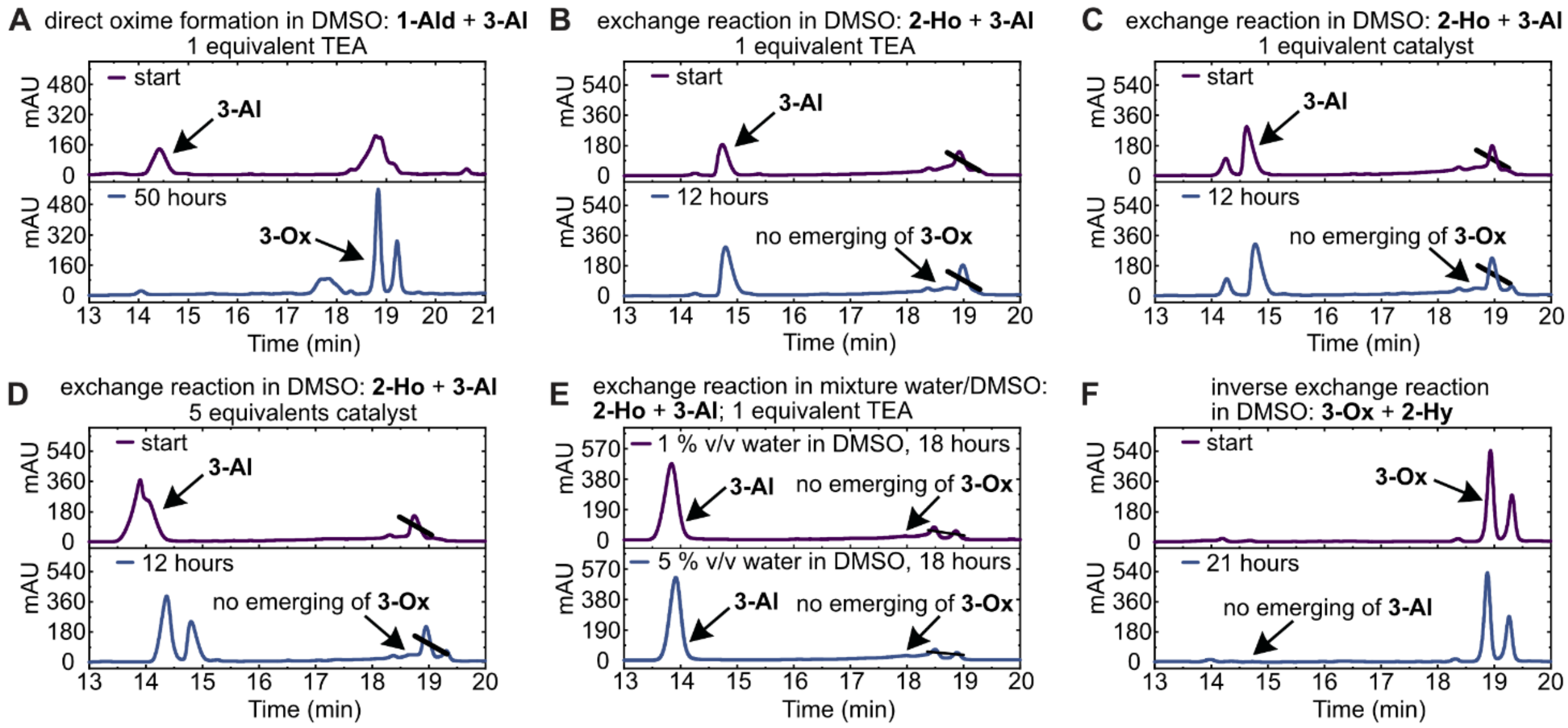

Figure S6. **Stacked HPLC qualitative analysis of the exchange and direct oxime formation in DMSO.** A) Chromatograms for the direct oxime formation. B-E) Chromatograms for the attempted exchange reaction in different conditions. No signal of 3-Ox appears. F) Chromatograms for the attempted reverse exchange reaction between a pre-formed 3-Ox and 2-Hy. No signal of 3-Al appears. The chromatograms titled as "start" represent the first injection immediately after having started the reaction.

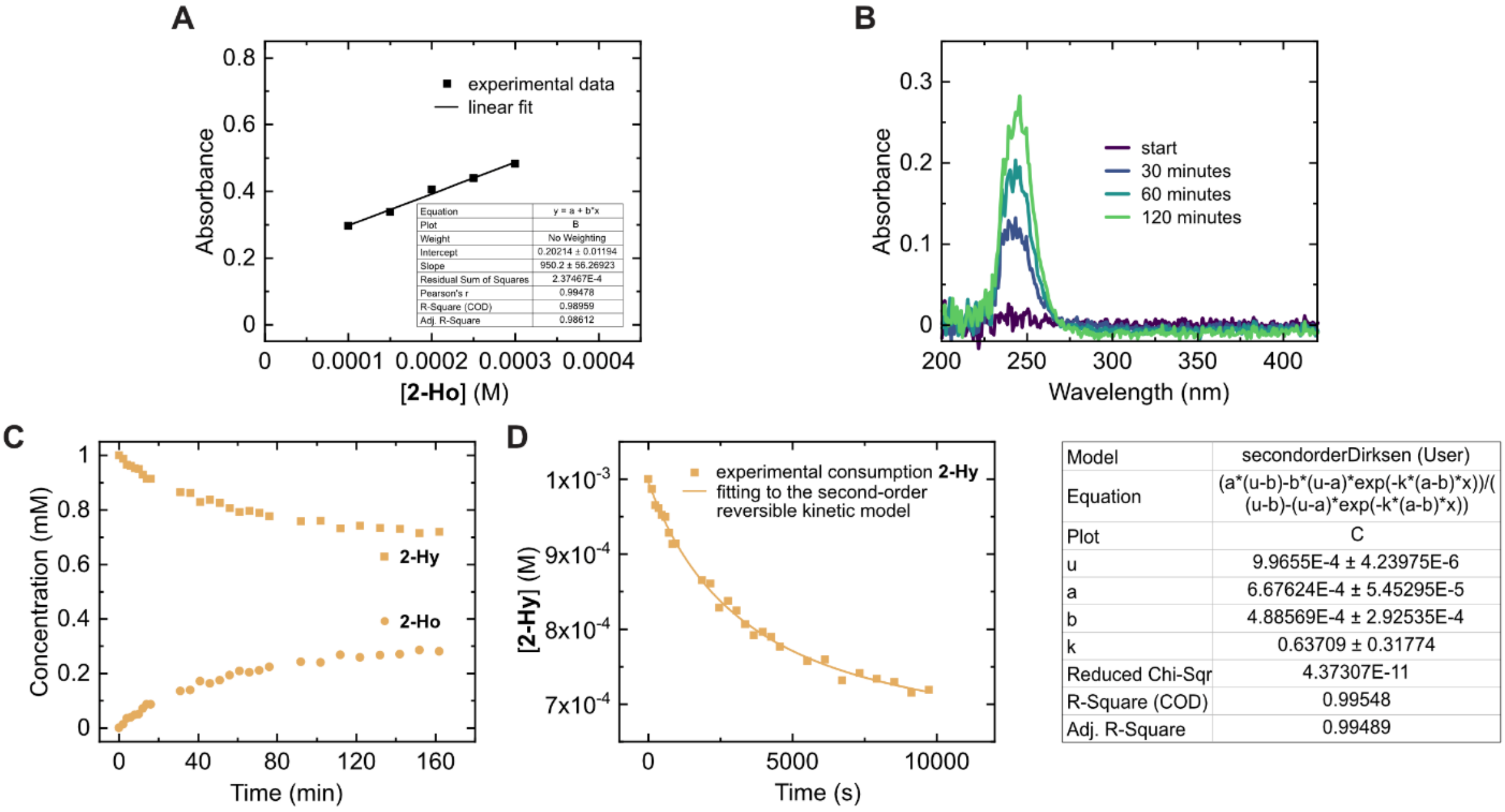

Figure S7. **UV-Vis kinetic analysis for the formation of the hydrazone 2-Ho to determine the kinetic parameters k_1 and k_{-1}.** A) Calibration to determine the extinction coefficient ε of 2-Ho. Obtained $\varepsilon = 950$. B) Representative stack of UV-Vis absorption spectra of the course of formation of the hydrazone 2-Ho. C) Derived full reaction progress for the formation of 2-Ho. D) Zoomed consumption of 2-Hy fitted to the second-order reversible kinetics.

Note. The magnitude of the rate of hydrazone hydrolysis requires the fitting to the second-order reversible kinetics, unlike the oxime. For the formation of 2-Ho by condensation of 2-Hy and 1-Ald we obtained the following kinetic parameters: $k_1 = (0.6 \pm 0.3)\ \mathrm{L \cdot mol^{-1} \cdot s^{-1}}$, $k_{-1} = (0.0009 \pm 0.0002)\ \mathrm{s^{-1}}$. And the derived equilibrium constant is: $K_a = (0.7 \pm 0.4)\ 10^3 \cdot \mathrm{L \cdot mol^{-1}}$.

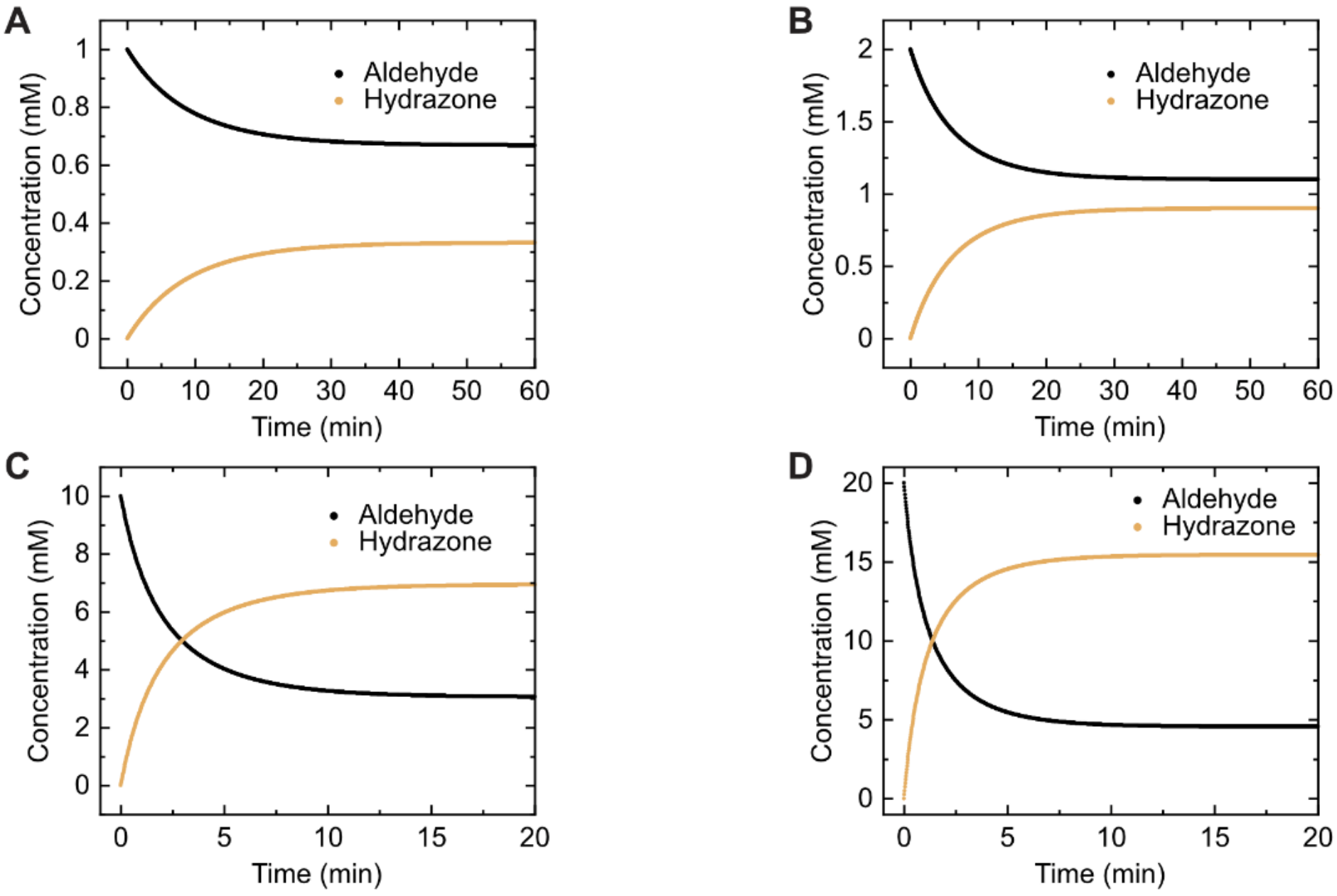

Figure S8. **Numerical solutions for the simulation of the hydrazone formation at different initial equimolar concentration of hydrazine and aldehyde**: A) 1 mM. B) 2 mM. C) 10 mM. D) 20 mM. The values of k_1, k_{-1}, and K_a, determined experimentally for the hydrazone 2-Ho formation, were used as parameters to obtain the numerical solutions.

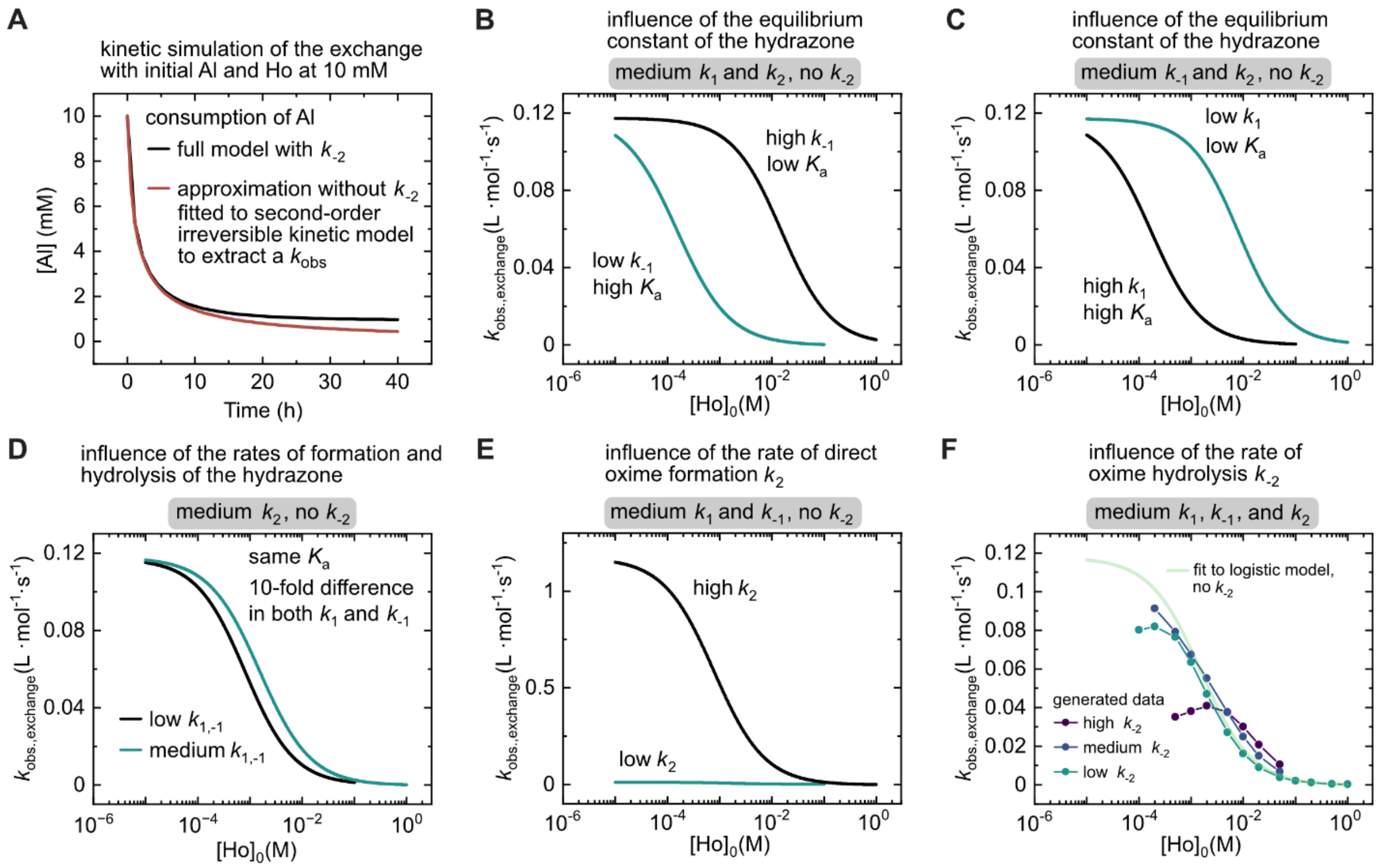

Figure S9. **Numerical simulations of the exchange reactions to define the kinetic framework.** A) Representative numerical solutions for the simulated consumption of Al using fixed kinetic parameters k_1, k_{-1}, k_2 (medium values in Table S2). Medium k_{-2} was used for the full model. Red line fits to second-order irreversible kinetics. Black line fits to second-order reversible kinetics. B-F) Simulated k_{obs} against $[Ho]_0$ (equal to $[Al]_0$), fitted to the logistic function, at different fixed kinetic parameters (indicated in the grey boxes, with corresponding values reported in Table S2).

Note. At least 7 values of $[Ho]_0$ were used to run the simulations at a given set of kinetic constants.

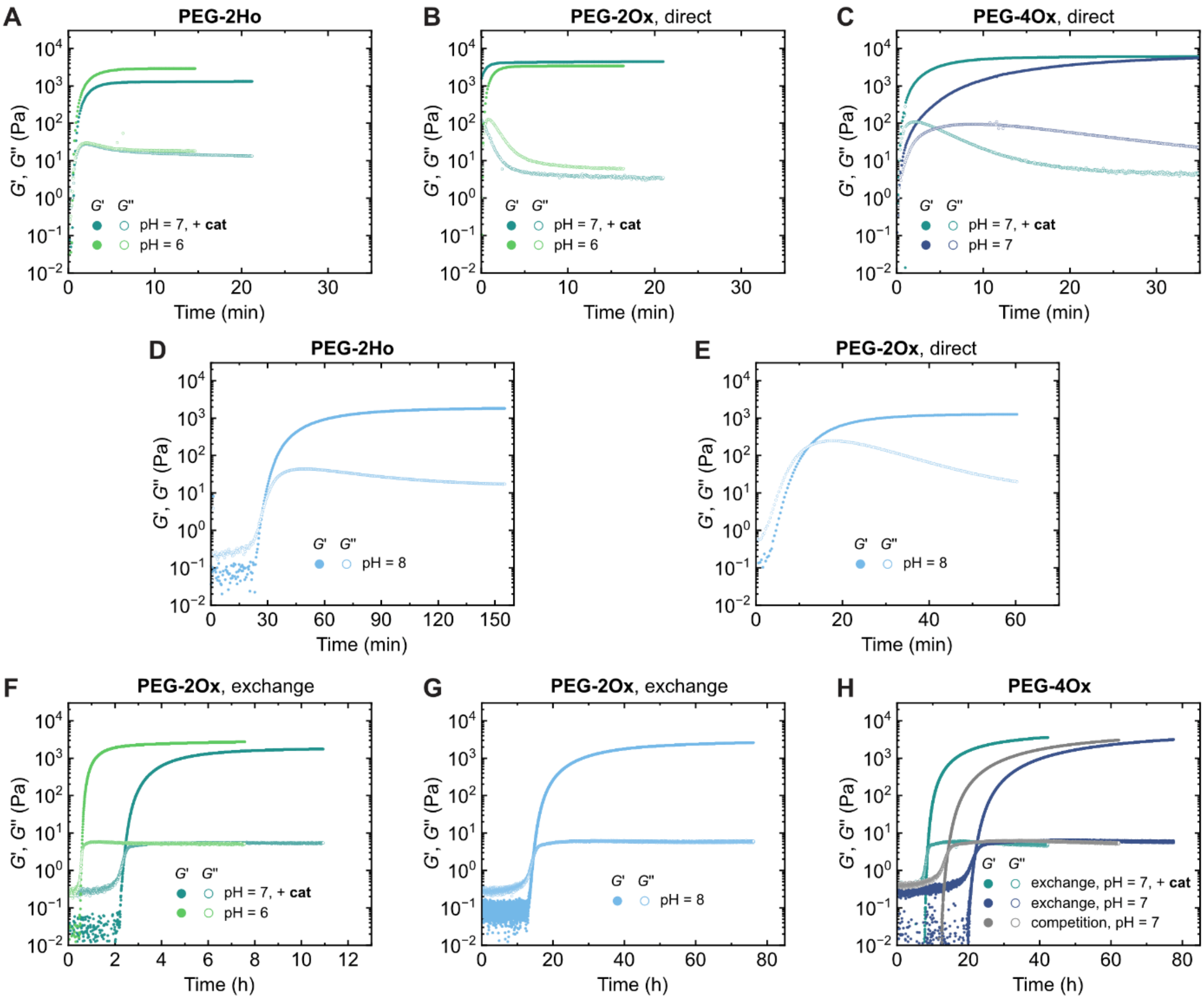

Figure S10. Representative time sweeps of the gels in Table S3, as indicated. A-E) Direct formations. F-H) Formations by exchange and competition. Gelation by exchange or competition is always significantly slower.

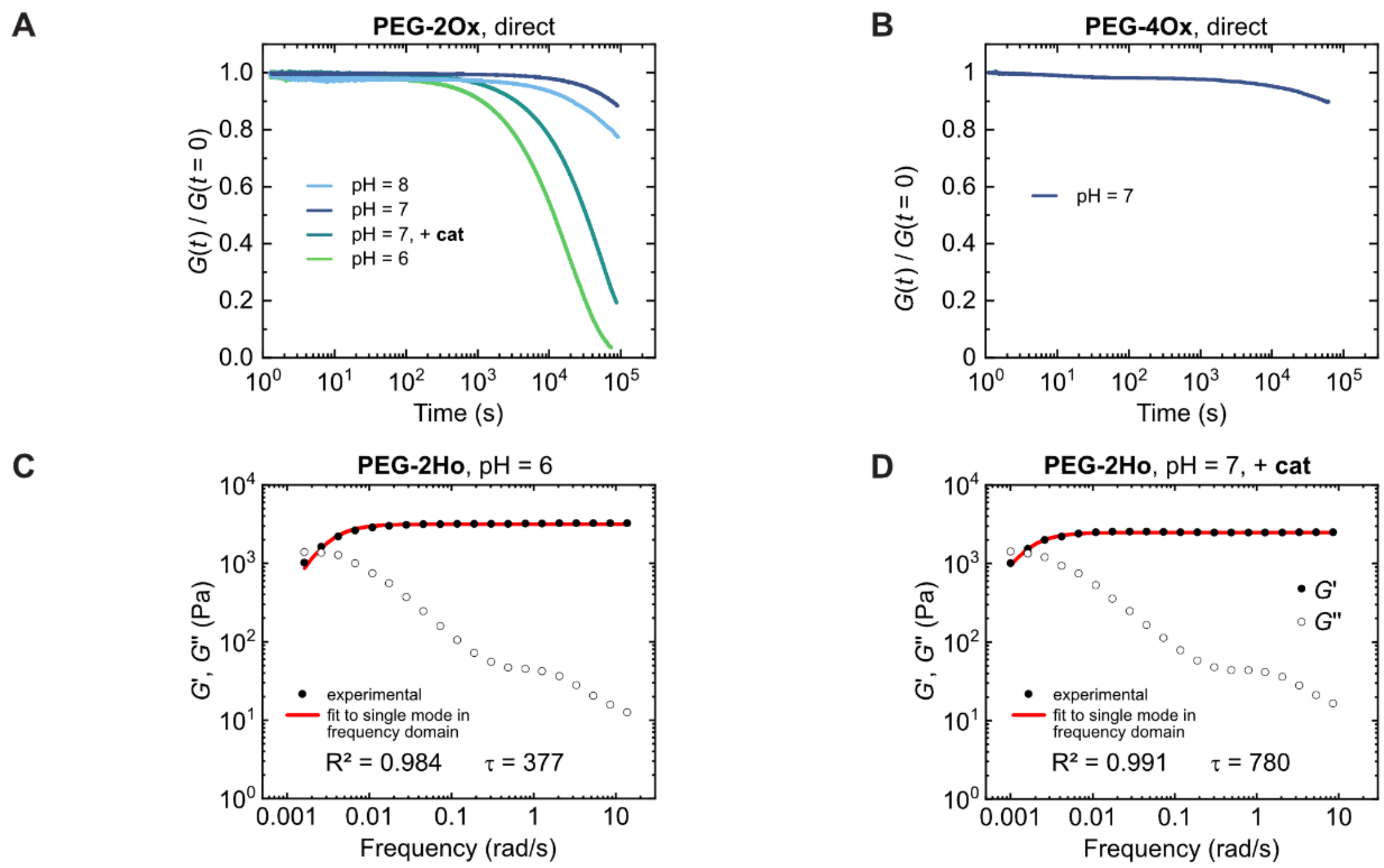

Figure S11. **Stress relaxation and frequency sweep analysis.** A-B) Stress relaxation curves of the gels in Table S3, as indicated. A) For PEG-2Ox formed directly. B) For PEG-4Ox formed directly. PEG-2Ox relaxes faster than PEG-4Ox. C-D) Frequency sweeps measurements, as indicated.

Note. As additional comparative analysis, the G' (ω) curves obtained from the frequency sweeps of the two fast relaxing hydrazone gels PEG-2Ho (pH 7 with catalyst, and pH 6) were fitted to the single mode Maxwell model in frequency domain, which takes the form:

$$G'(\omega) = G_0 \frac{(\omega\tau)^2}{1 + (\omega\tau)^2}$$

The characteristic relaxation times obtained are slightly higher than the ones obtained by fitting the stress relaxation curves (see Table S3), but nevertheless in the same order of magnitude.

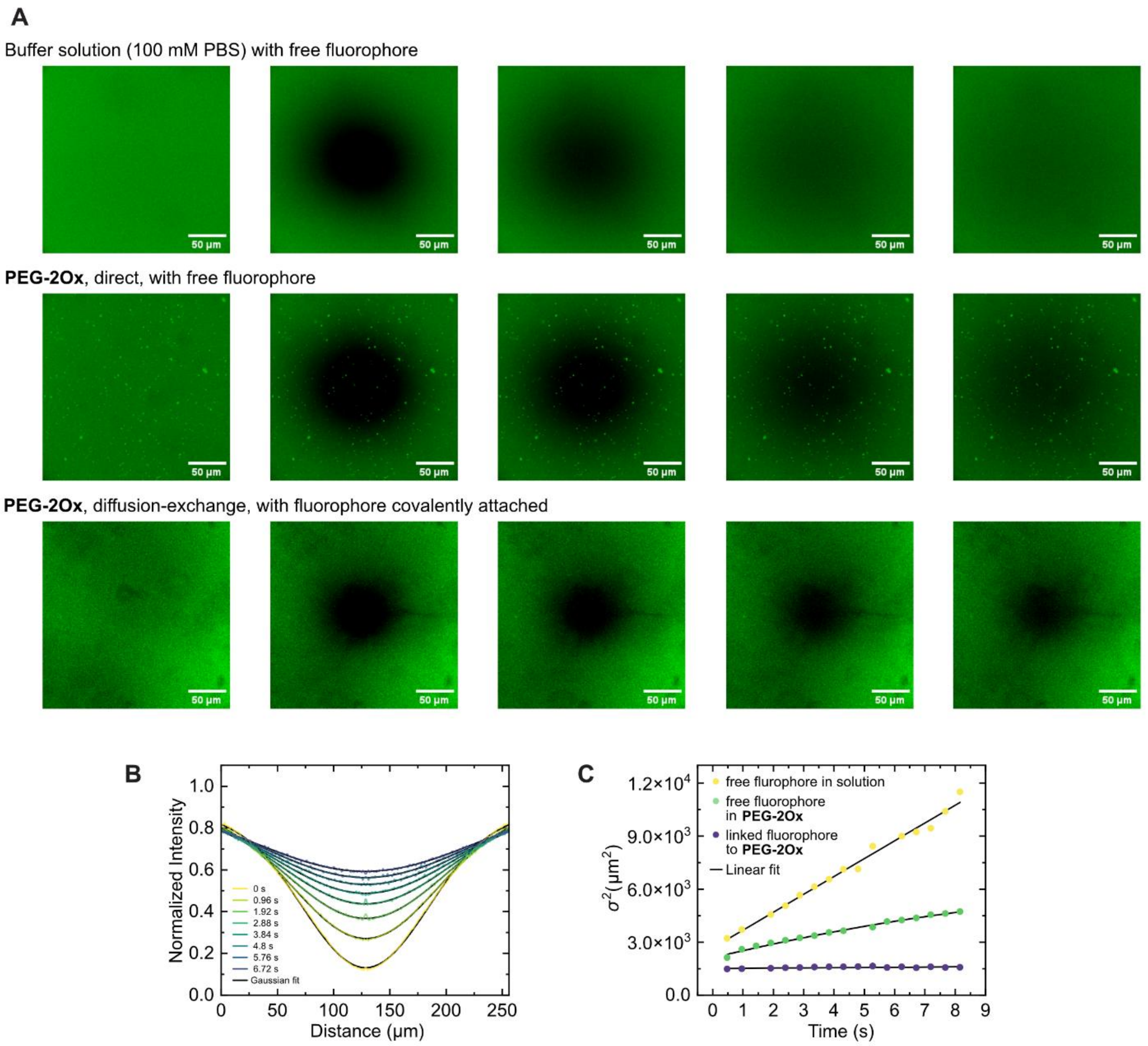

Figure S12. **FRAP analysis to determine the diffusion coefficients of the fluorophore in different environments.** A) Representative pre-bleached image (left) and post-bleached images at: 0.48, 2.4, 7.2, and 12 seconds. B) Representative cross-sectional line profiles at different time points for the fluorophore in the gel, giving a Gaussian distribution of the fluorescence intensity. The Gaussian fit yields the squared standard deviations (σ^2) at the different time points. C) Representative plots of the obtained σ^2 against time, for the fluorophore in solution, fluorophore in the gel, and for the linked fluorophore in the gel PEG-2Ox (diffusion-exchange), where the linear fit gives the slope from which the diffusion coefficient is extracted. The slope of the linear fits of PEG-2Ox with linked fluorophore are always close to zero due to the practical absence of diffusion within the experimental time range.

Note. To obtain the values of *D*, the fitting was always performed in the range of 0.48-8.16 seconds, as the increase of σ^2 deviates from linearity at higher recovery time.

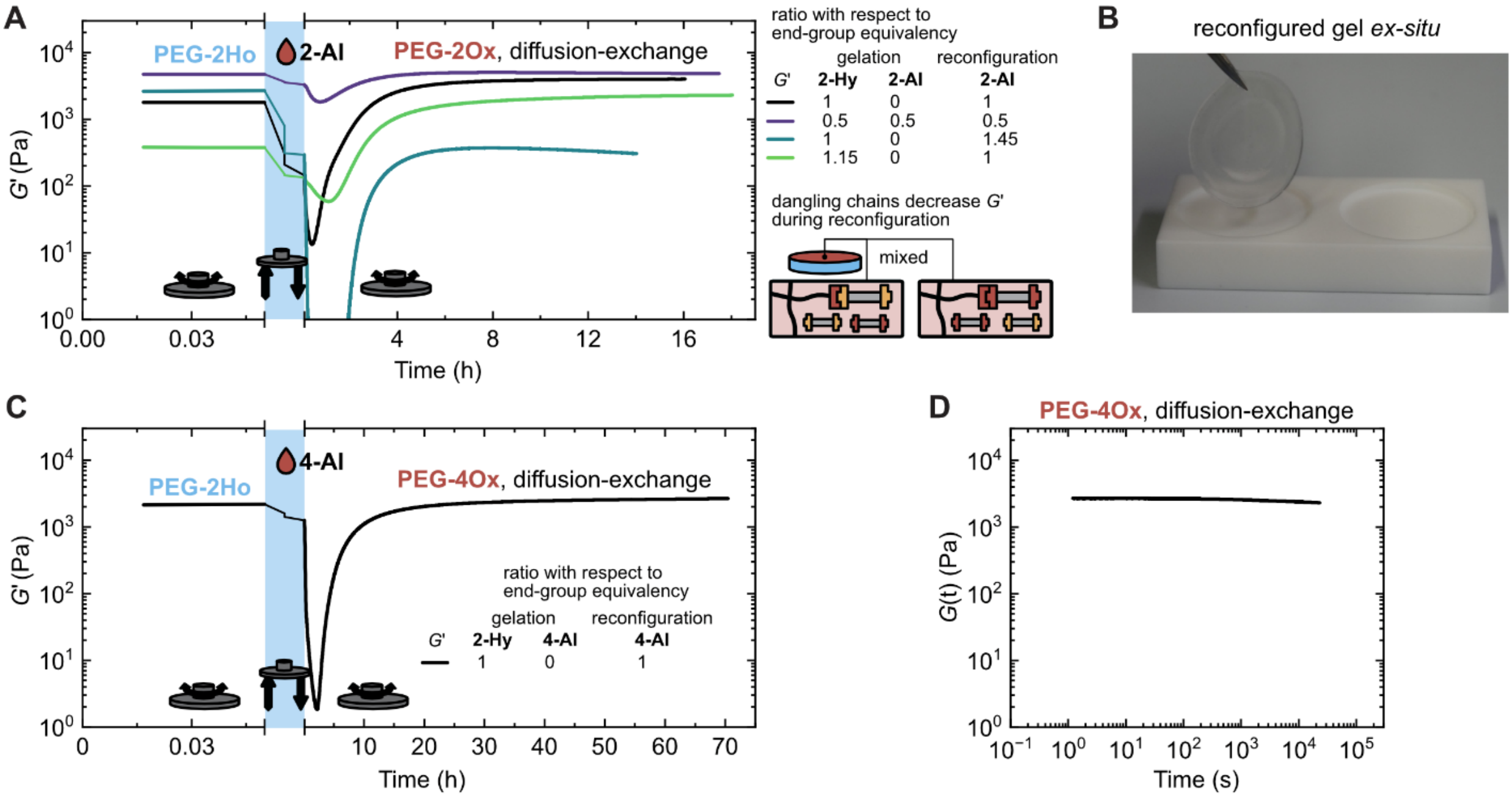

Figure S13. **Topological reconfigurations.** A) Representative time sweeps of the *in-situ* reconfigurations to PEG-2Ox, using different stoichiometries of hydrazine 2-Hy and alkoxyamine 2-Al. The pictogram summarizes the contribution of dangling chains to the transient softening after the addition of 2-Al. B) Oxime gel PEG-2Ox after 5 days of reconfiguration *ex-situ* using the crosslinkers 2-Hy and 2-Al with stoichiometry 1:1. After this time, the gel reaches a homogenous mechanics and showcases excellent structural integrity for safe removal from the mold, subsequent handling, and relocation in the rheometer for further analysis. C) Time sweep of the *in-situ* reconfiguration to PEG-4Ox, using the same stoichiometry of hydrazine 2-Hy and alkoxyamine 4-Al. D) Stress relaxation of the reconfigured oxime gel PEG-4Ox. Details in Table S4. The reconfigured oxime gel PEG-4Ox has a very long relaxation time, coherent with the high hydrolytic stability of the oxime bonds. This confirms the occurred exchange resulting predominantly in oxime crosslinks.

Note. This dip in *G′* arises from the exchange process itself, which transiently generates dangling chains and a corresponding reduction in load-bearing crosslinks. Such transient softening is markedly reduced when starting from a gel fabricated with equimolar hydrazine and alkoxyamine crosslinkers (0.5 equivalents each) and subsequently adding an additional 0.5 equivalent of alkoxyamine (Figure S13A, purple line). This is expected due to the presence of crosslinks that are not reconfigured. In addition, the higher initial *G′* reflects the contribution of oxime crosslinks. A parallel *ex-situ* reconfiguration demonstrates structural integrity of the bulk material and safe removal from the mold after the process (Figure S13B). Performing the 1:1 exchange with alkoxyamine 4-Al leads to a similar transient dip, but with a prolonged transient regime, consistent with the slower exchange kinetics of this crosslinker (Figure S13C).

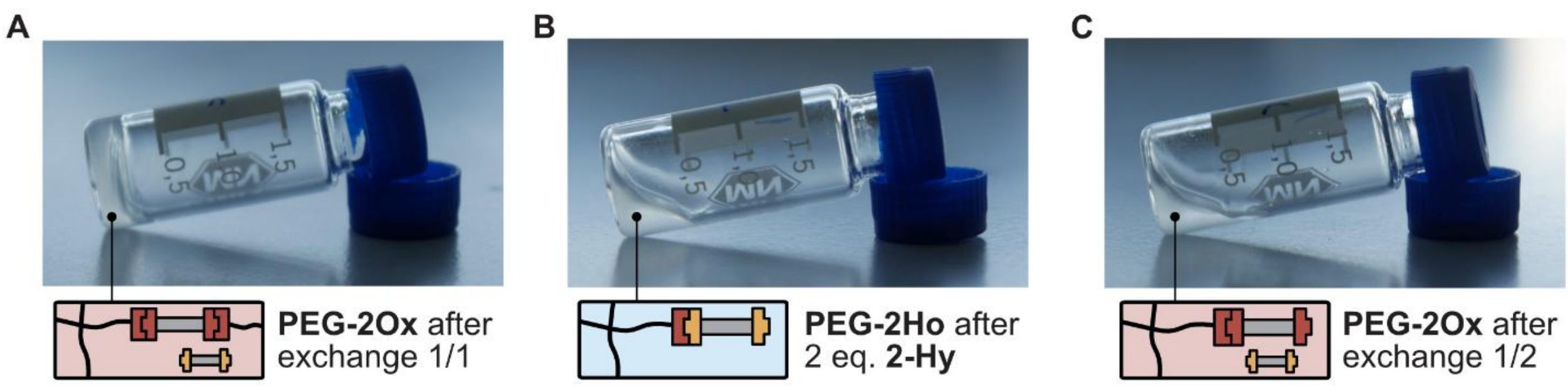

Figure S14. **Topological reconfigurations in vials using large stoichiometric excesses of the crosslinkers, as indicated. Details in Table S4**. A) Hydrogel maintains structural integrity. B-C) Gel-sol transitions with excesses of either crosslinker.

Characterization spectra

Compound **1**: ^{1}H NMR (400 MHz, DMSO-d_6)

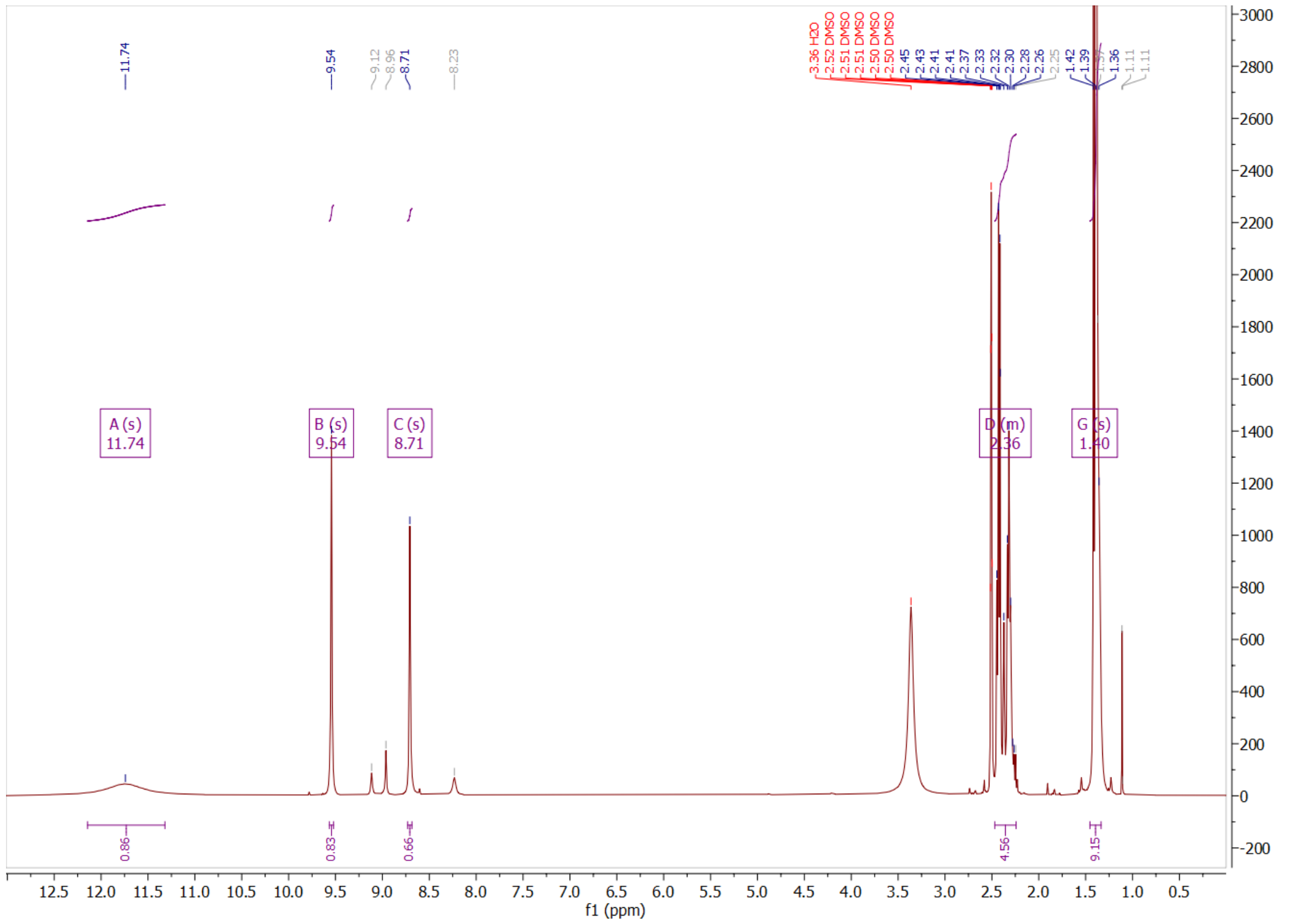

Compound **1**: ^{13}C NMR (75 MHz, DMSO-d_6)

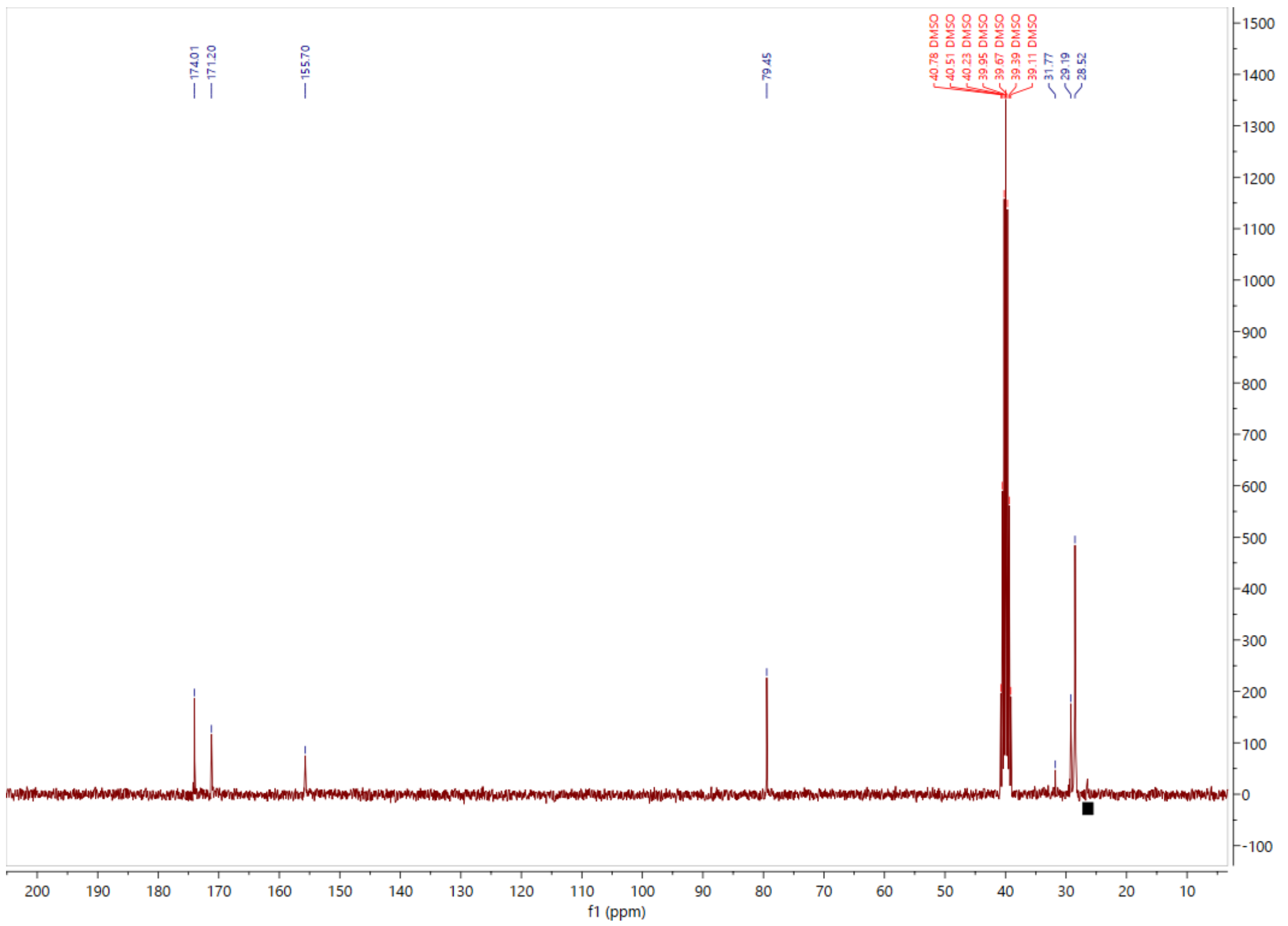

Note. Black square indicates unidentified impurity signal.

Compound **2**: ^{1}H NMR (400 MHz, DMSO-d_6)

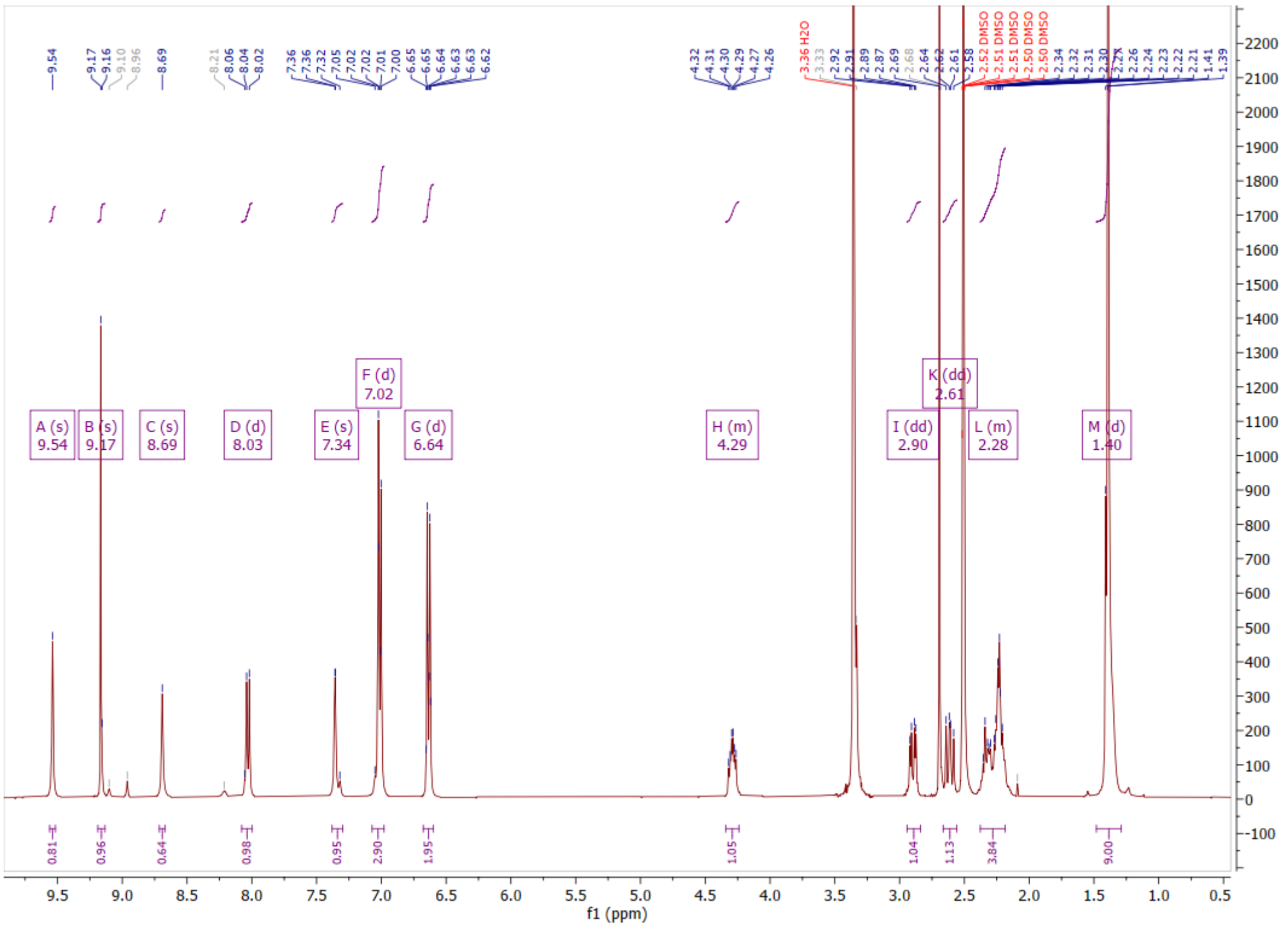

Compound **2**: ^{13}C NMR (75 MHz, DMSO-d_6)

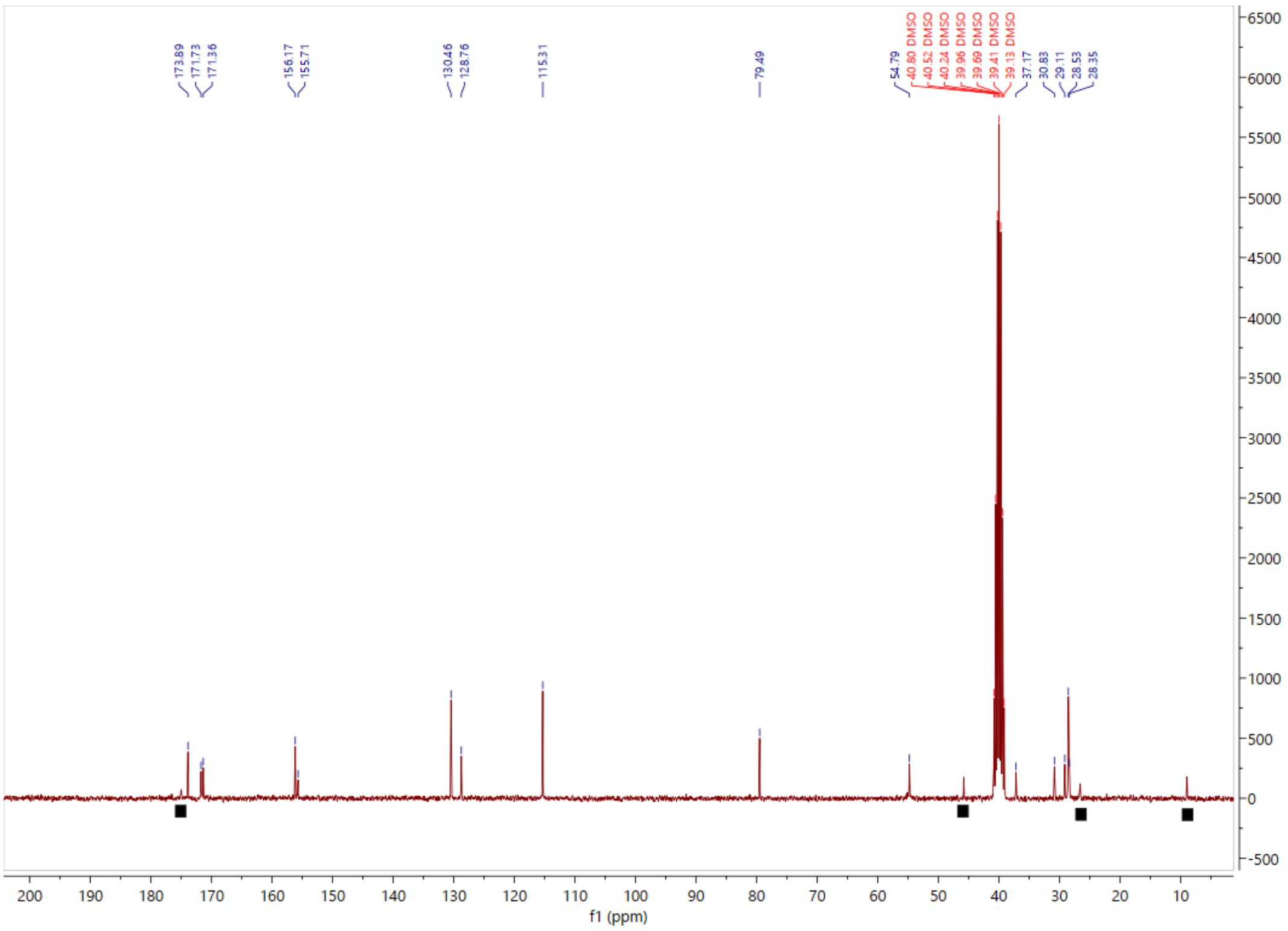

Note. Black squares indicate unidentified impurities signals.

Compound **1-Hy**: ^{1}H NMR (400 MHz, D_2O)

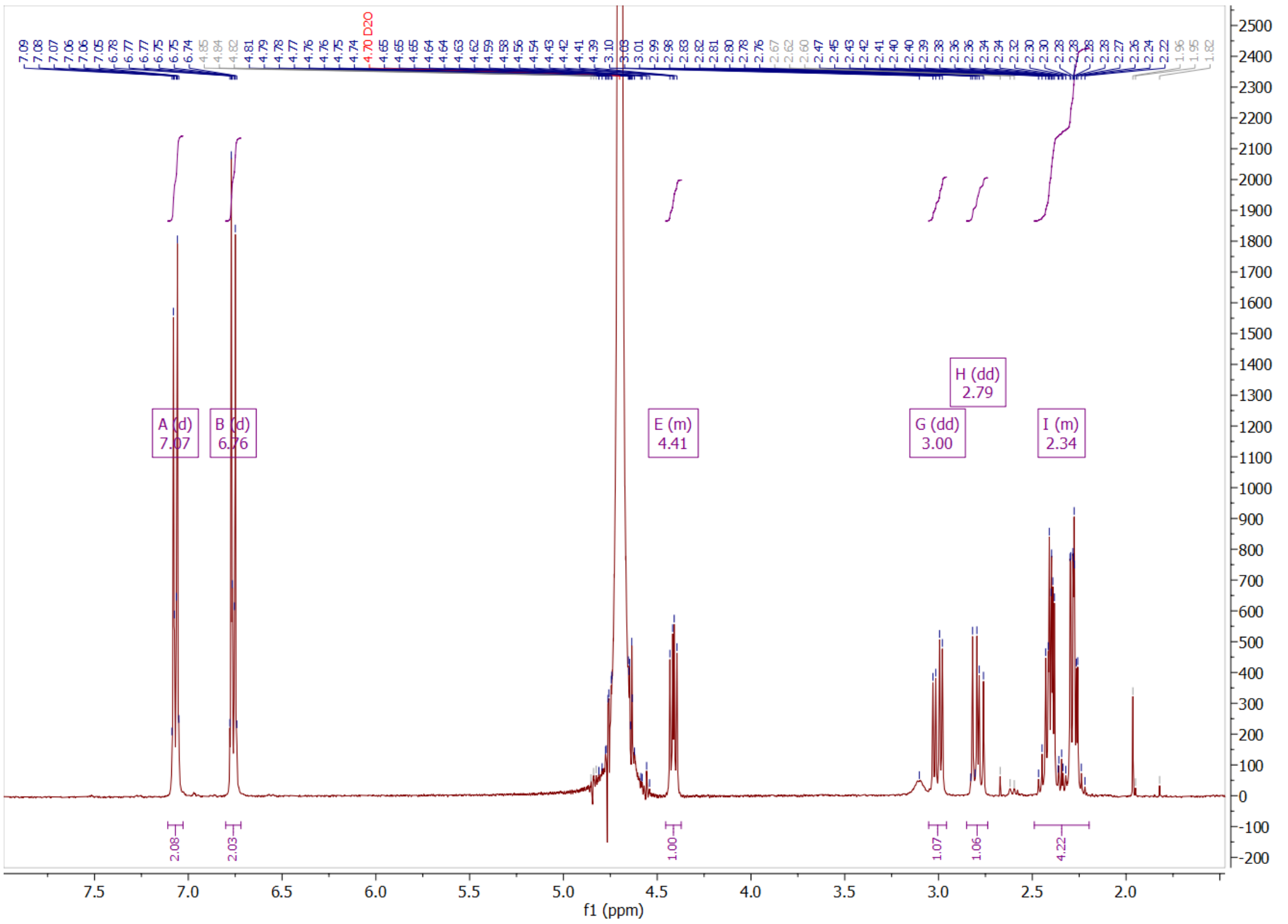

Compound **1-Hy**: ^{13}C NMR (101 MHz, D_2O)

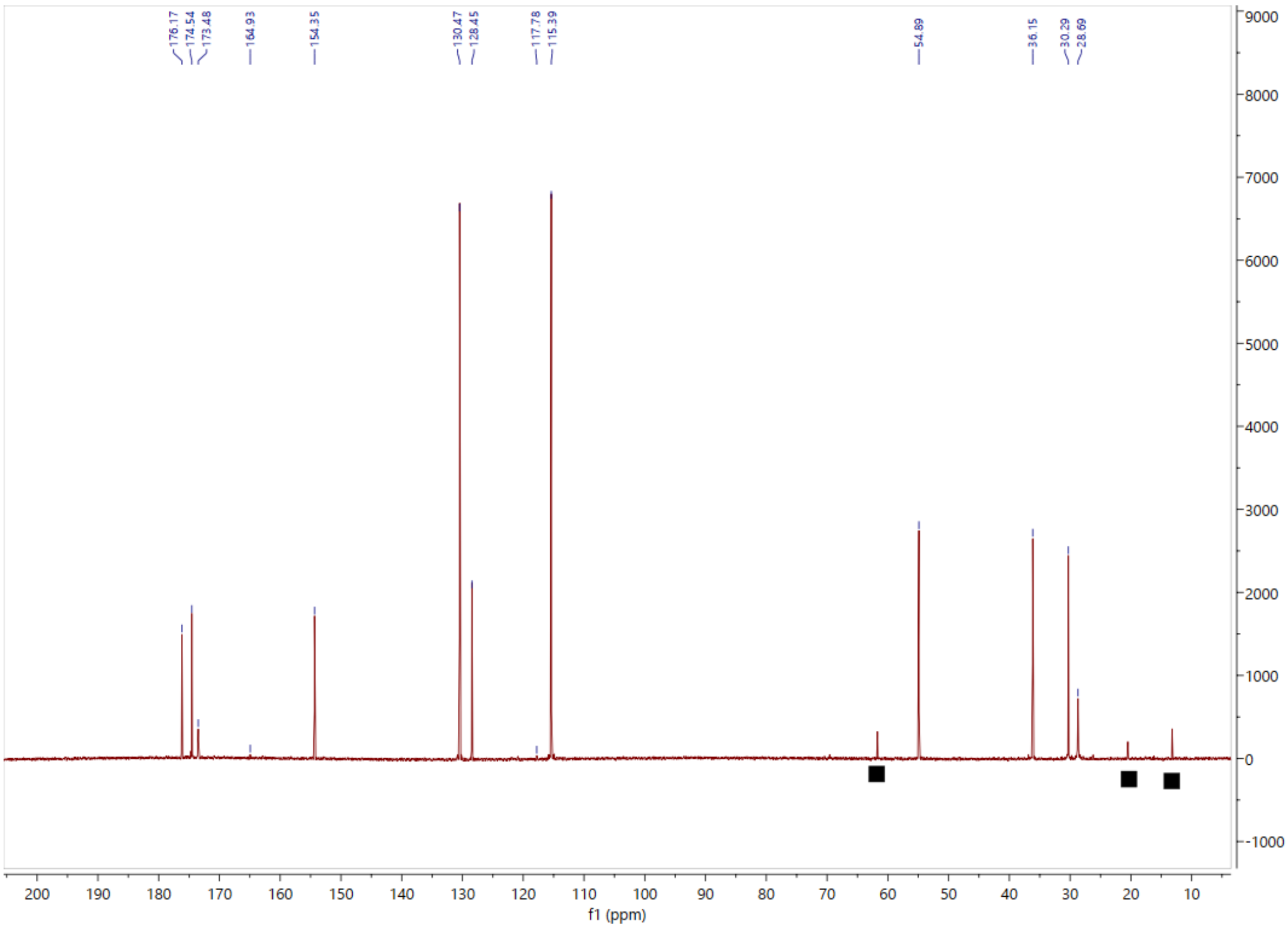

Note. Under the acquisition conditions used, the signals of the trifluoroacetate counterion, at δ = 164.93 and 117.78 ppm, have very low intensities. Black squares indicated unidentified impurities signals.

Compound **1-Hy**: ^{19}F NMR (376 MHz, D_2O)

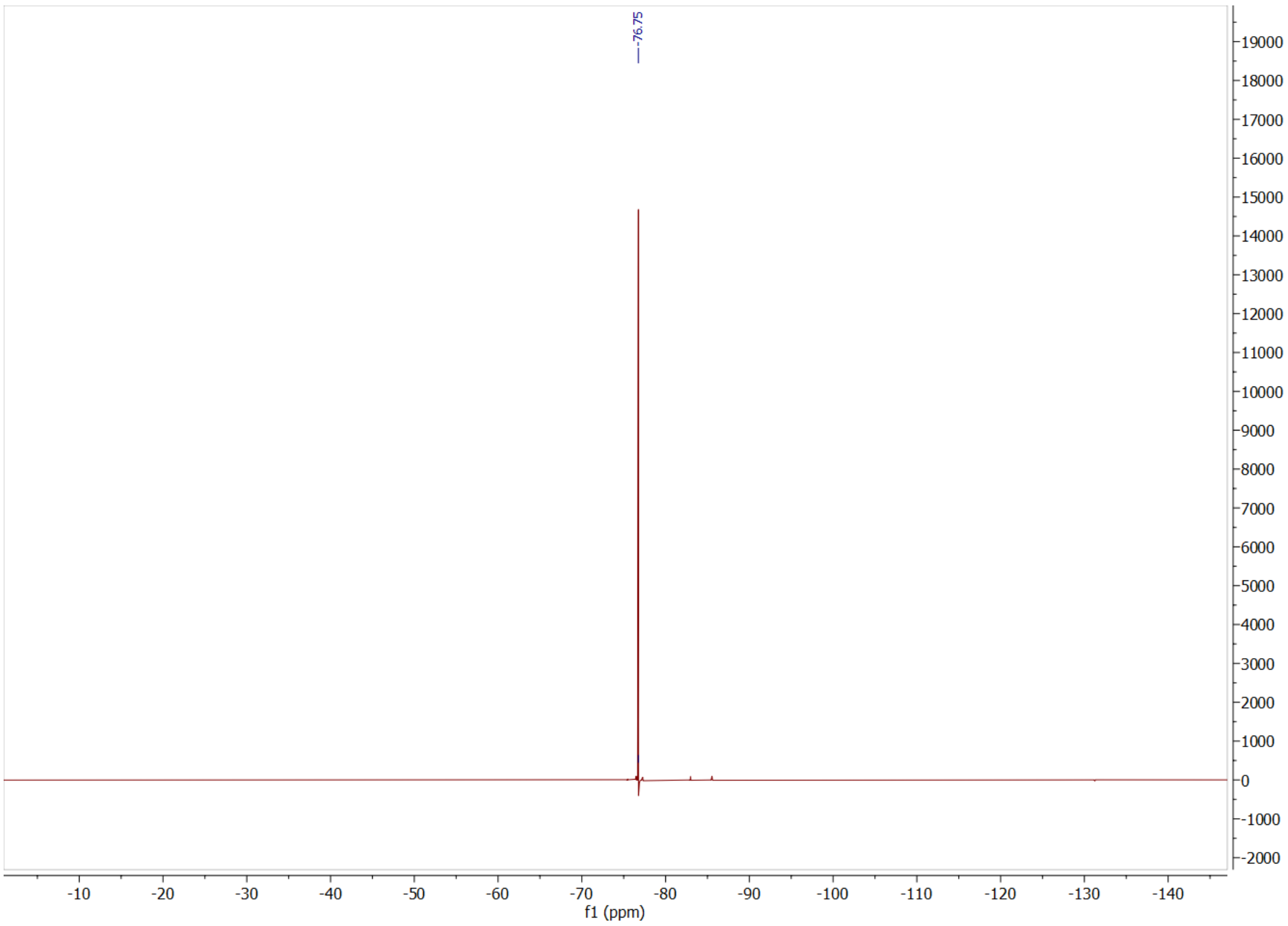

Compound **1-Hy**: UV-Vis absorption spectrum extracted from the HPLC chromatogram of the pure product

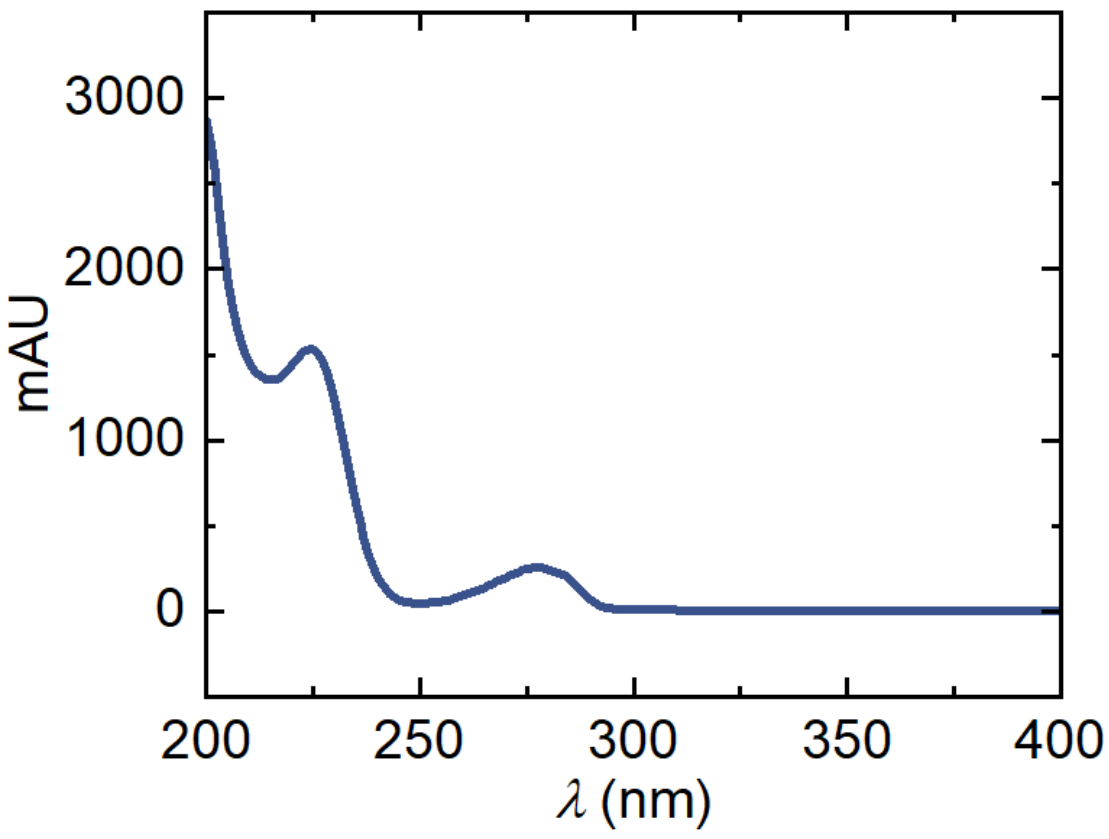

Compound **3**: ^{1}H NMR (400 MHz, DMSO-d_6)

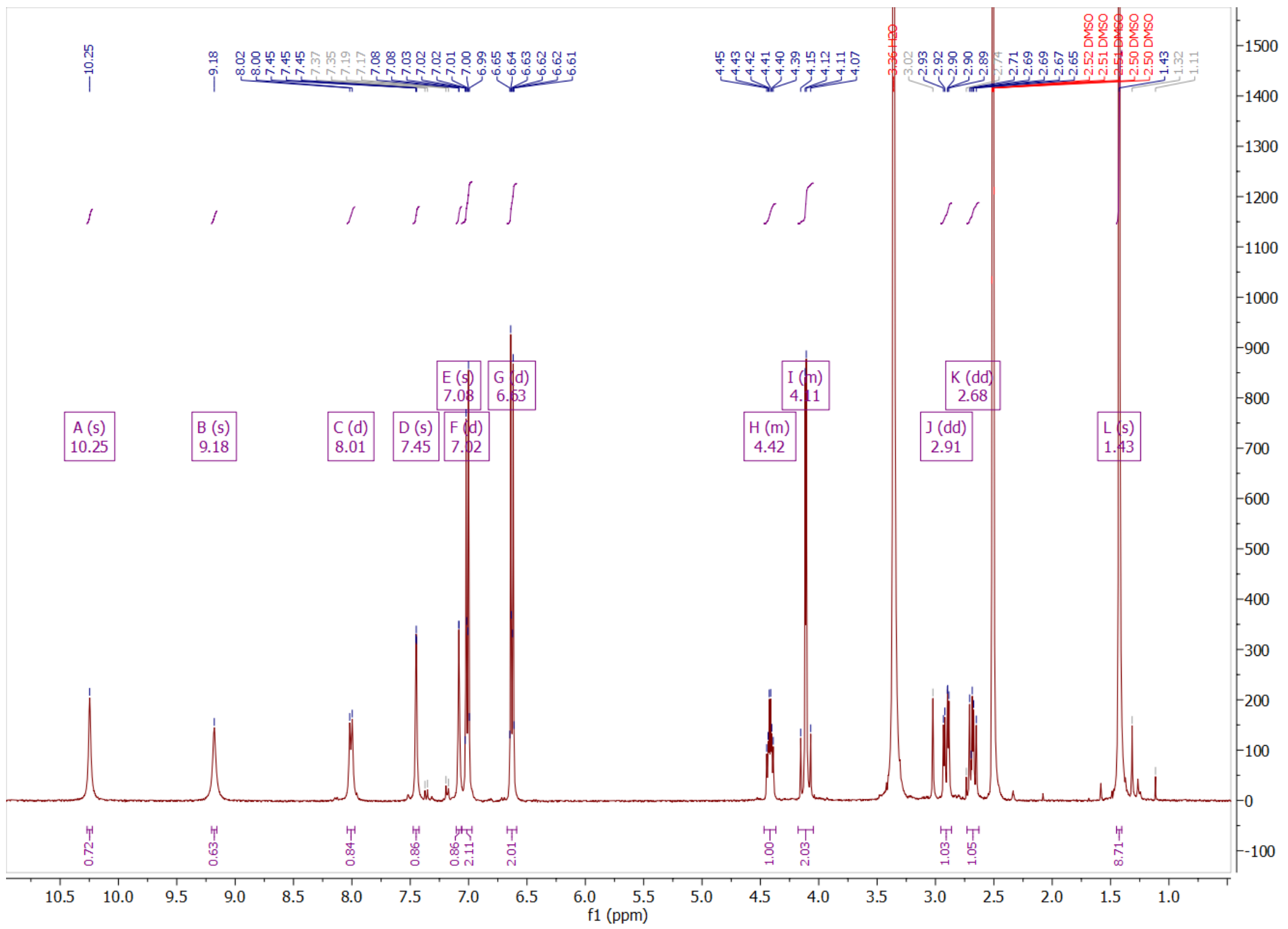

Compound **3**: ^{13}C NMR (75 MHz, DMSO-d_6)

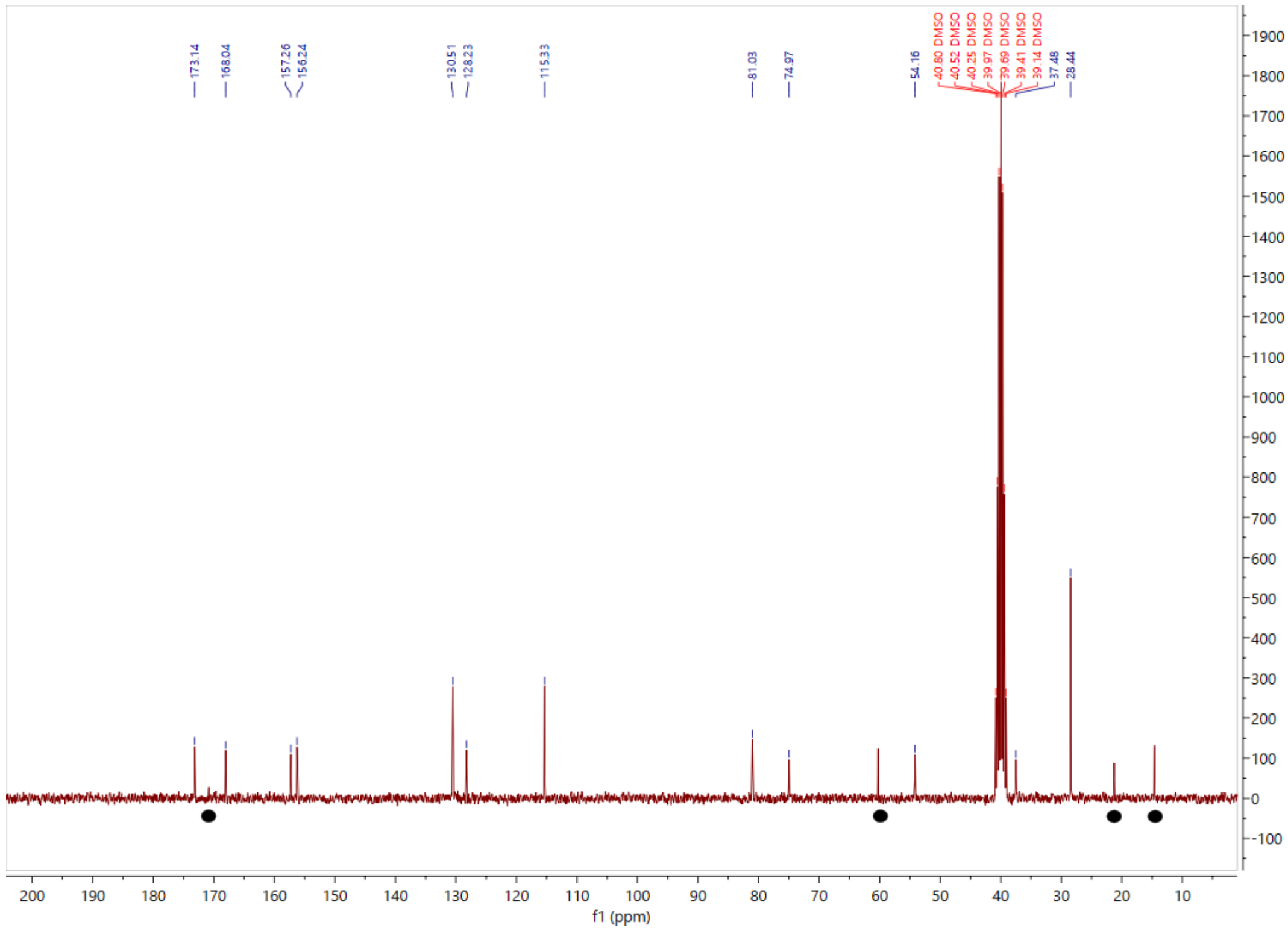

Note. Black dots indicate the signals of EtOAc

Compound **3-Al**: ^{1}H NMR (400 MHz, D_2O)

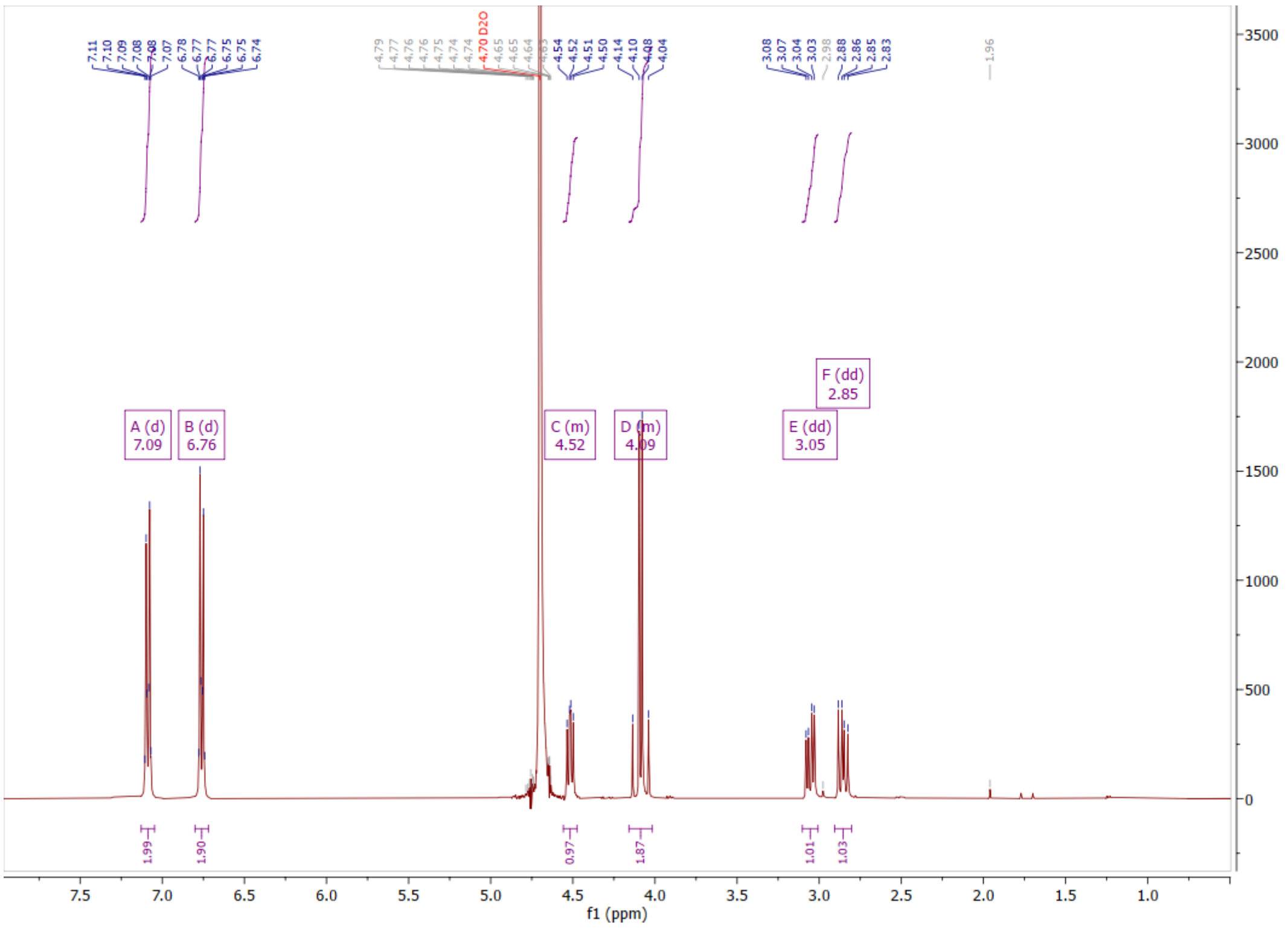

Compound **3-Al**: ^{13}C NMR (101 MHz, D_2O)

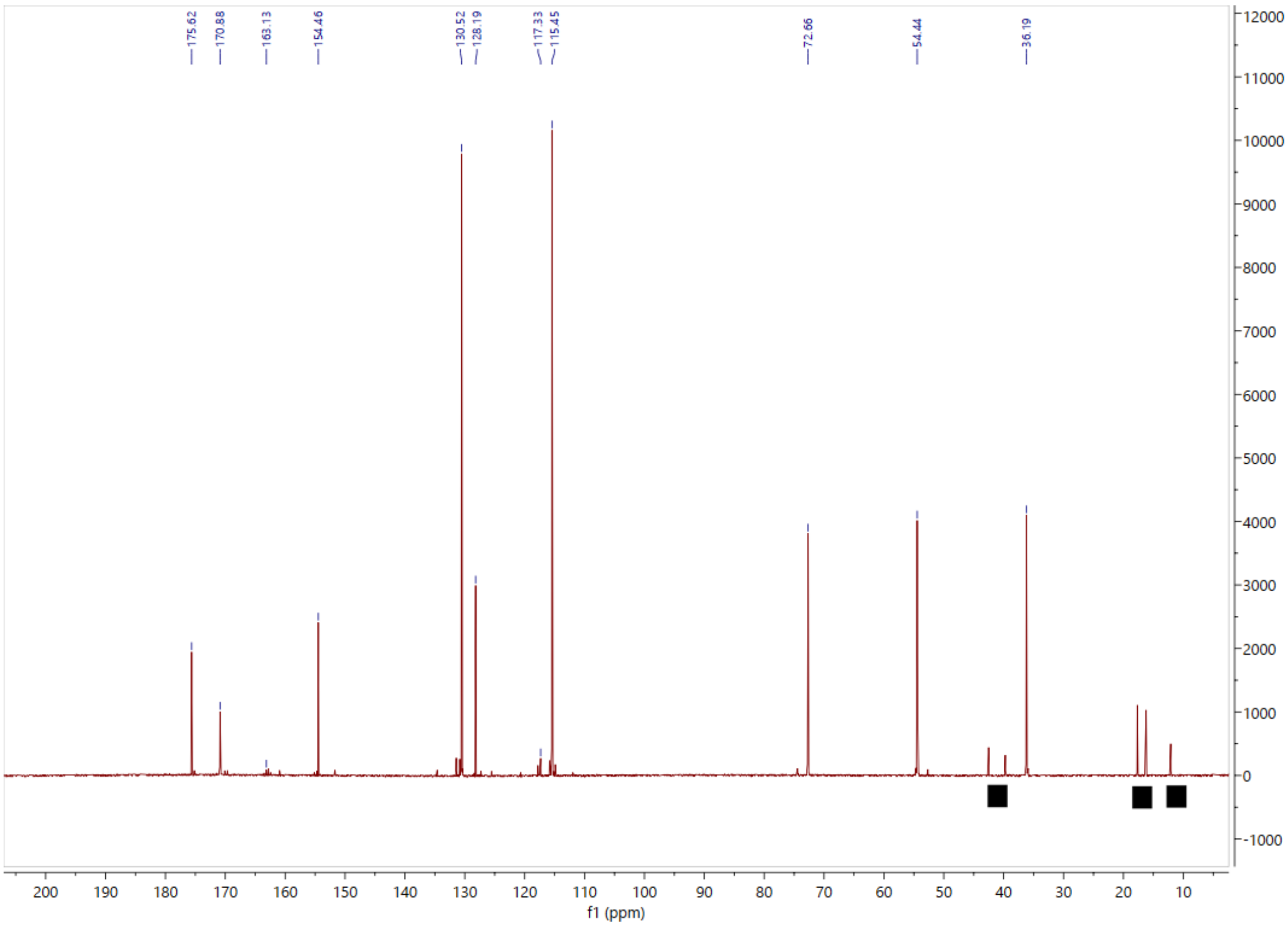

Note. Under the acquisition conditions used, the signals of the trifluoroacetate counterion, at δ = 163.13 and 117.33 ppm, have very low intensities. Black squares indicated unidentified impurities signals.

Compound **3-Al**: ^{19}F NMR (376 MHz, D_2O)

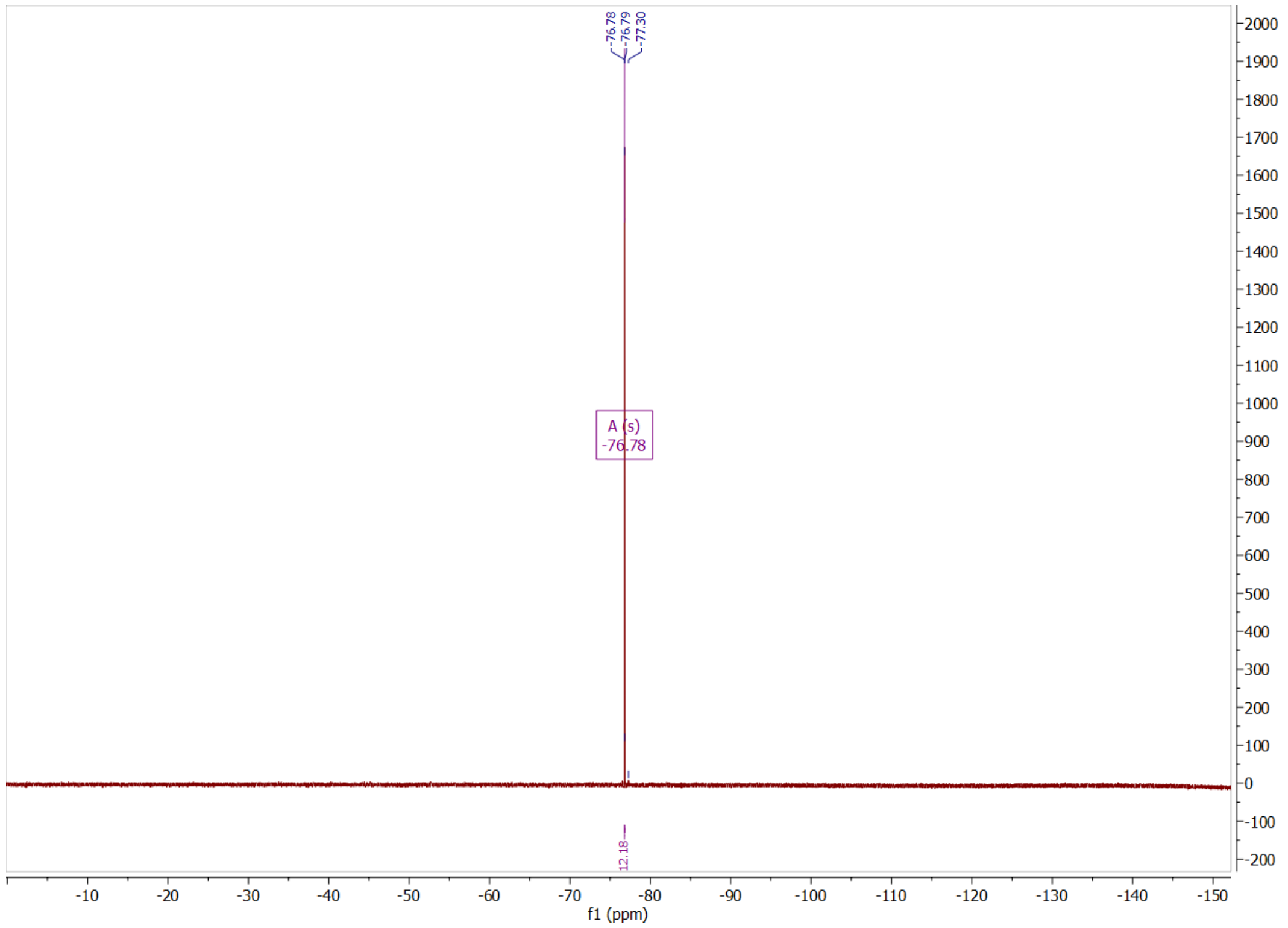

Compound **4**: ^{1}H NMR (300 MHz, DMSO-d_6)

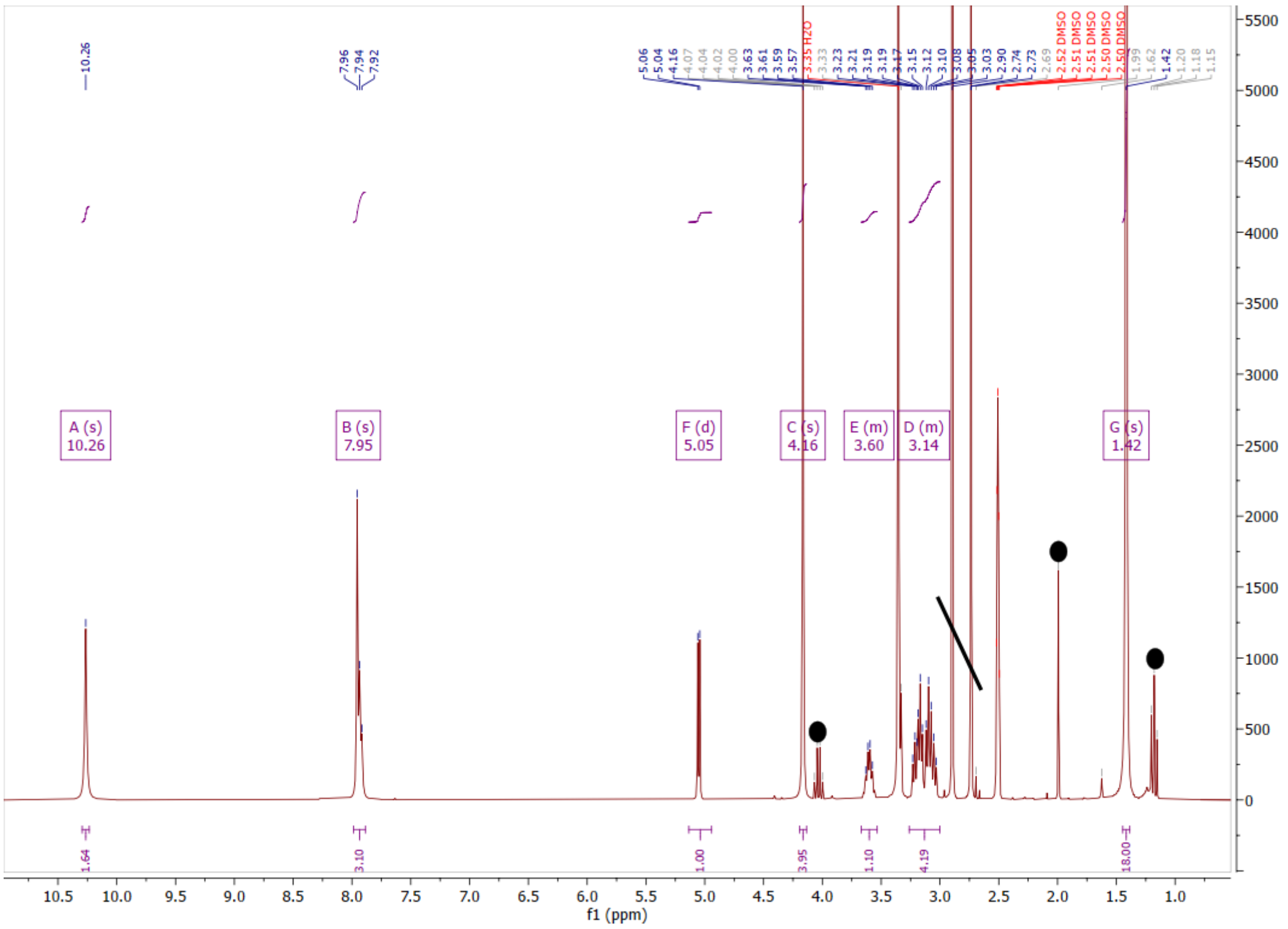

Note. Black dots indicate the signals of EtOAc. The black bar indicates unidentified impurities typically observed when using a specific batch of DMSO-d_6.

Compound **4**: ^{13}C NMR (75 MHz, DMSO-d_6)

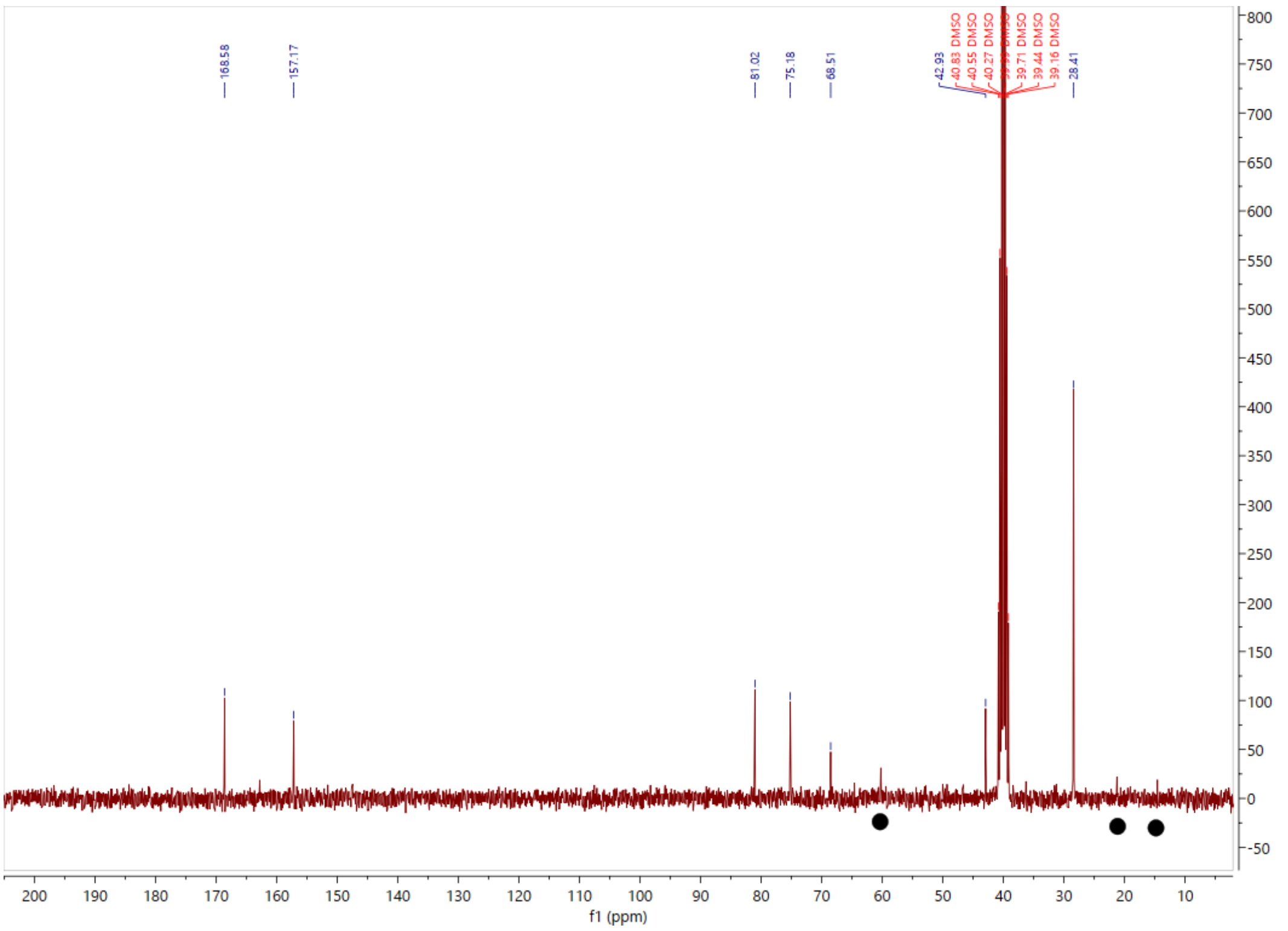

Note. Black dots indicate the signals of EtOAc.

Compound **4-Al**: ^{1}H NMR (300 MHz, DMSO-d_6)

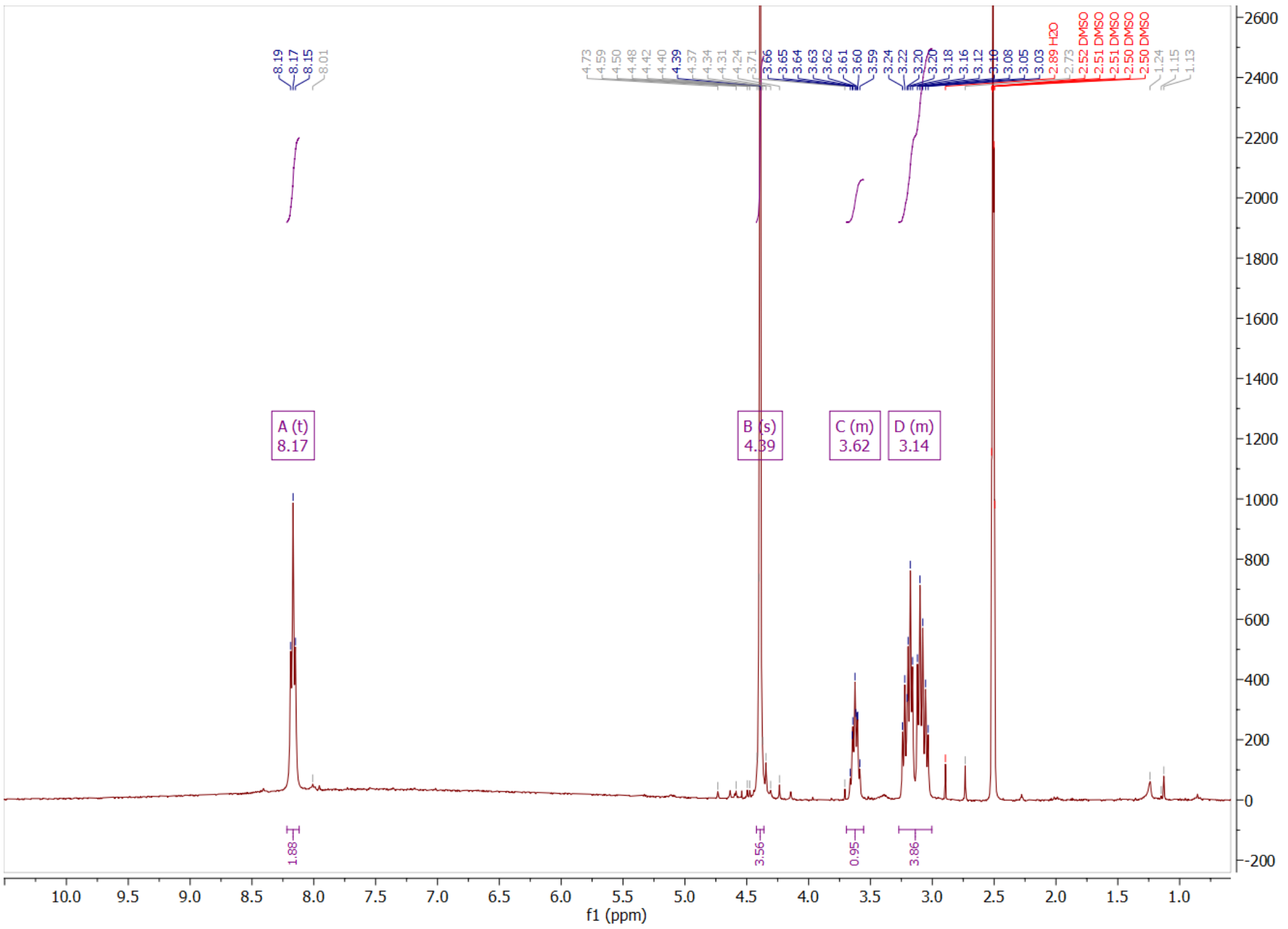

Compound **4-Al**: ^{13}C NMR (75 MHz, DMSO-d_6)

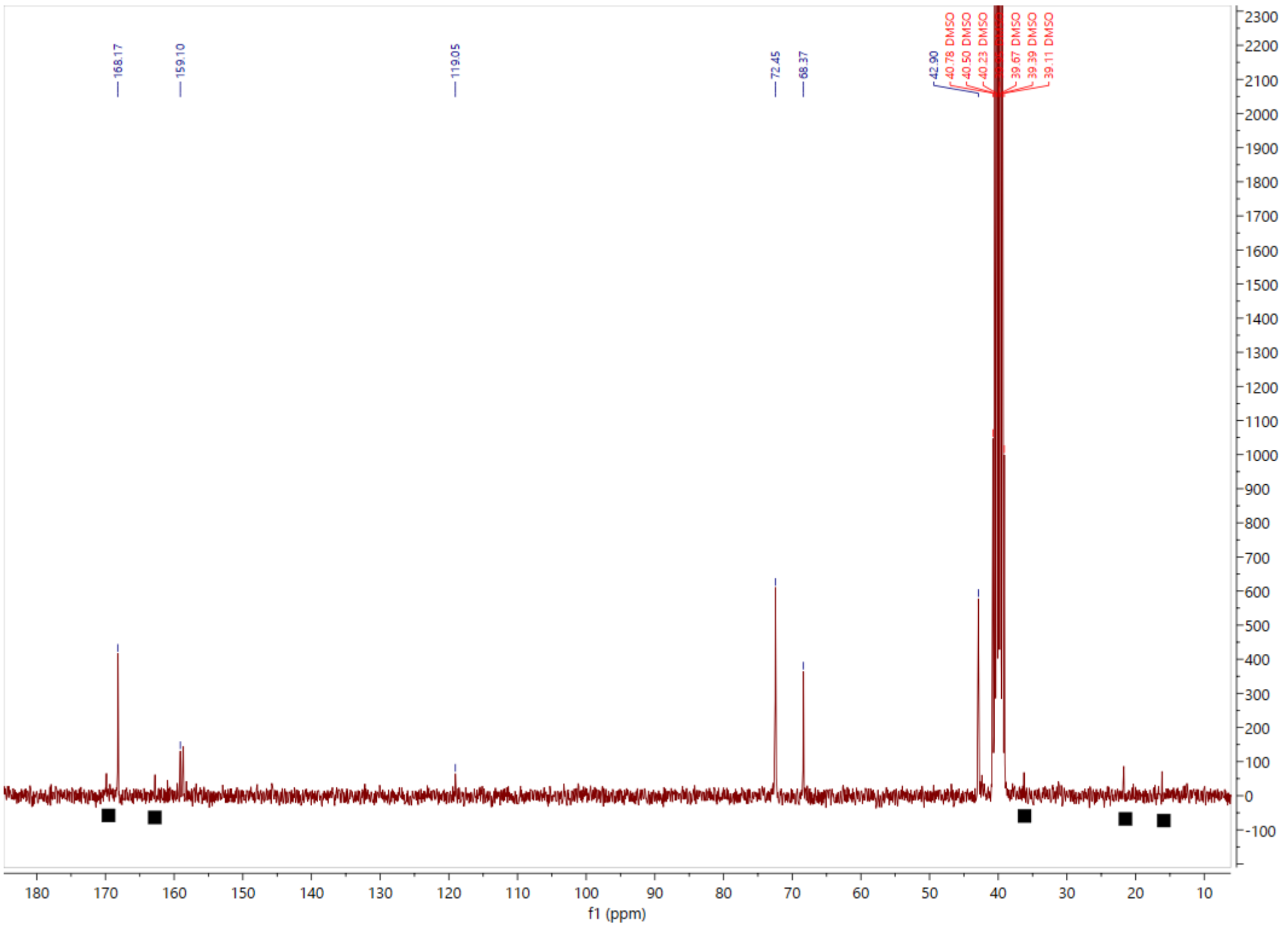

Note. The signals at δ = 159.10, 119.05 ppm are assigned to the trifluoroacetate counterion. Black squares indicate unidentified impurities signals.

Compound **4-Al**: ^{19}F NMR (376 MHz, D_2O)

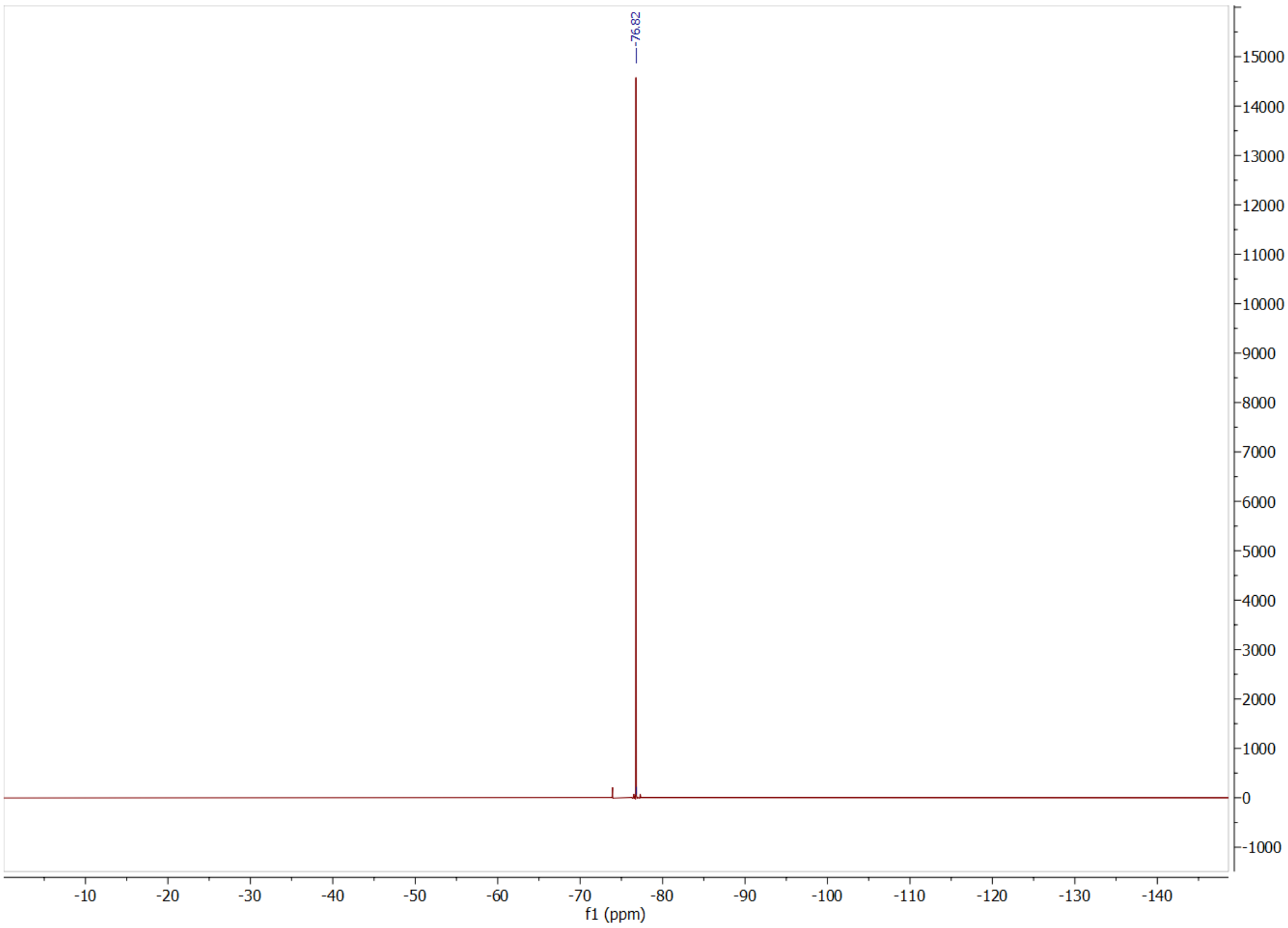

Compound **4-Al**: ^{19}F NMR (282 MHz, DMSO-d_6)

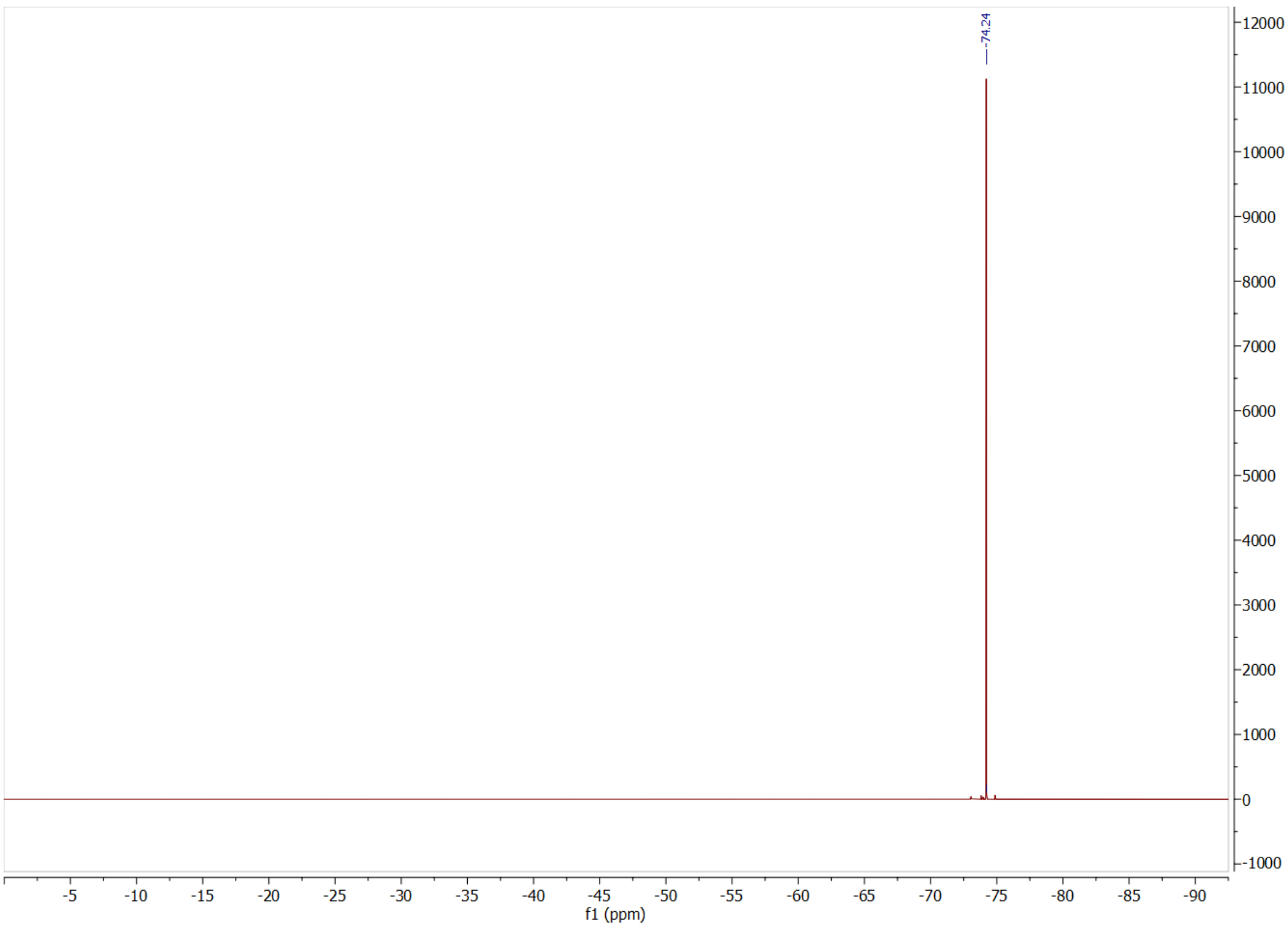

Compound **5**: ^{1}H NMR (400 MHz, DMSO-d_6)

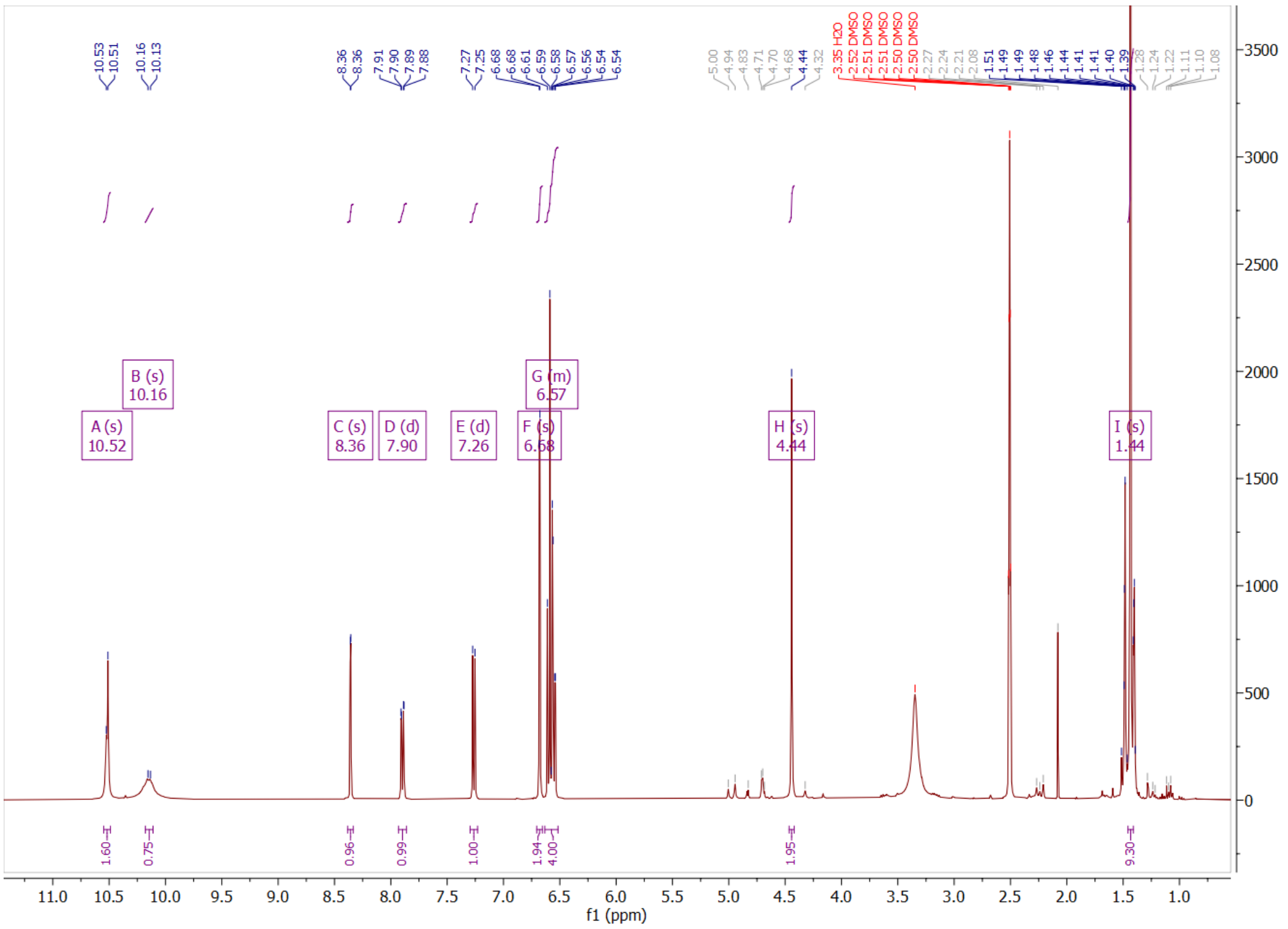

Note. The small impurities visible particularly between ca. 5.0 and 4.6 ppm are unidentified. However, the compound served only as intermediate to afford 5-Al which was purified by preparative HPLC.

Compound **5**: ^{13}C NMR (101 MHz, DMSO-d_6)

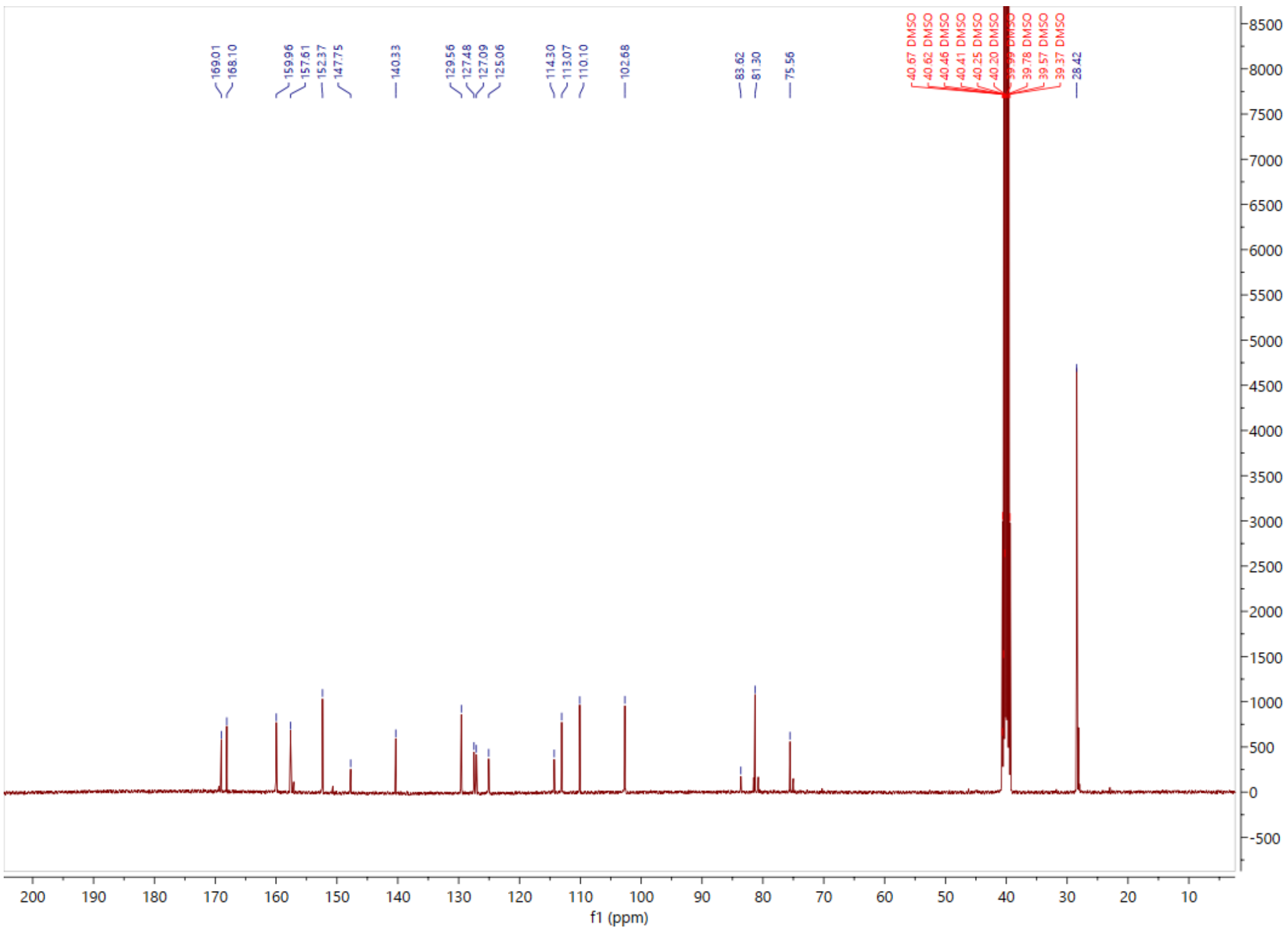

Compound **5**: ESI$^+$ HRMS

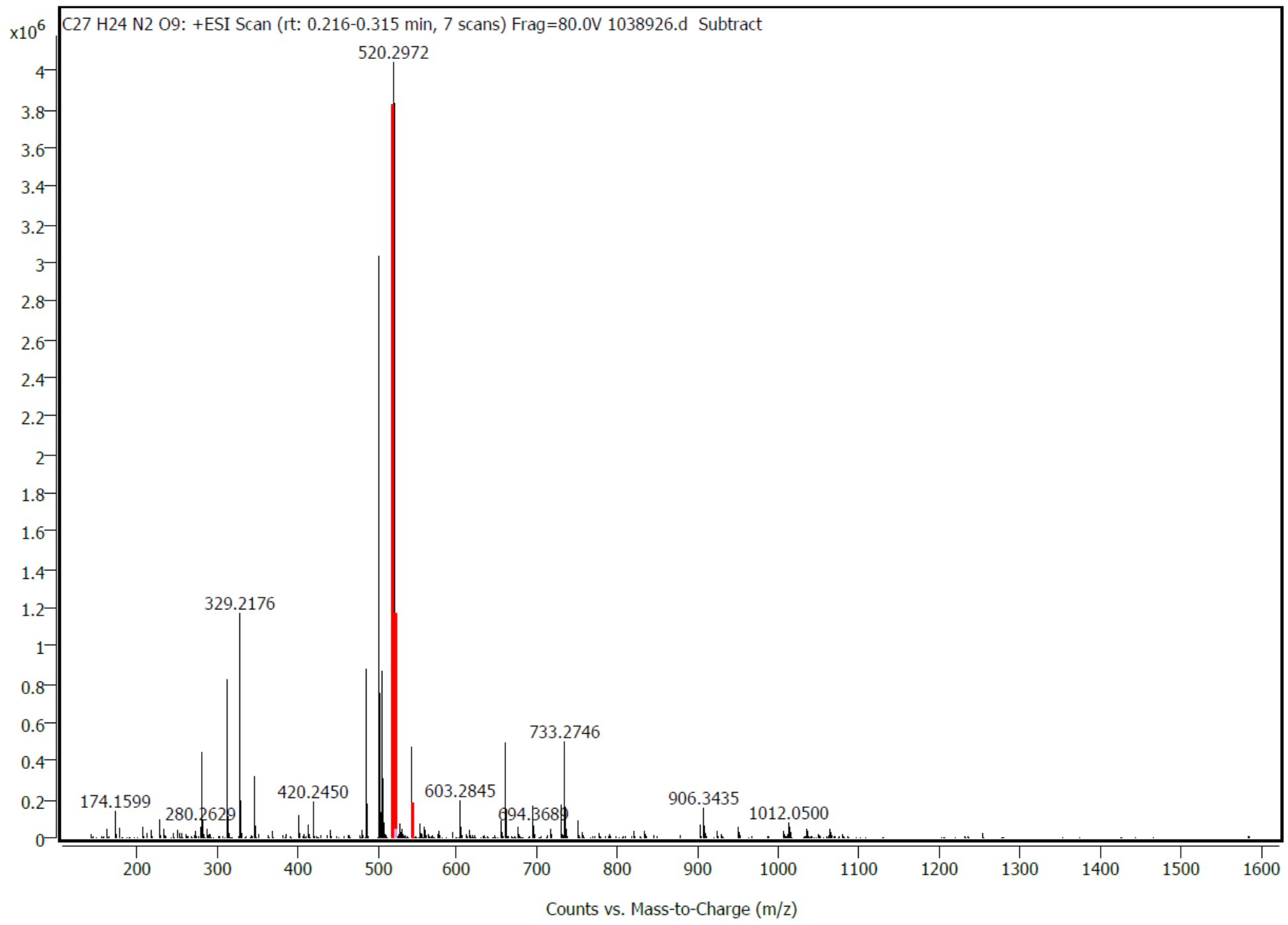

Compound **5-Al**: ^{1}H NMR (400 MHz, DMSO-d_6)

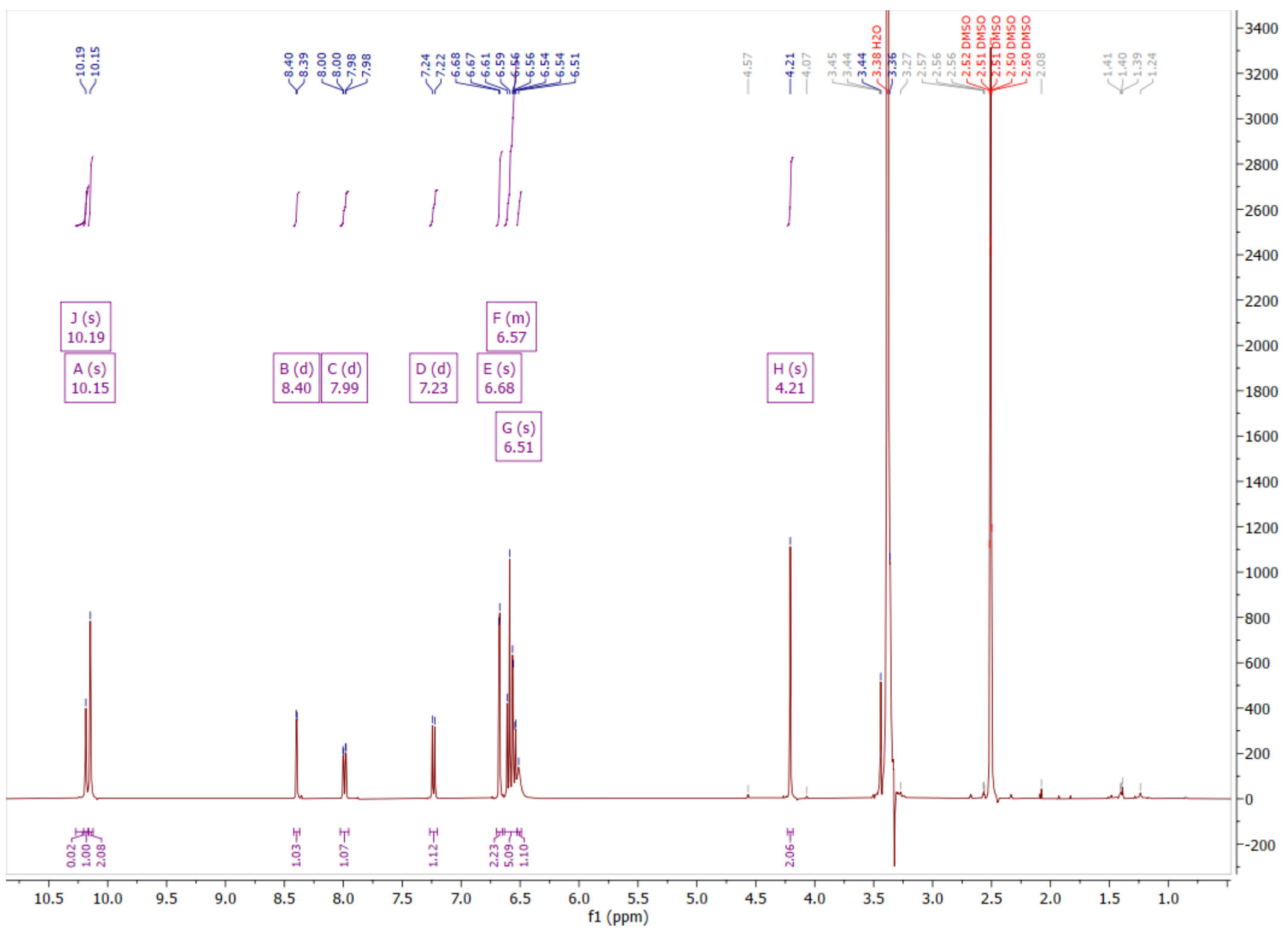

Compound **5-Al**: ^{13}C NMR (101 MHz, DMSO-d_6)

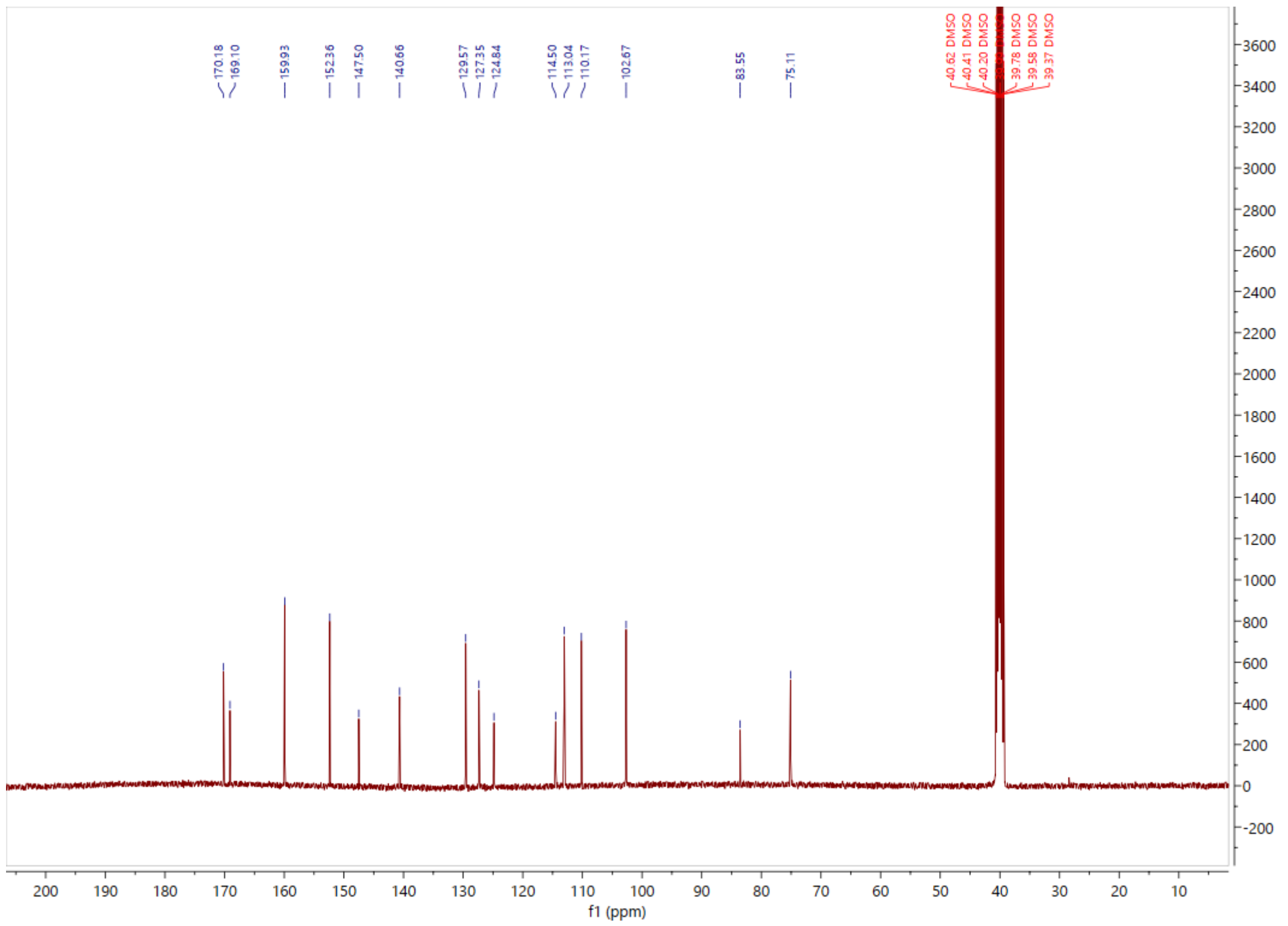

Compound **5-Al**: ^{19}F NMR (376 MHz, D_2O)

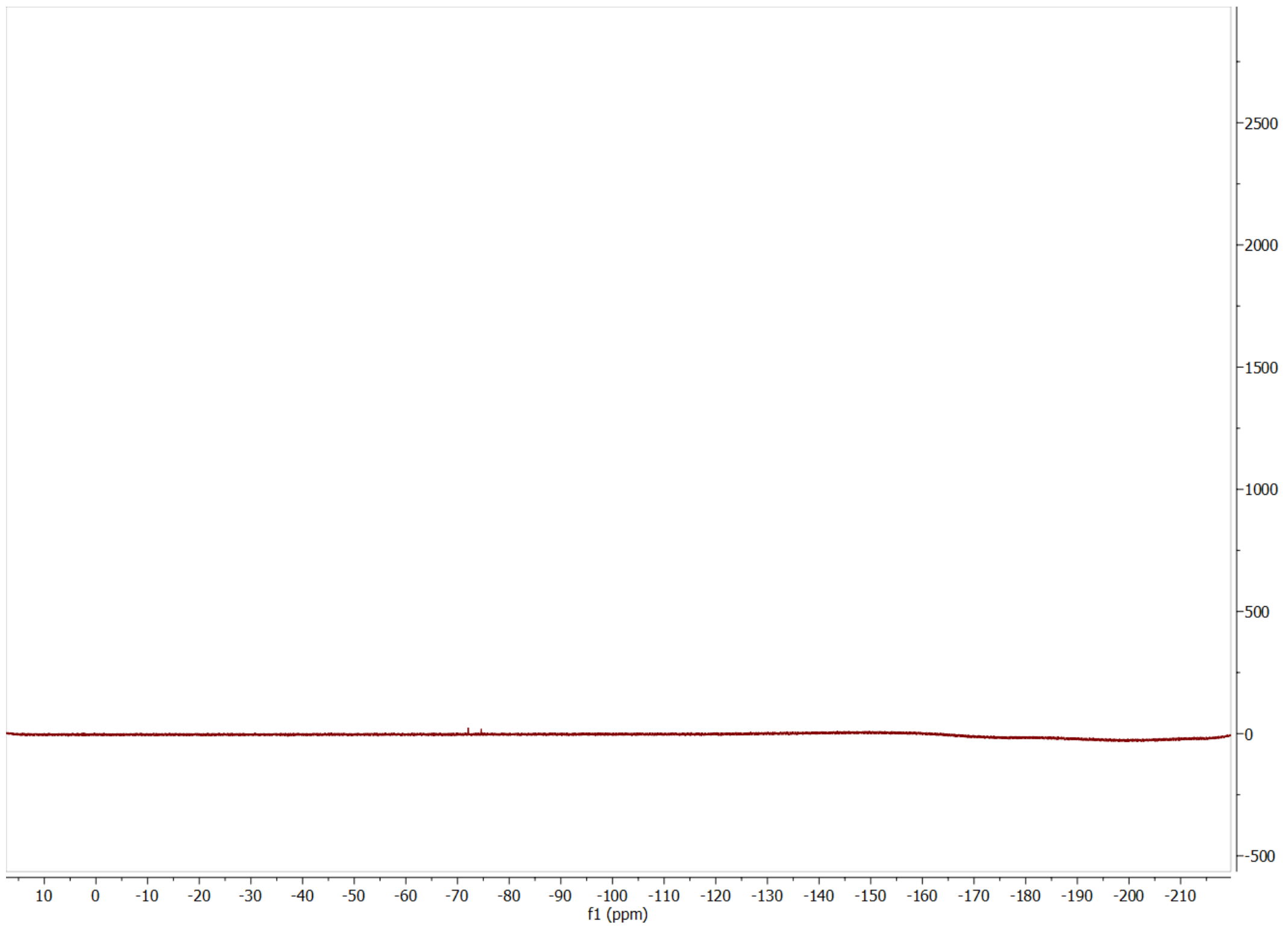

Compound **5-Al**: ESI$^+$ HRMS

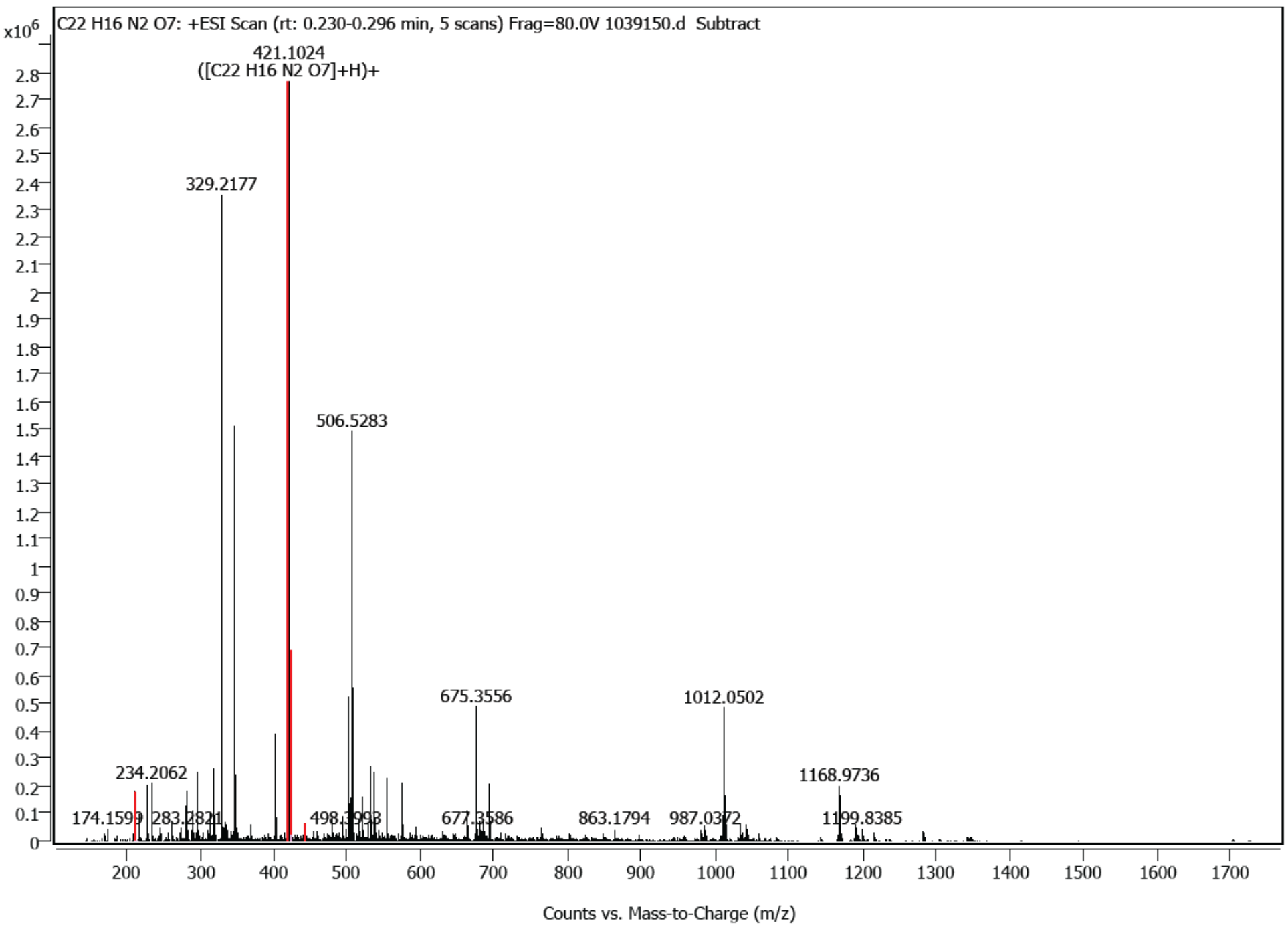

Compound **6**: ^{1}H NMR (300 MHz, DMSO-d_6)

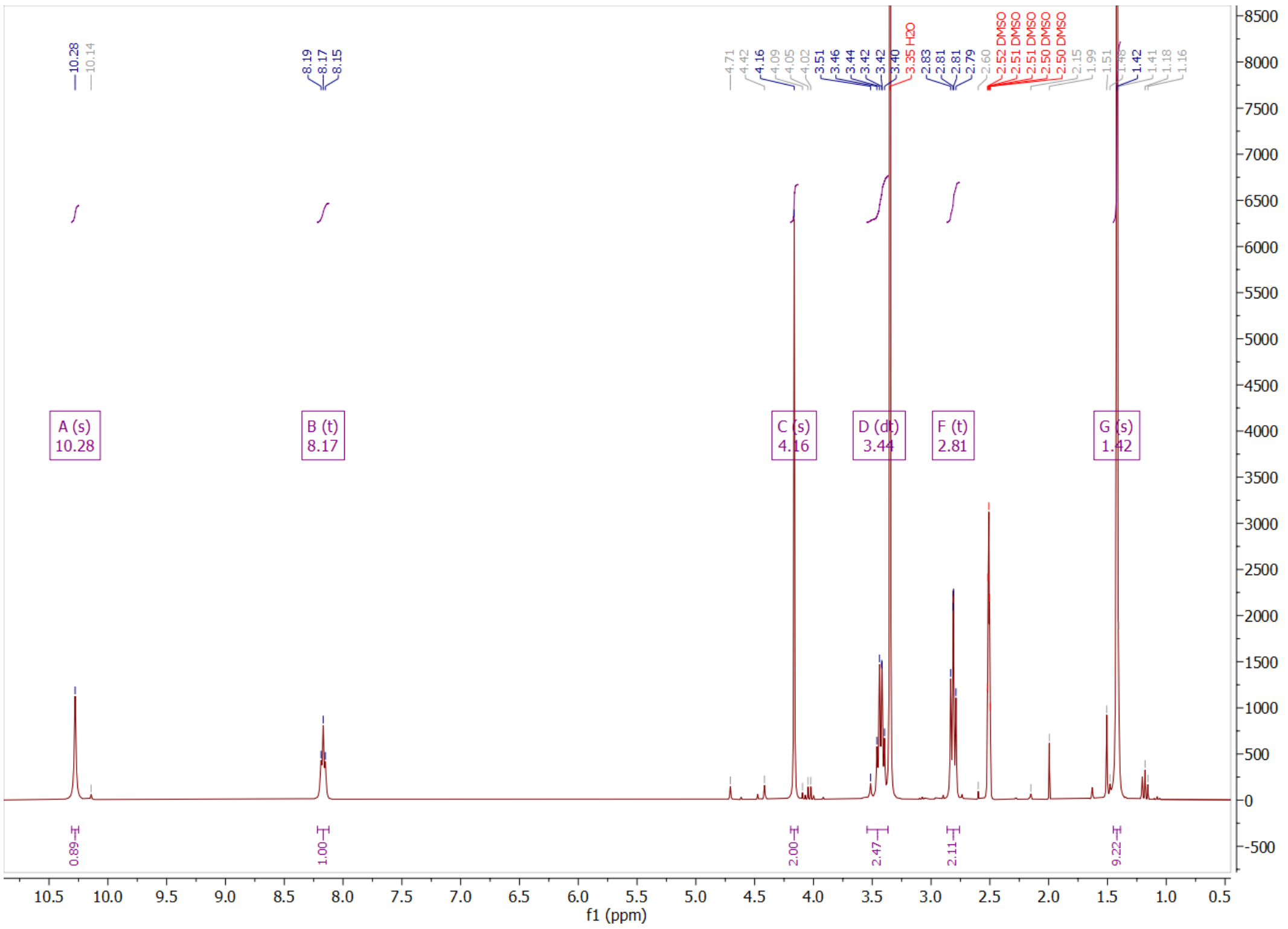

Compound **6**: ^{13}C NMR (75 MHz, DMSO-d_6)

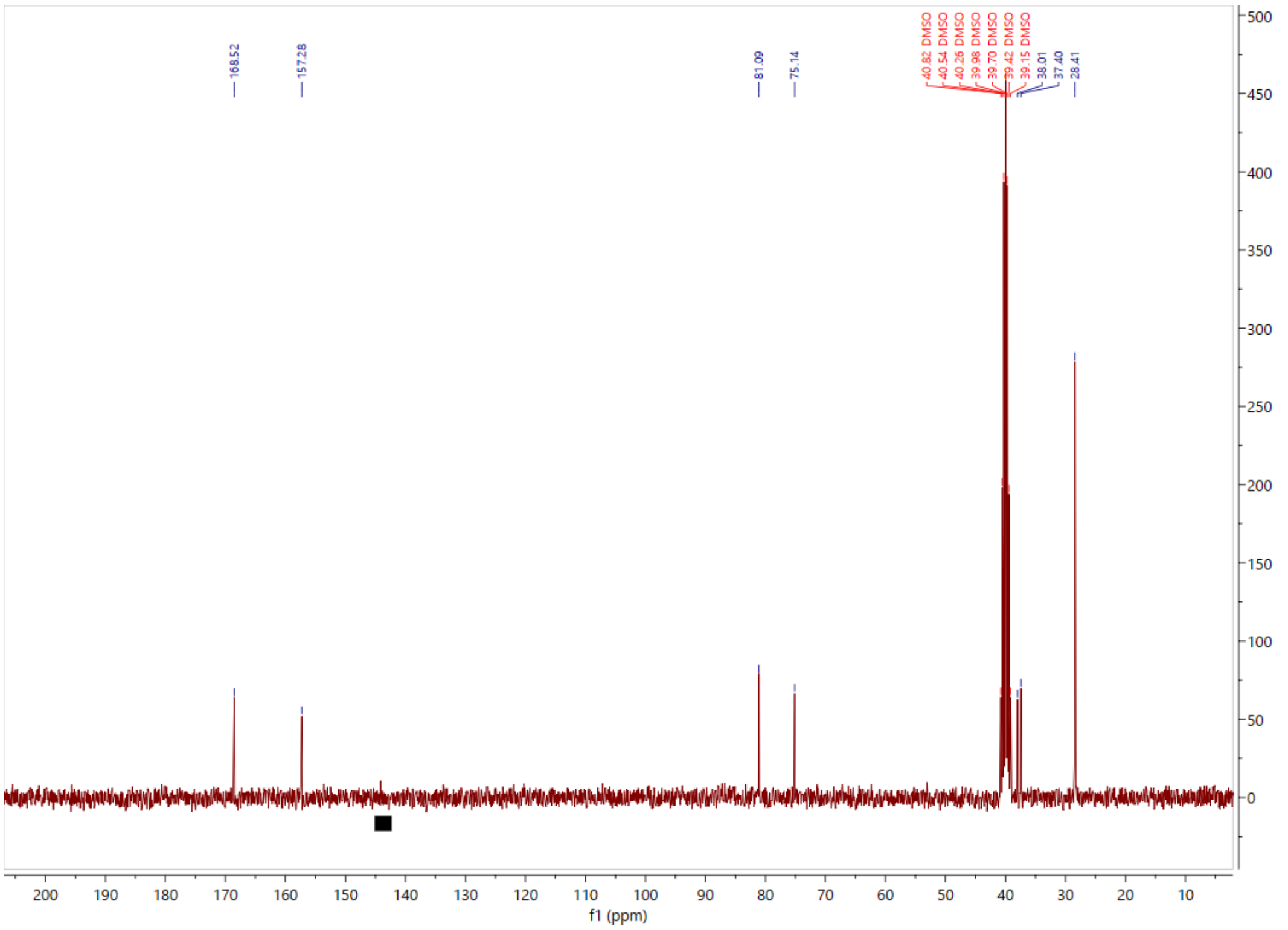

Note. Black square indicates unidentified impurity signal.

Compound **6-Al**: ^{1}H NMR (400 MHz, D_2O)

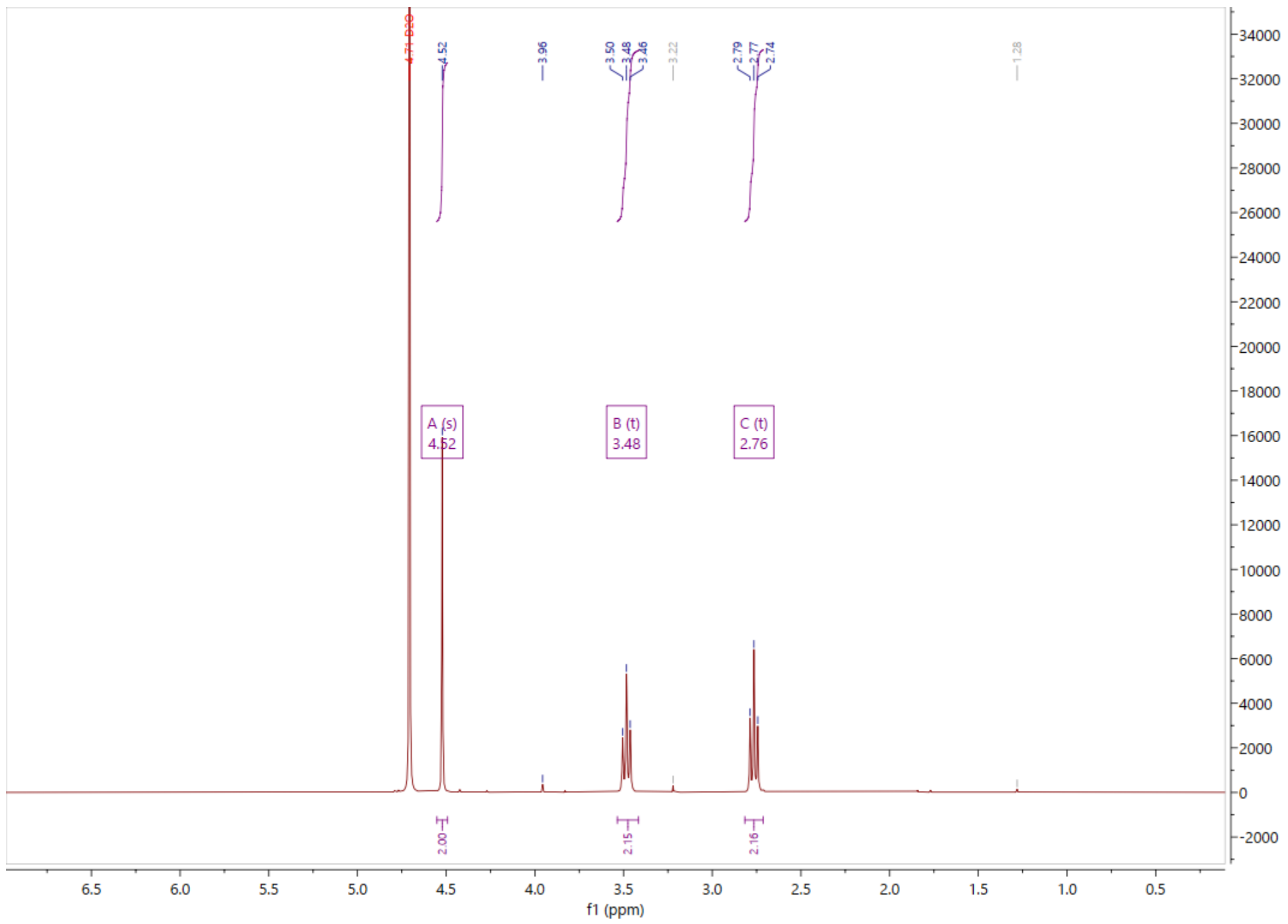

Compound **6-Al**: ^{13}C NMR (101 MHz, D_2O)

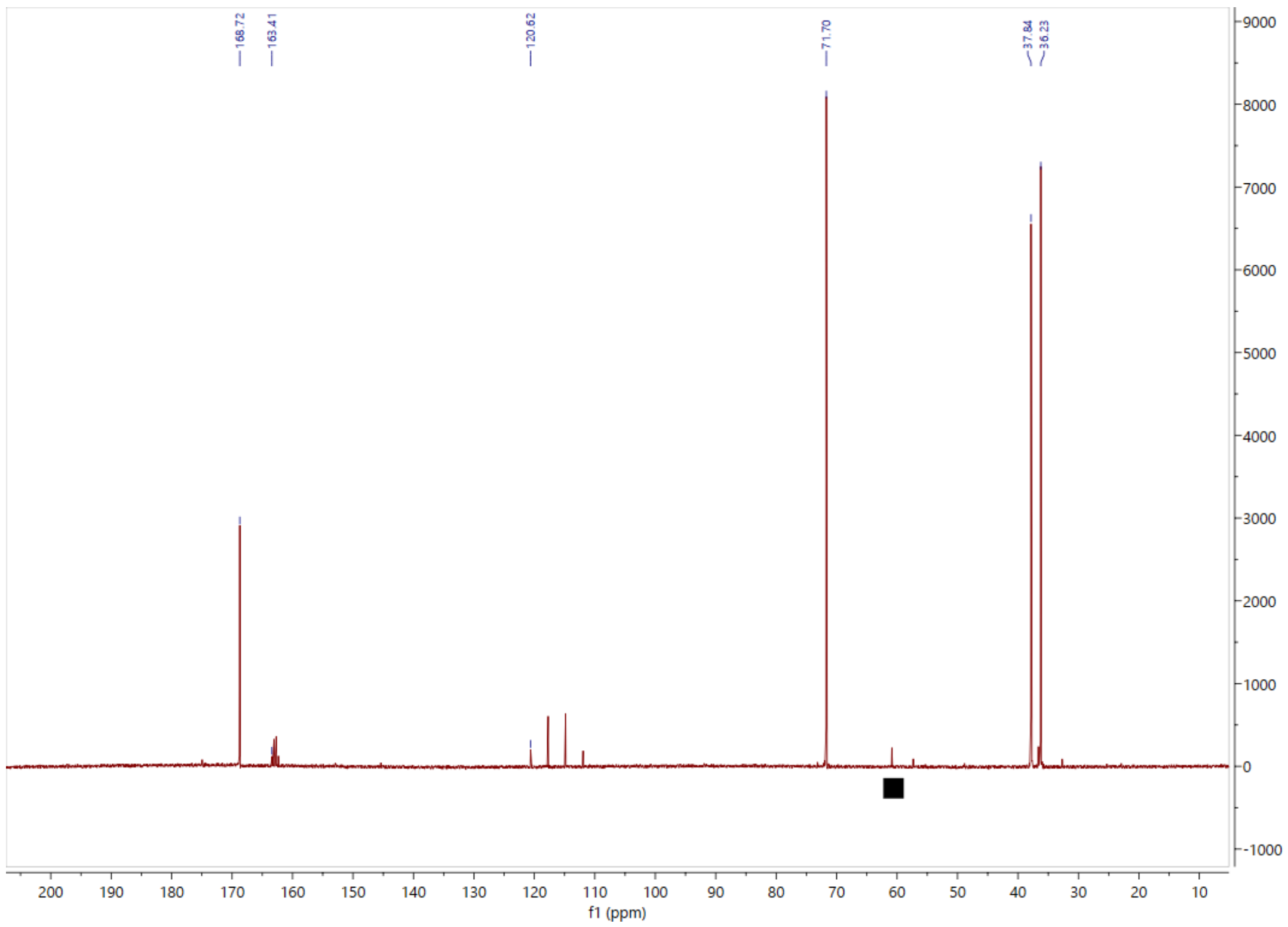

Note. The quartet signals at δ = 163.41 and 120.62 ppm are assigned to, respectively, the carbonyl and aliphatic carbons of the trifluoroacetate counterion. Black square indicates unidentified impurity signal.

Compound **6-Al**: ^{19}F NMR (376 MHz, D_2O)

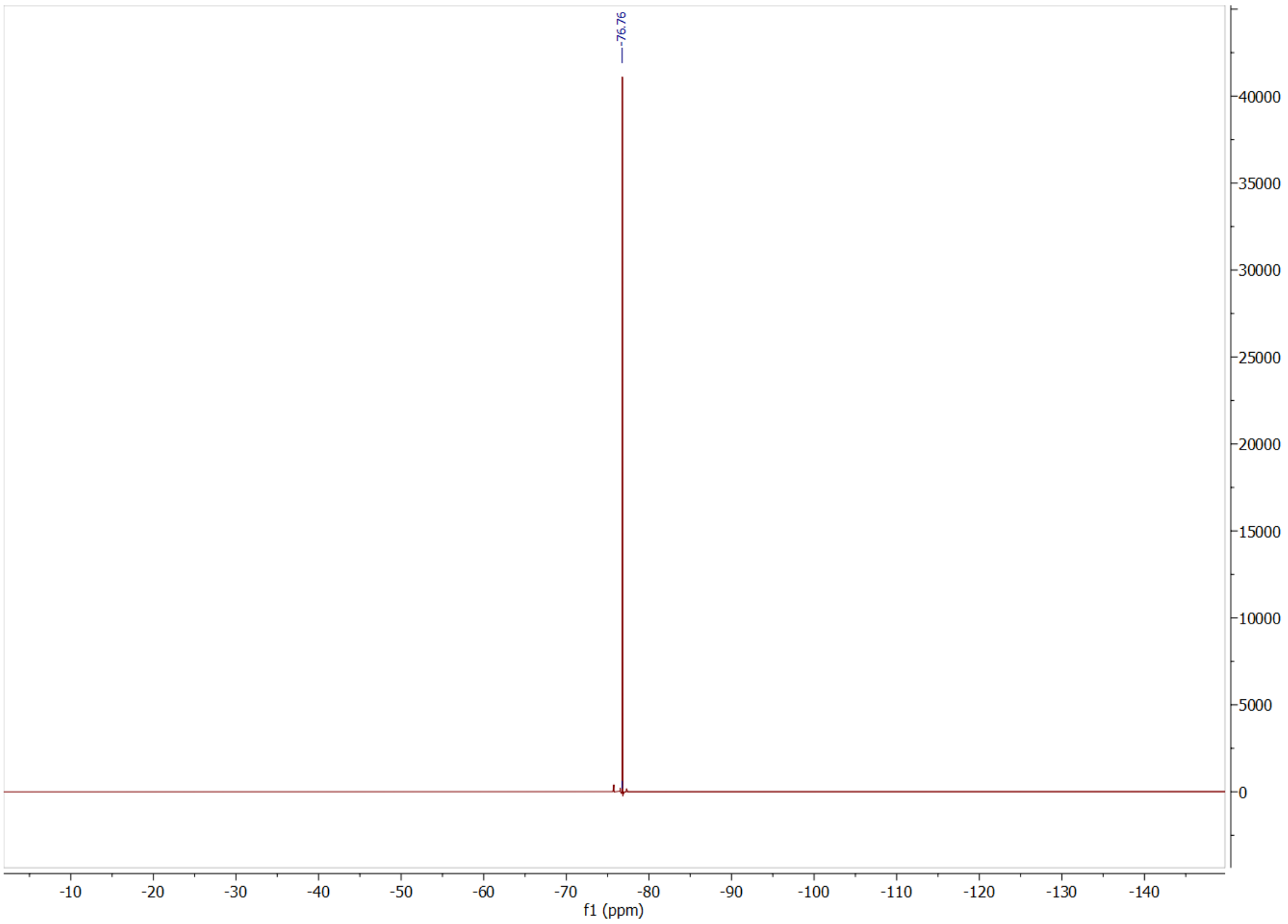

Compound **7**: ^{1}H NMR (300 MHz, DMSO-d_6)

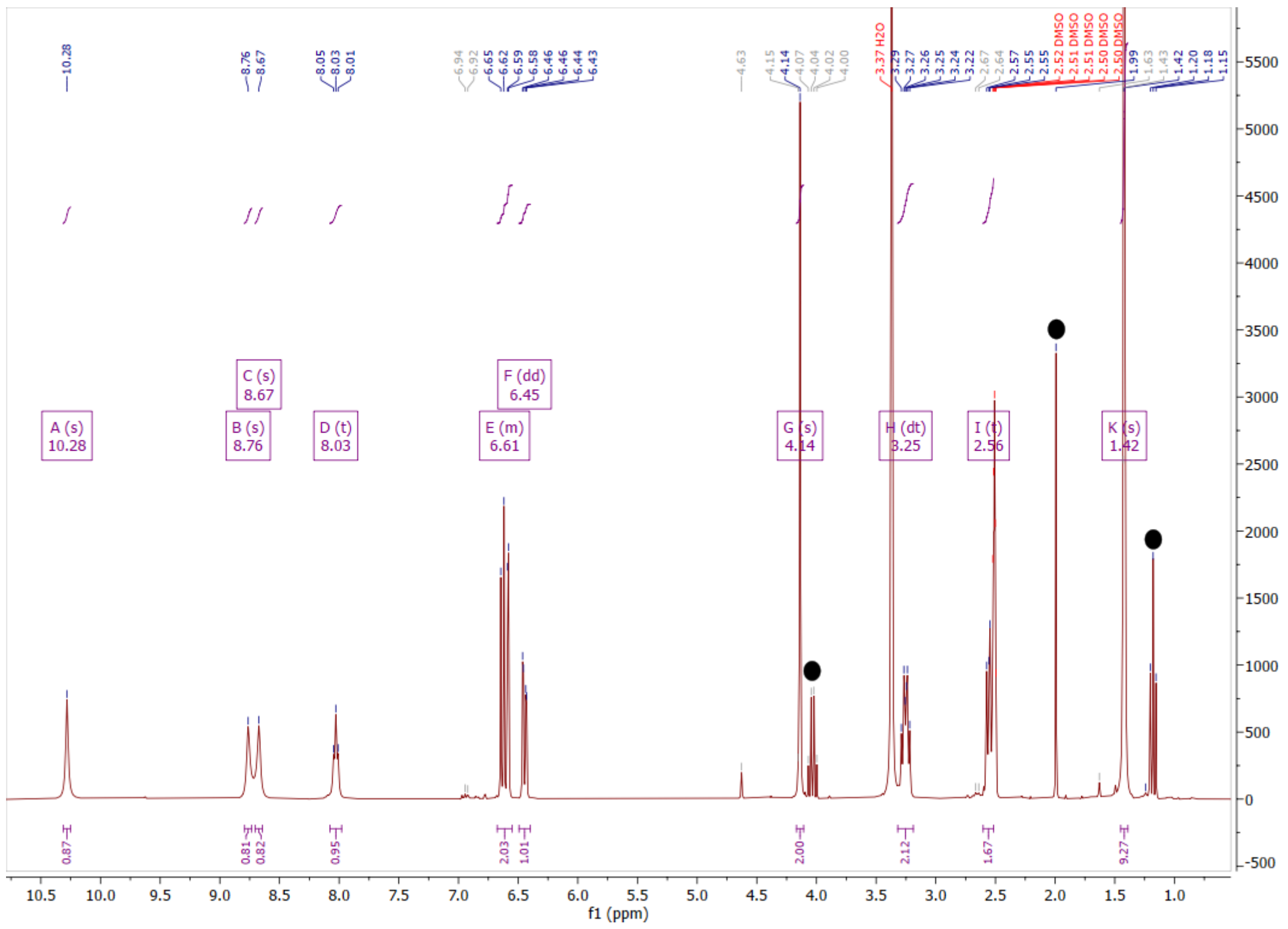

Note. Black dots indicate the signals of EtOAc.

Compound **7**: ^{13}C NMR (75 MHz, DMSO-d_6)

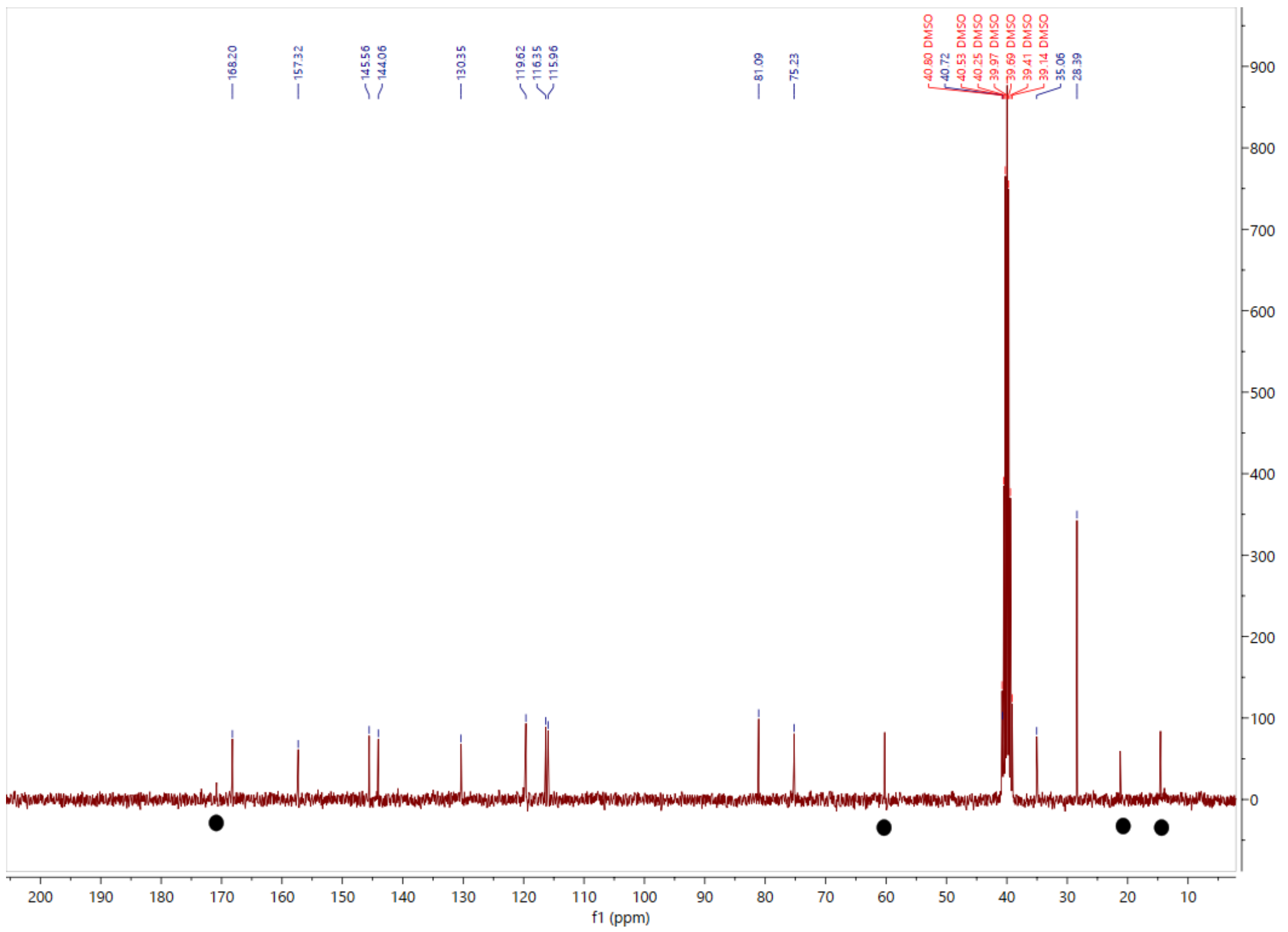

Note. Black dots indicate the signals of EtOAc. One compound signal falls under the signal of DMSO.

Compound **8**: ^{1}H NMR (300 MHz, D_2O)

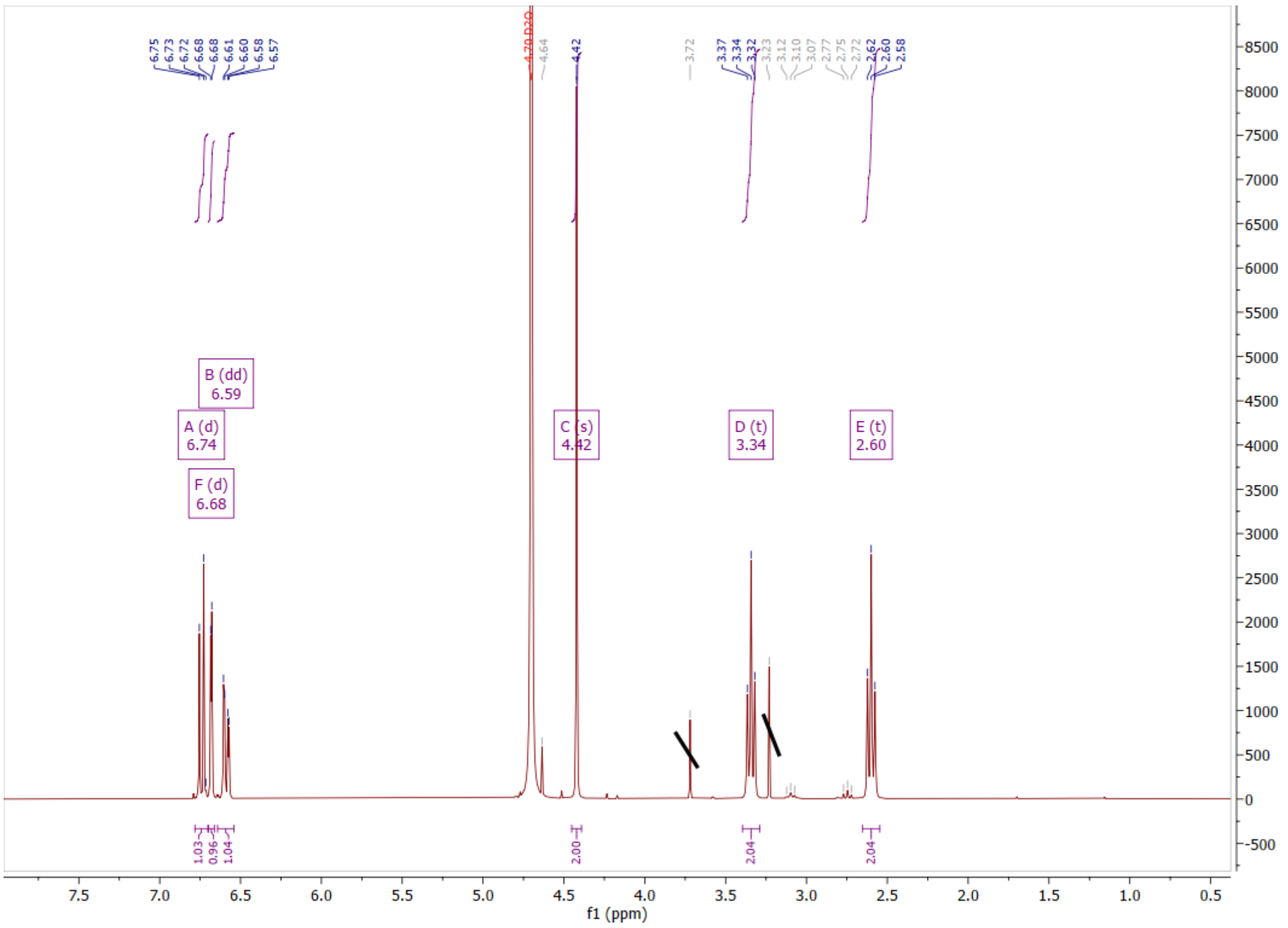

Note. Black bars indicate minor unidentified impurities signals.

Compound **8**: ^{13}C NMR (75 MHz, D_2O)

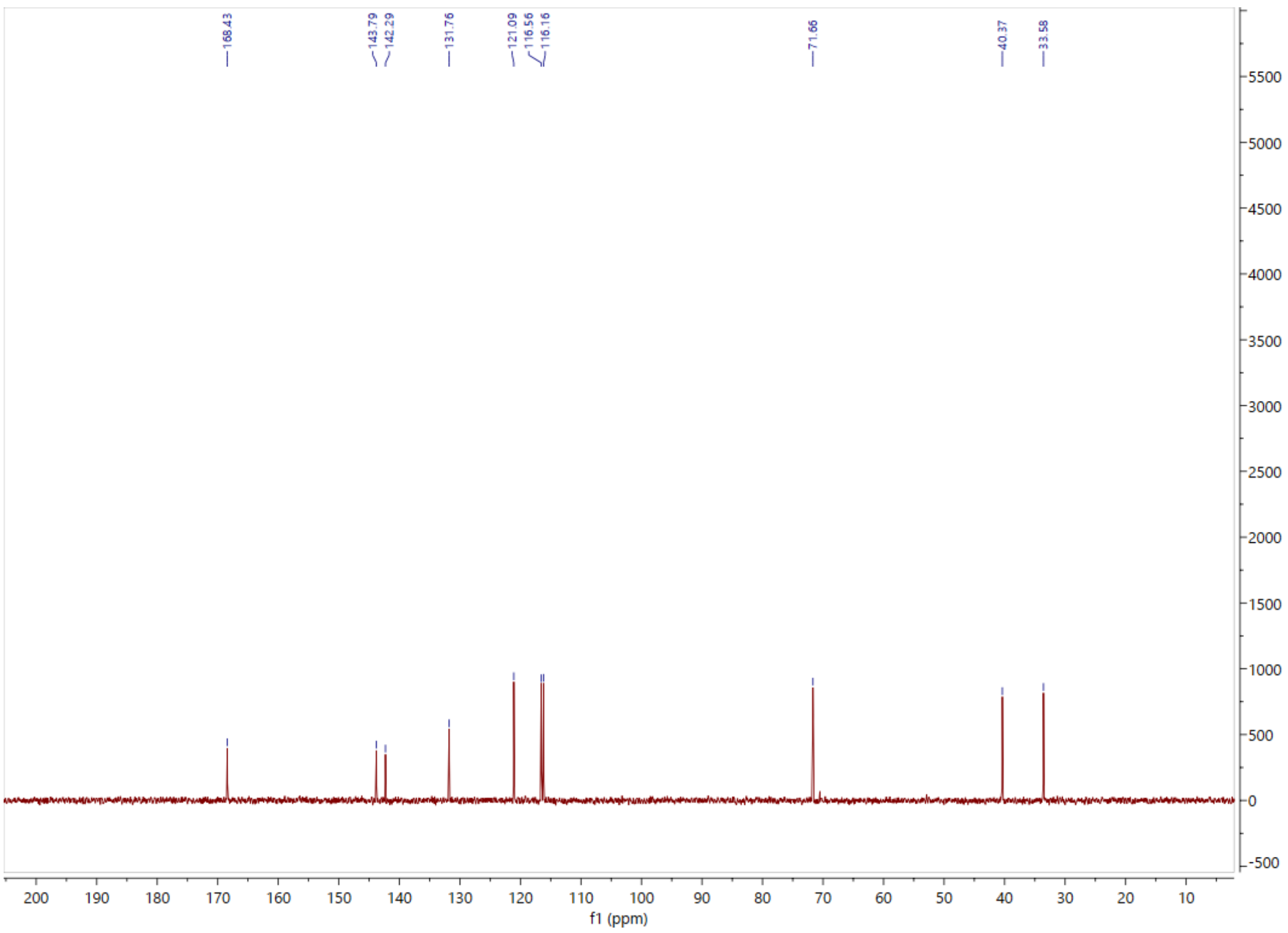

Supplementary References

1. Dirksen, A., Dirksen, S., Hackeng, T. M. & Dawson, P. E. Nucleophilic Catalysis of Hydrazone Formation and Transimination: Implications for Dynamic Covalent Chemistry. *J. Am. Chem. Soc.* **128**, 15602–15603 (2006).
2. McKinnon, D. D., Domaille, D. W., Cha, J. N. & Anseth, K. S. Biophysically Defined and Cytocompatible Covalently Adaptable Networks as Viscoelastic 3D Cell Culture Systems. *Adv. Mater.* **26**, 865–872 (2014).
3. McKinnon, D. D., Domaille, D. W., Cha, J. N. & Anseth, K. S. Bis-Aliphatic Hydrazone-Linked Hydrogels Form Most Rapidly at Physiological pH: Identifying the Origin of Hydrogel Properties with Small Molecule Kinetic Studies. *Chem. Mater.* **26**, 2382–2387 (2014).
