# Supplementary material for: Post-fabrication reconfiguration of hydrogels through thermodynamic asymmetry in dynamic chemistry
Source: Nat Commun. 2026 Oct 7;17:10574. doi: 10.1038/s41467-026-78292-4 (PMC13645739; doi:10.1038/s41467-026-78292-4)
Supplement: Supplementary file 2 [file 41467_2026_78292_MOESM2_ESM.pdf]

nature portfolio

Peer Review File

Post-fabrication Reconfiguration of Hydrogels through Thermodynamic Asymmetry in Dynamic Chemistry

Corresponding Author: Professor Andreas Walther

This file contains all reviewer reports in order by version, followed by all author rebuttals in order by version.

Attachments originally included by the reviewers as part of their assessment can be found at the end of this file.

Version 0:

Reviewer comments:

Reviewer #1

(Remarks to the Author)
This article by Walther and co-workers deals with the reconfiguration of hydrogels exploiting the different thermodynamic stability of -CH=N-R bond, which in turn, depends on the nature of R. In particular, they leverage on the more stable oxime bond with respect to hydrazone one. After a kinetic study is performed to rationalize the dynamic behavior of the reactions at hand, the acquired knowledge is transferred to the control of the rheological properties of hydrogels based on the same chemical bonds.
I like the article and I believe that it deserves publication in Nature Communications after addressing the series of issues listed below, which can contribute to improve the quality of the manuscript.
1) the demonstration of the hydrolytic mechanism for the hydrazone to oxime transformation is rather convincing. I would also carry out some experiments in DMSO which also includes tiny water percentages (1%, 2%, 3%...). Is the reaction catalyzed under such conditions? I do not mean carrying out rigorous kinetic experiments, since under such conditions the hydrolysis step could be not pseudo-first order (water concentration could be comparable to both hydrazones and oximes), but simply seeing if the equilibration in DMSO in the presence of water is faster.
2) related to above point, at page 8, line 6 from bottom, I will substitute “via a first-order reaction” with. “via a pseudo first order reaction”.
3) page 8, line 14 from bottom, I would remove the term “associative”
4) Figure 2, there is no need to put “H2O” above the equilibrium arrows. The information can be reported in the caption. It is instead important to write the right stoichiometry. For example, the reaction on the top produces water, and I think that H2O should be put on the product side. Above the arrows of the reaction depicted in Figure 2A, I would put kobs, which related to the “transimination” from left to right (although the mechanism passes through the aldehyde).
5) I also think that the equilibrium constant Keq,Ho should be indicated somewhere over the arrows of the reactions, replacing H2O.
6) Careful, a wrong stoichiometry is reported in the second chemical equation of Figure 3B. Furthermore, in the reaction between 2-Ho and 3-Al a more complex mixture of products is expected due to statistical distribution, that is both 2-Hy and its mono hydrazone (mainly this second product) can be present in the reaction mixture.
7) I have to say that I felt the last paragraph of the article a bit off with respect to the rest of the manuscript. But of course it is my personal opinion and I can understand the authors if they leave it.
8) I think that the sentence at page 19: “To our knowledge…..” should be reformulated in a less auto-celebrative style.
9) recent ref J. Am. Chem. Soc. 2025, 147, 11327 should be cited together with ref 25.

Overall, I support the publication of the article, which I like very much, after the authors take into account the above suggestions.

Reviewer #2

(Remarks to the Author)
In this research, Campagna and Walther are trying to establish a general framework for chemically reprogramming soft materials after formation through exploiting the thermodynamic asymmetry between hydrazone and oxime linkages.

However, the dynamic covalent chemistry mentioned in this research is conventional and studied quiet a lot. Even the methodology of thermodynamic asymmetry is interesting and a good theory to demonstrate the complexity and way-of-understanding of the post-fabrication during reconfiguration of networks within matrix structure. But the generality of the design principle should be adequately confirmed in different dynamic chemistry. Additionally, the author made the statement that "While individual dynamic covalent reactions have been extensively characterized in solution, general strategies that translate molecular-level exchange into controlled, spatially accessible reconfiguration of polymer networks are scarce. Consequently, clear design principles for chemically reprogramming soft materials after formation are missing." So far as I know, quiet a few research papers in this field have investigated the topological reconfiguration of soft materials which should be introduced and cited, such as Nature Chemistry 2017, 9, 817–823; Macromolecules 2026. 59, 4, 2623; JACS. 2021, 143, 51, 21622; JACS 2020, 142 (8), 3913.
Other questions. 1) In figure 2B, some information is missing, such as concentrations for the pairs between 1-Ald and 1-Hy, 1-Ho and 1-Al, as well as the justification of reaction times, such 14 h and 40 h for these particular reactions. 2) More pH effect in Figure 4 should be studied which play very an important role during this particular dynamic covalent bonding exchange. 3) The reason of choosing the fluorescein as the fluorophore in Figure 5B is not clear since it is a typical ACQ (aggregation caused quenching) dye. 4) BTW, more images for the reconfigured gels should be displayed in Figure 5 which can demonstrate the states of different ones during the transitions.

Reviewer #3

(Remarks to the Author)
See attached review

Version 1:

Reviewer comments:

Reviewer #1

(Remarks to the Author)
I congratulate the authors for the job done in revising the manuscript. They fully clarified my concerns, and, in my opinion, they also well addressed the points raised by the other Reviewers.
I strongly support the publication of the manuscript in the present form.

Reviewer #2

(Remarks to the Author)
My concerns and questions have been addressed by the authors. Thus, I agree its publication at the current version.

Reviewer #3

(Remarks to the Author)
The authors have adequately revised and strengthened the manuscript in response to the reviewers' comments, including performing additional experiments for statistical analysis (triplicates). The revised version is clearer and more rigorous, and the response letter satisfactorily addresses the concerns raised in the previous review round.

Minor revisions
1. In Figure 4F, there is a red box highlighting the 24 h timepoint. Does the inset in Figure 4G correspond to this red box? If so, it seems inconsistent to include a modulus inset within a bar chart that otherwise conveys relaxation data. I suggest either moving the inset to Figure 4F or removing it altogether.
2. In the SI that the authors already indicated the unidentified signals but haven't integrated them or indicated how many % of those unidentified signals/impurities in the compound, especially in the compound 6Al page 38, and compound 8 page 39.

Reviewer #4

(Remarks to the Author)
I co-reviewed this manuscript with one of the reviewers who provided the listed reports. This is part of the Nature Communications initiative to facilitate training in peer review and to provide appropriate recognition for Early Career Researchers who co-review manuscripts.

Reviewer #5

(Remarks to the Author)
I co-reviewed this manuscript with one of the reviewers who provided the listed reports. This is part of the Nature Communications initiative to facilitate training in peer review and to provide appropriate recognition for Early Career Researchers who co-review manuscripts.

Open Access This Peer Review File is licensed under a Creative Commons Attribution 4.0 International License, which permits use, sharing, adaptation, distribution and reproduction in any medium or format, as long as you give appropriate credit to the original author(s) and the source, provide a link to the Creative Commons license, and indicate if changes were made.
In cases where reviewers are anonymous, credit should be given to 'Anonymous Referee' and the source.
The images or other third party material in this Peer Review File are included in the article's Creative Commons license, unless indicated otherwise in a credit line to the material. If material is not included in the article's Creative Commons license and your intended use is not permitted by statutory regulation or exceeds the permitted use, you will need to obtain permission directly from the copyright holder.
To view a copy of this license, visit https://creativecommons.org/licenses/by/4.0/

Dear Reviewers,

We submit the revised version of our article titled "Post-fabrication Reconfiguration of Hydrogels through Thermodynamic Asymmetry in Dynamic Chemistry".

We thank the reviewers for the constructive comments. We appreciate the opportunity to discuss the reviewers' comments and revise our manuscript accordingly. We have included new data and discussions in response to the reviewers' comments. The major changes are summarized as follows:

- **Edit of the Introduction:** We clarified the difference between conventional stimuli-responsiveness and reconfiguration of soft materials, added references for an improved assessment of the state-of-the-art regarding the reconfiguration of topology and mechanics in polymer networks, and highlighted better the key advancement of our study in the last paragraph.
- **Statistical analysis:** We provide replicates of HPLC and rheology experiments to validate statistical significance of the established correlations (Figure 2 and 3), the gel points (Figure 4), and the elastic moduli for the reconfigurations of the topology (Figure 5). Changes also in Table S1, Table S3 and Table S4.
- **Additional studies (pH effects and water content in DMSO):** We performed exchange experiments for the small molecules in DMSO, with small quantities of water, to strengthen the mechanistic framework. We determined the kinetic constant of exchange for the small molecules at pH 8 (Figure 2D), as well as the gel points and stress relaxation curves for different gels at pH 8 (Figure 4E-G).
- **Repeated experiments:** We also repeated a few experiments with proper conditions that are coherent with the rest of the work. These include new FRAP data (Figure 5C and Figure S12) and new reconfigurations (Figure S14).

Below we respond to each comment point-by-point.

We trust that our revisions comprehensively address the points raised by the reviewers. We hope that these additional experiments and the changes in the manuscript fully address all the reviewers' comments to your satisfaction, and that the manuscript is now in a position to be accepted for publication in *Nature Communications*.

Sincerely,

Andreas Walther and co-authors

Reviewer 1:

This article by Walther and co-workers deals with the reconfiguration of hydrogels exploiting the different thermodynamic stability of -CH=N-R bond, which in turn, depends on the nature of R. In particular, they leverage on the more stable oxime bond with respect to hydrazone one. After a kinetic study is performed to rationalize the dynamic behavior of the reactions at hand, the acquired knowledge is transferred to the control of the rheological properties of hydrogels based on the same chemical bonds.

I like the article and I believe that it deserves publication in Nature Communications after addressing the series of issues listed below, which can contribute to improve the quality of the manuscript.

Thank you for the positive evaluation of our manuscript.

1) the demonstration of the hydrolytic mechanism for the hydrazone to oxime transformation is rather convincing. I would also carry out some experiments in DMSO which also includes tiny water percentages (1%, 2%, 3%...). Is the reaction catalyzed under such conditions? I do not mean carrying out rigorous kinetic experiments, since under such conditions the hydrolysis step could be not pseudo-first order (water concentration could be comparable to both hydrazones and oximes), but simply seeing if the equilibration in DMSO in the presence of water is faster.

We thank the reviewers for the suggestions. We performed tests with 1 vol % and 5 vol %, observing no formation of the oxime via exchange. We added HPLC plots in Figure S6 and discussed this in the Main Text (second paragraph at page 8).

2) related to above point, at page 8, line 6 from bottom, I will substitute “via a first-order reaction” with. “via a pseudo first order reaction”.

We applied the changes as suggested.

3) page 8, line 14 from bottom, I would remove the term “associative”

We applied the changes as suggested.

4) Figure 2, there is no need to put “H2O” above the equilibrium arrows. The information can be reported in the caption. It is instead important to write the right stoichiometry. For example, the reaction on the top produces water, and I think that H2O should be put on the product side. Above the arrows of the reaction depicted in Figure 2A, I would put kobs, which related to the “transimination” from left to right (although the mechanism passes through the aldehyde).

We applied the changes in both Figure 2 and Figure 3.

5) I also think that the equilibrium constant Keq,Ho should be indicated somewhere over the arrows of the reactions, replacing H2O.

We applied the changes in Figure 2.

6) Careful, a wrong stoichiometry is reported in the second chemical equation of Figure 3B. Furthermore, in the reaction between 2-Ho and 3-Al a more complex mixture of products is expected due to statistical distribution, that is both 2-Hy and its mono hydrazone (mainly this second product) can be present in the reaction mixture.

We corrected the stoichiometry. However, we believe that showing the full mixture is not highly relevant here as the focus is on the final product. To improve clarity, we added a short comment about the formation of mono-hydrazone in the caption of Figure 3B.

7) I have to say that I felt the last paragraph of the article a bit off with respect to the rest of the manuscript. But of course it is my personal opinion and I can understand the authors if they leave it.

We decided not to modify this paragraph, since we believe it is an adequate concluding remark that contextualizes our system in a broader spectrum, serving as conceptual perspective and inspiration for future studies.

8) I think that the sentence at page 19: "To our knowledge….." should be reformulated in a less auto-celebrative style.

This was not meant to be "auto-celebrative", but to reflect our deep literature research. We nonetheless reformulated the sentence accordingly.

9) recent ref J. Am. Chem. Soc. 2025, 147, 11327 should be cited together with ref 25.

We added the suggested reference accordingly.

Overall, I support the publication of the article, which I like very much, after the authors take into account the above suggestion

Thank you very much for your encouraging assessment.

Reviewer 2:

In this research, Campagna and Walther are trying to establish a general framework for chemically reprogramming soft materials after formation through exploiting the thermodynamic asymmetry between hydrazone and oxime linkages. However, the dynamic covalent chemistry mentioned in this research is conventional and studied quiet a lot. Even the methodology of thermodynamic asymmetry is interesting and a good theory to demonstrate the complexity and way-of-understanding of the post-fabrication during reconfiguration of networks within matrix structure. But the generality of the design principle should be adequately confirmed in different dynamic chemistry. Additionally, the author made the statement that "While individual dynamic covalent reactions have been extensively characterized in solution, general strategies that translate molecular-level exchange into controlled, spatially accessible reconfiguration of polymer networks are scarce. Consequently, clear design principles for chemically reprogramming soft materials after formation are missing." So far as I know, quiet a few research papers in this field have investigated the topological reconfiguration of soft materials which should be introduced and cited, such as Nature Chemistry 2017, 9, 817–823; Macromolecules 2026. 59, 4, 2623; JACS. 2021, 143, 51, 21622; JACS 2020, 142 (8), 3913.

We thank the reviewer for the suggestions. We agree that the papers mentioned are worth being properly cited and contextualized. Indeed, we edited the corresponding part (third paragraph at page 2), accordingly, highlighting the key limitations of the works that contrast with our study. In short, these studies have drawbacks related to the conditions (harsh, heating at 120°C, which dramatically limits the applicability) or to the specificity of the exchangeable motif (which limits the array of possible reconfigurations).

Other questions. 1) In figure 2B, some information is missing, such as concentrations for the pairs between 1-Ald and 1-Hy, 1-Ho and 1-Al, as well as the justification of reaction times, such 14 h and 40 h for these particular reactions.

The concentrations in Figure 2B are the same as for the Figure 2C. To avoid confusion, we added "B" in the sentence "(Conditions for (B, C and D))". Regarding the reaction time, 14h was chosen to ensure equilibrated hydrazone formation, whereas 40h is the approximate time at which the hydrazone-to-oxime exchange is near-quantitative. We clarified these aspects in the caption of Figure 2B.

2) More pH effect in Figure 4 should be studied which play very an important role during this particular dynamic covalent bonding exchange.

We have so far performed reactions at pH = 6, 7. We have now performed additional studies at pH = 8, both for the small molecules (kinetic constants in Figure 2) and for the hydrogels. In the latter case, we added the gelation point in Figure 4D (in triplicates), as well as new stress relaxation data (Figure 4E and S11) and related gelations of hydrazone PEG2Ho and oxime PEG2Ox (direct); see Figure S10. We edited the related discussions in the Main Text between pages 5-6 (kinetics of small molecules) and in the second paragraph at page 11 (gelations).

3) The reason of choosing the fluorescein as the fluorophore in Figure 5B is not clear since it is a typical ACQ (aggregation caused quenching) dye.

We thank the reviewer for raising this point. To begin with, fluorescein is a routine dye for FRAP experiments. Fluorescein derivatives can indeed show concentration-dependent self-quenching or aggregation-caused quenching, especially at high local dye concentrations or high labeling densities. However, this effect is highly dependent on the dye derivative, pH, ionic strength, and local environment, and it is typically most pronounced in the high micromolar to millimolar concentration range (PLoS ONE, 2020, 16, e0247326). For example, reported bulk-solution studies on FITC/fluorescein-type dyes show pronounced self-quenching above ca. 0.5 mM, whereas our FRAP experiments were conducted with a final fluorophore concentration of only 0.05 mM.

More importantly, possible moderate changes in fluorescence quantum yield do not affect the central conclusion of the FRAP experiment. The diffusion coefficient was not extracted from absolute fluorescence intensity, but from the temporal broadening of the bleached intensity profile after point bleaching. Therefore, concentration-dependent brightness effects would mainly influence signal-to-noise, but not create an artificial near-zero diffusion coefficient.

This is further supported by the internal controls: free fluorescein in PBS and free fluorescein in the PEG-2Ox gel show high diffusion coefficients, whereas 5-AI incorporated during direct gelation or diffusion-exchange gives near-zero diffusion coefficients. Thus, under the experimental conditions used here, the FRAP analysis clearly distinguishes mobile from network-attached fluorophores. We therefore do not expect ACQ/self-quenching of fluorescein to influence the interpretation of the FRAP results.

4) BTW, more images for the reconfigured gels should be displayed in Figure 5 which can demonstrate the states of different ones during the transitions.

The gel is characterized by heterogeneous mechanics and softening during the transition, as reported in the *in-situ* reconfiguration with constant rheological monitoring in time sweep (now in Figure S13 due to the revision of Figure 5). Regarding the reconfiguration *ex-situ*, the gels require ca. 5 days to get a homogeneous network that results in safe handling, as shown in the image in Figure 5A. As further clarification, we added an image in Figure S13B that shows the structural integrity of the gel immediately after removal from the mold. During the transition, the gel cannot be safely removed from the mold for images due to local softening that leads to significant deformation.

Reviewer 3

Summary:

This manuscript presents a strategy for post-fabrication reconfiguration of hydrogel networks by exploiting thermodynamic asymmetry in dynamic covalent chemistry, specifically hydrazone-to-oxime exchange. The work combines small-molecule kinetic analysis with hydrogel formation and reprogramming, demonstrating that crosslink identity can be altered after gelation to modulate mechanical, topological, and functional properties. The integration of molecular-level kinetics with materials-level behavior is a strength, and the ability to achieve near-quantitative exchange within a network is compelling. The manuscript is well executed overall and addresses an important problem in adaptive materials, but several aspects of the mechanistic interpretation, rheological analysis, and framing would benefit from clarification and additional support.

Thank you very much for the positive assessment.

Major Comments:

1. The manuscript overstates the novelty of post-fabrication reconfiguration of hydrogel networks. There has been substantial prior work demonstrating that network mechanics can be modified after gelation through competitive binding, changes in pH or ionic environment, temperature, and other dynamic or stimuli-responsive chemistries. In this context, the key advance of the present work is not the general concept of post-gelation modification, but rather the ability to achieve directional and near-quantitative replacement of covalent crosslinks through thermodynamic bias. Clarifying this distinction and citing representative prior work would improve the accuracy of the framing and better highlight the true contribution of the study.

We thank the reviewer for the insightful input. We edited the first paragraph of the introduction, accordingly, highlighting the distinction between stimulus-responsive behaviour and reprogramming the network. While the first involves approaches that modulate the existing network, the second scenario implies exchanging molecular motifs that constitute the network to alter topology, mechanics or function.

We also made a few smaller edits. In the third paragraph of the introduction, we start by stating “Achieving more complex reprogramming requires more than reversibility”, putting emphasis on the reprogramming concept rather than on the more trivial and generic “post-fabrication reconfiguration”. Additionally, we edited the last paragraph, focusing on the mode of reconfiguration that allows the reprogramming, which indeed is the “near-quantitative replacement of crosslinking segments”.

2. The presentation of the kinetic data in Figure 4B/C makes the results difficult to interpret. The use of an axis break introduces a discontinuity in the time scale, and the spacing before and after the break is not consistent. In particular, the minor tick spacing changes substantially across the break (e.g., sub-hour resolution before the break versus multi-hour intervals after), making it difficult to directly compare rates or assess the evolution of the system across the full time range. Because these data are central to establishing the exchange kinetics, the current visualization limits interpretability. Reformatting the figure using a continuous log-scale axis or an inset for early time points would significantly improve clarity.

Figure 4 was significantly revised also considering modifications according to other comments (*e.g.*, statistical analysis, additional data like pH 8). In the current state, the axis brake has been removed to improve clarity, the gelation by exchange has been moved in a separated graph, and the full gelations of PEG4Ox (exchange and competition) have been moved to Figure S10, making the Figure 4 looking more ordered. Gel point data are reported in Figure 4D for the quantitative evaluation. Due to these modifications of the Figures, we applied related changes in the Main Text in the last paragraph of page 10 and in the first paragraph of page 11.

3. The stress relaxation analysis requires more careful treatment. The relaxation experiments appear to be conducted over a time window up to ~10^5 s, yet several reported relaxation times (e.g., PEG-2Ox direct, PEG-2Ox exchange, PEG-4Ox direct in Table S3) extend beyond this range, indicating that these values are extrapolated rather than directly measured. In addition, it appears that different samples were measured over different time windows, which complicates comparison across conditions. The long experimental durations (up to ~1 day) also raise concerns about potential evaporation or drift, even with the use of an oil layer. Finally, a single-exponential Maxwell model does not appear appropriate for all systems, particularly the slower-relaxing oxime networks. Incorporating a stretched exponential (KWW) analysis and reporting both τ and the stretching exponent (β) would provide a more complete description of the relaxation behavior and may reveal meaningful differences between networks.

The stress relaxation data was largely revised. We decided to perform relaxation until a maximum of 24 hours. We therefore calculated characteristic relaxation times for the curves that are fully or almost fully relaxed, whereas, for the very slowly relaxing samples, we calculated the fraction of modulus at 24 h versus the initial modulus as alternative, extrapolation-free quantitative evaluation (see Figure 4F-G). All the values are reported in Table S3 (which was updated accordingly). We edited the related discussion in the Main Text at page 13, accordingly.

To prevent evaporation, we routinely use silicon oil (medium viscous, 350 cSt) to cover any gel/air interfaces. This works well for days. Hydrogels are very well preserved in these conditions, and the fitting with a single exponential confirms accuracy.

We performed a comparison between single exponential and stretched exponential decay as shown in the Figure below. No significant difference can be detected, as the exponential parameter *β* converges to 1. This implies that the single exponential decay is an adequate model. Since Nature Communications operates with a transparent peer review scheme, we prefer to only list this comparison here for an interested reader.

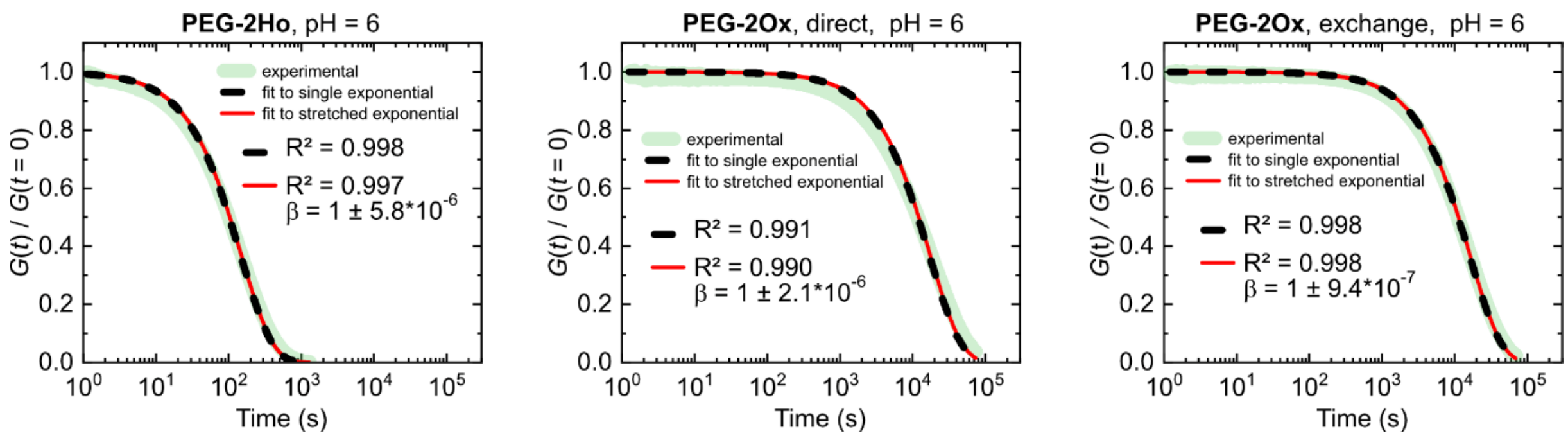

4. The manuscript relies heavily on thermodynamic arguments to explain the observed changes in network properties, but these are not fully supported experimentally. While the equilibrium constant for hydrazone formation/hydrolysis (Keq,Ho ≈ 0.74 M^{-1}) is reported, the corresponding equilibrium for the oxime system is not measured, despite being invoked to explain differences in final modulus (e.g., higher G′ attributed to higher oxime stability). In addition, the current definition of Keq may be confusing, as it is presented in a dissociative form in which smaller values correspond to stronger association. Clarifying this definition (e.g., using Ka vs Kd) and providing a more complete set of thermodynamic and kinetic parameters (including oxime hydrolysis or stability where relevant) would strengthen the mechanistic framework.

We thank the reviewer for the suggestion regarding the nomenclature of the equilibrium constant. We edited the Main Text and the Supplementary Information accordingly, and also Figures 3 and Figure S9.

Regarding the identification of the equilibrium constant for the oxime system, there are some aspects that require a more detailed discussion. Unlike the hydrazone system, analysis by UV-Vis is not possible due to the lack of an absorption band at 240 nm for the oxime. Analytic HPLC would be too unprecise at the low concentration required to reliably detect very little fractions of the free alkoxyamine. The most important work (cited in our manuscript) about the investigation of the kinetic constants of hydrolysis of hydrazones and oxime (Angew. Chem. 2008, 47, 7523) relies on NMR in deuterated buffer. In this work, the authors do not simply detect the fraction of alkoxyamine at equilibrium, but they use a specific trap to push the system towards complete hydrolysis. This technique allows to detect large concentrations of the hydrolyzed state, necessary for a reliable NMR integrals analysis. However, we did not purse such a detailed investigation of the oxime hydrolysis for two main reasons: 1) the mentioned work consistently reports a higher hydrolytic stability of the oxime, which represents a sufficient information for us to develop the kinetic framework that we present in our work; 2) in the mentioned work, the authors synthesize specific hydrazones and oxime using pivalaldehyde, which “was chosen because it lacks enolizable protons, thus preventing obfuscating side reactions such as aldol condensations”. Our model aldehyde (1-Ald) has enolizable protons, implying that side reactions would affect a precise determination of the kinetics parameters by NMR.

In our study, the central mechanistic question is not the absolute hydrolysis constant of a specific oxime, but whether the hydrazone-to-oxime exchange can be described by a hydrolytically mediated pathway and whether this thermodynamic bias is sufficient to drive near-quantitative network reconfiguration. For this purpose, the established and consistently reported higher hydrolytic stability of oximes relative to hydrazones provides the relevant thermodynamic boundary condition.

Moreover, extracting an apparent oxime hydrolysis constant in our specific system would likely introduce uncertainty rather than strengthen the interpretation. Reliable quantification would require trapping or forcing hydrolysis to generate detectable amounts of the free alkoxyamine, as done in previous detailed small-molecule studies. Such an experiment would probe a modified chemical system rather than the actual network-reconfiguration conditions used here. We therefore avoid assigning an insufficiently constrained oxime equilibrium constant and instead base our mechanistic analysis on the experimentally measured hydrazone equilibrium, the observed near-quantitative exchange to oxime, and literature-established oxime hydrolytic stability.

We also recognize that the kinetic model necessarily involves simplifying assumptions, including negligible oxime hydrolysis on the experimental time scale. We however point to these aspects clearly

in the manuscript, and are confident that these aspects shine through and are sufficiently addressed in the manuscript.

5. The mechanistic conclusion that hydrazone-to-oxime exchange proceeds predominantly through a hydrolytic pathway is reasonable but not fully resolved. The absence of exchange in DMSO is used to argue against a direct associative pathway, but this may reflect kinetic suppression rather than complete exclusion. In addition, the mechanism relies on inferred aldehyde intermediates and fitted rate constants derived from multiple techniques (UV–Vis for k_1/k_{-1} and HPLC for k_1), along with simplifying assumptions such as negligible oxime hydrolysis. While the proposed mechanism is consistent with the data, the conclusions should be framed more carefully given these limitations.

We thank the reviewer for the comment. We agree that the available data do not formally exclude a minor contribution from a direct pathway under all possible conditions. However, we indeed wrote "Since DMSO suppresses hydrolytic pathways…" in the second paragraph at page 8, which, as the reviewer commented, does not imply complete exclusion. On page 10, we have now included a longer final sentence to highlight this more clearly as a final note.

We also recognize that the kinetic model necessarily involves simplifying assumptions, including negligible oxime hydrolysis on the experimental time scale. We however point to these aspects clearly in the manuscript, and are confident that these aspects shine through and are sufficiently addressed in the manuscript.

6. The exchange chemistry and resulting material properties are highly sensitive to environmental conditions, particularly pH and buffer composition, but this is not fully developed in the manuscript. For example, in the Fe^{3+}–catechol reconfiguration experiments, the system is transferred from buffered conditions to DI water, where pH is not controlled. Both oxime/hydrazone exchange and Fe^{3+}–catechol coordination are strongly pH-dependent, making it difficult to determine whether the observed changes in viscoelastic response arise from metal–ligand interactions, changes in dynamic covalent exchange, or both. Similarly, reported pH values for different gel compositions (e.g., PEG-2Ho ~8.3 vs PEG-2Ox ~5.4) suggest significant variation that could influence both kinetics and mechanics. A clearer treatment of how pH is controlled and how it influences the observed behavior would strengthen the interpretation.

The pH of deionized water and stock solution of crosslinkers (2-Hy and 7-Al), used to fabricate the initial gel and to perform the reconfiguration, were carefully adjusted to the same value (pH 7.5). Once the 7-Al is combined with the hydrazone in the gel, the exchange reaction takes place, which has a negligible influence on the pH. We edited the last section of page 13 in the Supplementary Information accordingly.

Regarding other experiments with different compositions, we repeated those experiments using 100 mM PBS and the pH of all the solutions was properly adjusted to the same desired value. This involves the reconfiguration in vial (entries 1-3 in Table S4, and Figure S14, with new images) and the samples used for the FRAP analysis (Figure 5C and S12). We also edited the sections "FRAP experiments" and "topological reconfiguration" in the Supplementary Information accordingly.

7. The manuscript would also benefit from stronger statistical support. Kinetic parameters are derived from fitted datasets without replicate measurements, and rheological data (e.g., differences in modulus attributed to dangling chains in Figure 5) are presented without error bars despite relatively small differences between conditions. Given the potential variability in both kinetics and rheology, including replicate measurements or uncertainty estimates would improve confidence in the conclusions.

We have now performed additional extensive experiment for statistical analysis (triplicates) of kinetics studies by HPLC (Figure 2-3, Table S1), gelations (Figure 4, Table S3), and reconfigurations (Figure 5, Table S4). In all cases, the new data are in line with the ones reported previously.

For the topological reconfiguration, we did a more substantial revision besides the statistical analysis. We made a new pictogram that underscores the dangling chains responsible for the modulation of the mechanics (Figure 5D). Since we now report averages and standard deviations in Figure 5E, we moved the full *in-situ* rheology to Figure S13, together with the short discussion about the transient drop of *G*' (see note below Figure S13). Additionally, we repeated the experiment for the reconfiguration from a stiffer hydrazone gel PEG-2Ho to a softer oxime gel PEG-2Ox, using different conditions (now 1.45 eq. of crosslinker 2-Al) for a better overview of the modulation of mechanics. This involves changes in Figure 5E and a new stress relaxation curve in Figure 5H (PEG-2Ox). Overall, we edited the Main Text, according to these changes, on page 16.

Minor comments:

1. The NMR data in the Supporting Information would benefit from improved clarity and consistency. Several peak integrations (e.g., Compound 2) appear inconsistent with expected values, some spectra are truncated such that key regions (e.g., ~1.4 ppm for Boc groups) cannot be verified, and residual solvent peaks (e.g., MeOH, EtOAc) are not consistently labeled. In addition, some spectra (e.g., Compound 5) contain unidentified impurity peaks that should be annotated or discussed. Presenting full spectral windows (e.g., down to ~0.5 ppm) and consistent labeling across all spectra would improve reproducibility.

We performed the following corrections: Integration of compound 2; for compounds 3-Al, 5-Al, and 8, the region until 0.5 ppm is now visible; for compound 4 the signals of EtOAc and impurities are now indicated; for compound 5 the impurities are now indicated and commented; for compound 6-Al unidentified signals are now indicated; for compound 7 the solvent signals are now indicated.

2. Additional detail is needed for the rheological measurements. The gap size used in the experiments should be specified, and the duration of individual gelation and stress relaxation experiments should be clearly reported. It would also be helpful to clarify why different samples were measured over different time windows.

We added the requested details as second paragraph in the section of the Supplementary Information "Gelations experiments in-situ: direct, exchange, competitions".

3. The rheological characterization focuses primarily on time sweeps and stress relaxation. Including representative frequency sweeps would allow reporting of plateau modulus and crossover

frequencies, providing an independent estimate of relaxation times and enabling comparison with values obtained from stress relaxation (e.g., $\tau \approx 147$ s for PEG-2Ho at pH 6).

We performed frequency sweeps for representative fast relaxing hydrazone gels (pH7 with catalyst and pH6) and compared the relaxation times with the ones obtained by fitting the stress relaxation curves. The values extracted using these two techniques are in the same order of magnitude (see Figure S11 and related note).

4. The presentation of kinetic parameters could be clarified. Rate constants are derived from different experimental techniques and combined in the modeling, and simulated “low/high” parameter sets are presented alongside experimentally derived values. Clearly distinguishing measured versus modeled parameters would improve transparency.

To improve clarity, we extended the caption of Table S2 to explicitly mention which are the parameters determined experimentally, and which are chosen arbitrarily.

5. The reporting of pH throughout the manuscript should be clarified. It is not always clear how pH was measured, particularly in gel samples, and some reported values are not immediately intuitive based on composition. Explicitly stating when pH was adjusted (e.g., in Tables S1 and S3) and how it was measured would improve clarity.

The pH of any used solution was carefully adjusted to the same desired value (see “control of pH” in materials and methods). Regarding the specific experiments, we have now added more detailed descriptions below Table S1 and S3, and in the last three sections on page 13 in the Supplementary Information. As explained below Table S1, we verified that the pH of buffer without dissolved PEG polymer is nearly identical to the pH after dissolving the polymer. Hence, we consider the pH of the final gel to have a value nearly identical to those of the used solutions (buffer, crosslinkers stock solutions, other eventual reactants such as DTT, all adjusted at the same values prior to combining aliquots).

Reviewer 3 – Minor revisions

1. In Figure 4F, there is a red box highlighting the 24 h timepoint. Does the inset in Figure 4G correspond to this red box? If so, it seems inconsistent to include a modulus inset within a bar chart that otherwise conveys relaxation data. I suggest either moving the inset to Figure 4F or removing it altogether.

We thank the reviewer for the suggestion. We have removed the inset from the Figure. In the related discussion in the main text (page 6), we now refer to Table S3 (which contains all tabulated values) instead of Figure 4.

2. In the SI that the authors already indicated the unidentified signals but haven't integrated them or indicated how many % of those unidentified signals/impurities in the compound, especially in the compound 6Al page 38, and compound 8 page 39.

We thank the reviewer for the comments. We have now included newly recorded NMR spectra of compounds 6-Al and 8 using fresh deuterated solvent. Impurities are visibly reduced. Overall, impurities are always only a truly minor fraction of the total integration, therefore we believe it is out of scope to determine individual integrals for all the observable traces of impurities in the compounds spectra.

Summary:

This manuscript presents a strategy for post-fabrication reconfiguration of hydrogel networks by exploiting thermodynamic asymmetry in dynamic covalent chemistry, specifically hydrazone-to-oxime exchange. The work combines small-molecule kinetic analysis with hydrogel formation and reprogramming, demonstrating that crosslink identity can be altered after gelation to modulate mechanical, topological, and functional properties. The integration of molecular-level kinetics with materials-level behavior is a strength, and the ability to achieve near-quantitative exchange within a network is compelling. The manuscript is well executed overall and addresses an important problem in adaptive materials, but several aspects of the mechanistic interpretation, rheological analysis, and framing would benefit from clarification and additional support.

Major Comments:

1. The manuscript overstates the novelty of post-fabrication reconfiguration of hydrogel networks. There has been substantial prior work demonstrating that network mechanics can be modified after gelation through competitive binding, changes in pH or ionic environment, temperature, and other dynamic or stimuli-responsive chemistries. In this context, the key advance of the present work is not the general concept of post-gelation modification, but rather the ability to achieve directional and near-quantitative replacement of covalent crosslinks through thermodynamic bias. Clarifying this distinction and citing representative prior work would improve the accuracy of the framing and better highlight the true contribution of the study.

2. The presentation of the kinetic data in Figure 4B/C makes the results difficult to interpret. The use of an axis break introduces a discontinuity in the time scale, and the spacing before and after the break is not consistent. In particular, the minor tick spacing changes substantially across the break (e.g., sub-hour resolution before the break versus multi-hour intervals after), making it difficult to directly compare rates or assess the evolution of the system across the full time range. Because these data are central to establishing the exchange kinetics, the current visualization limits interpretability. Reformatting the figure using a continuous log-scale axis or an inset for early time points would significantly improve clarity.

3. The stress relaxation analysis requires more careful treatment. The relaxation experiments appear to be conducted over a time window up to ~10^5 s, yet several reported relaxation times (e.g., PEG-2Ox direct, PEG-2Ox exchange, PEG-4Ox direct in Table S3) extend beyond this range, indicating that these values are extrapolated rather than directly measured. In addition, it appears that

different samples were measured over different time windows, which complicates comparison across conditions. The long experimental durations (up to ~1 day) also raise concerns about potential evaporation or drift, even with the use of an oil layer. Finally, a single-exponential Maxwell model does not appear appropriate for all systems, particularly the slower-relaxing oxime networks. Incorporating a stretched exponential (KWW) analysis and reporting both τ and the stretching exponent (β) would provide a more complete description of the relaxation behavior and may reveal meaningful differences between networks.

4. The manuscript relies heavily on thermodynamic arguments to explain the observed changes in network properties, but these are not fully supported experimentally. While the equilibrium constant for hydrazone formation/hydrolysis ($K_{eq,Ho} \approx 0.74\ M^{-1}$) is reported, the corresponding equilibrium for the oxime system is not measured, despite being invoked to explain differences in final modulus (e.g., higher G′ attributed to higher oxime stability). In addition, the current definition of Keq may be confusing, as it is presented in a dissociative form in which smaller values correspond to stronger association. Clarifying this definition (e.g., using Ka vs Kd) and providing a more complete set of thermodynamic and kinetic parameters (including oxime hydrolysis or stability where relevant) would strengthen the mechanistic framework.

5. The mechanistic conclusion that hydrazone-to-oxime exchange proceeds predominantly through a hydrolytic pathway is reasonable but not fully resolved. The absence of exchange in DMSO is used to argue against a direct associative pathway, but this may reflect kinetic suppression rather than complete exclusion. In addition, the mechanism relies on inferred aldehyde intermediates and fitted rate constants derived from multiple techniques (UV–Vis for $k_□/k_{-□}$ and HPLC for $k_□$), along with simplifying assumptions such as negligible oxime hydrolysis. While the proposed mechanism is consistent with the data, the conclusions should be framed more carefully given these limitations.

6. The exchange chemistry and resulting material properties are highly sensitive to environmental conditions, particularly pH and buffer composition, but this is not fully developed in the manuscript. For example, in the Fe^{3+}–catechol reconfiguration experiments, the system is transferred from buffered conditions to DI water, where pH is not controlled. Both oxime/hydrazone exchange and Fe^{3+}–catechol coordination are strongly pH-dependent, making it difficult to determine whether the observed changes in viscoelastic response arise from metal–ligand interactions, changes in dynamic covalent exchange, or both. Similarly, reported pH values for different gel compositions (e.g., PEG-2Ho ~8.3 vs PEG-2Ox ~5.4)

suggest significant variation that could influence both kinetics and mechanics. A clearer treatment of how pH is controlled and how it influences the observed behavior would strengthen the interpretation.

7. The manuscript would also benefit from stronger statistical support. Kinetic parameters are derived from fitted datasets without replicate measurements, and rheological data (e.g., differences in modulus attributed to dangling chains in Figure 5) are presented without error bars despite relatively small differences between conditions. Given the potential variability in both kinetics and rheology, including replicate measurements or uncertainty estimates would improve confidence in the conclusions.

Minor comments:

1. The NMR data in the Supporting Information would benefit from improved clarity and consistency. Several peak integrations (e.g., Compound 2) appear inconsistent with expected values, some spectra are truncated such that key regions (e.g., ~1.4 ppm for Boc groups) cannot be verified, and residual solvent peaks (e.g., MeOH, EtOAc) are not consistently labeled. In addition, some spectra (e.g., Compound 5) contain unidentified impurity peaks that should be annotated or discussed. Presenting full spectral windows (e.g., down to ~0.5 ppm) and consistent labeling across all spectra would improve reproducibility.

2. Additional detail is needed for the rheological measurements. The gap size used in the experiments should be specified, and the duration of individual gelation and stress relaxation experiments should be clearly reported. It would also be helpful to clarify why different samples were measured over different time windows.

3. The rheological characterization focuses primarily on time sweeps and stress relaxation. Including representative frequency sweeps would allow reporting of plateau modulus and crossover frequencies, providing an independent estimate of relaxation times and enabling comparison with values obtained from stress relaxation (e.g., $\tau \approx 147$ s for PEG-2Ho at pH 6).

4. The presentation of kinetic parameters could be clarified. Rate constants are derived from different experimental techniques and combined in the modeling, and simulated “low/high” parameter sets are presented alongside experimentally

derived values. Clearly distinguishing measured versus modeled parameters would improve transparency.

5. The reporting of pH throughout the manuscript should be clarified. It is not always clear how pH was measured, particularly in gel samples, and some reported values are not immediately intuitive based on composition. Explicitly stating when pH was adjusted (e.g., in Tables S1 and S3) and how it was measured would improve clarity.
